# Brillouin Light Scattering from Magnetic Excitations

**DOI:** 10.3390/ma16031038

**Published:** 2023-01-24

**Authors:** Akira Yoshihara

**Affiliations:** Faculty of Science and Engineering, Ishinomaki Senshu University, Ishinomaki 986-8580, Japan; yosihara@isenshu-u.ac.jp

**Keywords:** Brillouin light scattering, spin wave, magnetic thin film, ultrathin film, magnetic multilayer, magnetic superlattice, magnetic anisotropy, interlayer exchange coupling, nanogranular film, superparamagnetic excitation

## Abstract

Brillouin light scattering (BLS) has been established as a standard technique to study thermally excited sound waves with frequencies up to ~100 GHz in transparent materials. In BLS experiments, one usually uses a Fabry–Pérot interferometer (FPI) as a spectrometer. The drastic improvement of the FPI contrast factor over 10^10^ by the development of the multipass type and the tandem multipass type FPIs opened a gateway to investigate low energy excitations (*ħω* ≤ 1 meV) in various research fields of condensed matter physics, including surface acoustic waves and spin waves from opaque surfaces. Over the last four decades, the BLS technique has been successfully applied to study collective spin waves (SWs) in various types of magnetic structures including thin films, ultrathin films, multilayers, superlattices, and artificially arranged dots and wires using high-contrast FPIs. Now, the BLS technique has been fully established as a unique and powerful technique not only for determination of the basic magnetic constants, including the gyromagnetic ratio, the magnetic anisotropy constants, the magnetization, the SW stiffness constant, and other features of various magnetic materials and structures, but also for investigations into coupling phenomena and surface and interface phenomena in artificial magnetic structures. BLS investigations on the Fe/Cr multilayers, which exhibit ferromagnetic-antiferromagnetic arrangements of the adjacent Fe layer’s magnetizations depending on the Cr layer’s thickness, played an important role to open the new field known as “spintronics” through the discovery of the giant magnetoresistance (GMR) effect. In this review, I briefly surveyed the historical development of SW studies using the BLS technique and theoretical background, and I concentrated our BLS SW studies performed at Tohoku University and Ishinomaki Senshu University over the last thirty five years. In addition to the ferromagnetic SW studies, the BLS technique can be also applied to investigations of high-frequency magnetization dynamics in superparamagnetic (SPM) nanogranular films in the frequency domain above 10 GHz. One can excite dipole-coupled SPM excitations under external magnetic fields and observe them via the BLS technique. The external field strength determines the SPM excitations’ frequencies. By performing a numerical analysis of the BLS spectrum as a function of the external magnetic field and temperature, one can investigate the high-frequency magnetization dynamics in the SPM state and determine the magnetization relaxation parameters.

## 1. Introduction

Since the early 1960s, Brillouin light scattering (BLS) has been widely applied to study acoustic properties near ferroelectric and ferroelastic phase transitions [1]. Usually, a Fabry–Pérot interferometer (FPI) has been used as a spectrometer for BLS. For a traditional single-pass FPI, the contrast factor *C*_1_, which is defined by the ratio between the maximum transmission and the minimum transmission, was limited to about 10^3^ at most. A traditional FPI with a higher contrast factor is a dark FPI with lower transmission efficiency. For successful BLS studies, high-quality transparent samples, which have no inclusions, polished surfaces, and dimensions larger than several mm, are strongly required to reduce the elastic scattering (Rayleigh scattering: RS) intensity. Although the index-matching technique is also helpful to reduce the RS intensity, it is rather difficult to find a suitable index-matching liquid. For these reasons, BLS in 1960–1970’s was a much more difficult experiment than Raman scattering. One of the great improvements in Fabry–Pérot interferometry was achieved by the piezoelectrically scanned FPI, which can be electrically stabilized and enable setting of an appropriate spectrum accumulation time, depending on BLS signal intensity [2]. With a piezo-scanning FPI, it is rather easy to assemble a computer-controlled BLS system, including the FPI stabilization, spectrum accumulation on the computer memories, and spectrum analysis [3]. Other great improvements were the development of the multipass-type FPI and the vernier tandem multipass-type FPI by Sandercock and coworkers^2)^ in the early 1980s [4,5]. The contrast factor of a multipass FPI is given by *C_p_* = (*C*_1_)*^p^* (*p* is the total number of passes through an FPI or FPIs). The drastic improvement of the contrast factor of the multipass FPI without losing transmission efficiency has made BLS studies much easier than the BLS studies reported in 1960 through the 1970s. The vernier tandem multipass FPI can eliminate the overlapping effect with the adjacent interference-order spectrum of a traditional FPI (a multipass FPI still retains the overlapping effect), which has enabled the monitoring of overlap-free BLS spectra up to a few hundred GHz from collective excitations in various research fields of condensed matter physics [6].

## 2. Historical Survey of Brillouin Light Scattering from Spin Waves

### 2.1. Experiment

Reviews on the early stage of BLS from SWs have been already given by Borovik-Romanov and Kreines [7], Patton [8], Sandercock [9], and Grünberg [10] by the middle 1980’s. Hillebrands gives a list of publications on SW BLS up to 1999 [11].

Since the mid-1970s, the BLS technique has been intensively applied to study spin waves (SWs) from opaque surfaces. The first observation of BLS from SWs was reported by Grüenberg and Mitawe from ferromagnetic semiconductor EuO (*T*_C_ = 69 K) [12]. Sandercock and Wettling reported SW BLSs from Fe and Ni at room temperature [13]. Many SW BLS results have been subsequently reported. Readers can find them in the list of references [11]. Thanks to the developments of high-quality thin film preparation techniques, such as the sputtering technique, the MBE technique, and so on, the BLS technique has been successfully applied to study collective SWs in various types of magnetic structures (thickness of *L*), including thin films, ultrathin films, multilayers, superlattices, and artificially arranged dots and wires [14,15,16,17,18,19,20,21]. For BLS from metallic surfaces, it is important to recognize that there is an essential difference between BLS phenomena from transparent materials and from opaque surfaces. Visible laser light penetrates at most a few hundreds of angstroms from the illuminated surface due to the skin effect (in other words, the absorption effect) [22]. The skin effect strongly violates the momentum conservation law for light scattering. For transparent materials, the momentum conservation law is fully conserved during scattering process. For description of the optical property of metals (for convenience’s sake, this example is of an isotropic metal), one should introduce a complex refractive index (*n*, *κ*). Here, *n* is the real part and *κ* is the imaginary part of the refractive index. The imaginary part *κ* is usually larger than the real part *n* for visible light in metals. Then, one should take into account the large uncertainty of Δ*q*_⊥_/*q*_⊥_~2 *κ/n* in the momentum conservation law for the surface normal (perpendicular) component *q*_⊥_ of the light momentum. The in-plane (surface parallel) component *Q*_//_ of the wave vector is defined as:

(1) $$Q_{//}=\frac{2\pi }{\lambda }\left(\sin\vartheta_{in}+\sin\vartheta_{s}\right)$$

**Figure 1:**
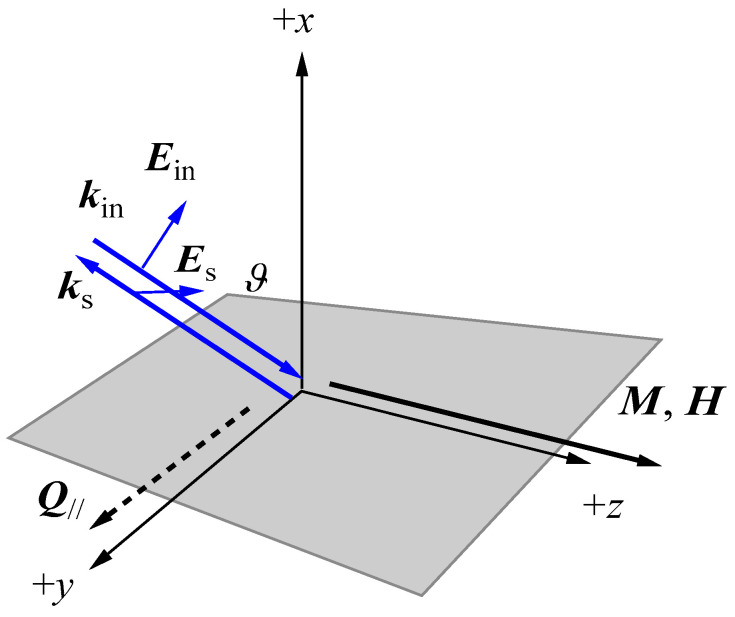
Schematic illustration of the present scattering geometry and the coordinate system for BLS spectrum calculation. The *s*-polarized component of the scattered beam is selected by using a polarizing beam splitter placed in front of the tandem FPI.

Here, *λ* is the vacuum wavelength of the laser light, and $\vartheta_{in}$ and $\vartheta_{s}$ are the incident and scattering angles measured from the surface normal. Usually, the standard backscattering geometry is employed, in which one sets $\vartheta_{in}=\vartheta_{s}=\vartheta$ as shown in Figure 1. 

In contrast to the momentum conservation law for the perpendicular component *q*_⊥_, the momentum conservation law for *Q*_//_ is always conserved, just the same as transparent materials. It is convenient for later discussions to define the surface dispersion parameter, *Q*_//_*L*.

The magnetic structures of thickness *L* are deposited on appropriate substrate. Within the skin depth of a magnetic structure, both of the SWs and surface acoustic waves (Rayleigh and the Sezawa waves) coexist and can be simultaneously observed in a BLS spectrum. For a thin magnetic structure which satisfies a condition of *Q_//_L* < 1 (though in this example, this condition is satisfied for *L* by less than a few tens of nanometers for *λ* = 5320 Å (1 Å = 10^−8^ cm = 0.1 nm) laser light and $\vartheta =45^{\circ }$), the SAW frequencies are merely controlled by the elastic properties of the substrate and independent of the external magnetic fields [23]. Hence, the SAWs are not the subjects of present interest. By examining the external magnetic field’s dependence on the observed peaks, one can readily identify the SAW peaks. As discussed later, the selection rule for SW scattering helps eliminate the SAW contributions in a BLS spectrum. Because the SWs interact with laser photons within the skin depth from the laser-illuminated surface, BLS can give us information on both bulk and surface-localized SWs. Note that the tail portion of the bulk SW within the skin depth reflects the surface’s pinning conditions for the SWs. The BLS technique can directly determine the pinning states. This is one of the most important reasons for the effectiveness of SW BLS in thin film magnetism studies. As already mentioned, the in-plane momentum conservation law (and also the energy conservation law) is satisfied, and one can expect sharp peaks for the surface-localized SWs in a BLS spectrum. On the other hand, one can expect broad bulk SW peaks due to the large uncertainty of Δ*q*_⊥_ and possibly the bulk SW dispersion from the exchange coupling.

### 2.2. Theory

Theoretical developments on SWs and SW BLSs from magnetic films were another motive force. Damon and Eshbach have already discussed magnetostatic SWs in a ferromagnetic slab and discussed the surface-localized SW now known as the Damon–Eshbach (DE) mode by employing standard magnetic boundary conditions [24]. Beyond the magnetostatic framework of the film SW theory, the dipole-exchange framework was developed [25]. In this approach, one must introduce additional boundary conditions. These are known as the Rado–Weertman boundary conditions [26] and the Hoffman boundary conditions [27]. These are related to the SW pinning effects at the interfaces.

In the late 1970s, many theoretical efforts were devoted to calculating a SW BLS spectrum from an opaque surface. Cottam developed a BLS theory for a finite-thickness ferromagnetic slab in terms of the response functions within the magnetostatic framework [28]. Another description of the BLS spectrum calculation from opaque semi-infinite ferromagnetic surfaces was published by Camley and Mills (CM) in 1978 [29]. Readers can refer to reviews by Cottam [30] and Mills [31]. Camley, Rahman, and Mills successively developed a quantitative theory for SW BLS from a ferromagnetic thin film, taking into account both the exchange coupling and the surface pinning conditions [32]. In spite of excellent agreement between the observed and calculated standing spin-wave (SSW) BLS spectra, their theory was too complicated. Another effort to calculate the SSW BLS spectrum was proposed by Cochran and Dutcher [33]. With these theoretical efforts, a quantitative comparison between the observed and calculated SW BLS spectra became possible.

Other efforts were devoted to calculating SW frequencies in thin magnetic film beyond magnetostatic approximation and in layered magnetic structures. Rado and Hicken calculated the SW frequencies from an epitaxial Fe thin film on W substrate taking into account the exchange coupling, MAE, and the surface pinning energies [34]. Grünberg discussed SWs in a trilayer, in which two magnetic layers sandwich a nonmagnetic spacer layer, by adapting the magnetostatic boundary conditions at the top and bottom surfaces and each interface [35,36]. Grünberg and Mika extended the trilayer approach to more stacked multilayer films [37]. Their approach was quite intuitive, but it requires handling a large boundary condition determinant (BCD) as the stacked multilayer increases. The transfer matrix method was developed by Barnas and found to be effective in treating the SWs in magnetic superlattices [38]. On the other hand, Camley, Rahman, and Mills developed a theory of SWs in a superlattice consisting of ferromagnetic and nonmagnetic layers within the magnetostatic framework [39]. Although the theory by Camley, Rahman, and Mills was clear in the thread of the argument and much easier to handle than the theory by Grünberg and Mika, it seems to be difficult to extend beyond the magnetostatic framework. Vohl, Barnas, and Grünberg developed a SW theory based the dipole-exchange model in which the interlayer exchange coupling between ferromagnetic layers across the nonmagnetic spacer layer was taken into account [40]. I will give brief outlines of these theories as I discuss each subject. 

For SWs in magnonic crystals, which consist of artificial periodic structures instead of mathematical descriptions, micromagnetic calculations have been widely utilized [41].

## 3. Spin Wave Light Scattering as Dynamic Magneto-Optic Effects

The most dominant interaction between laser photon and spin waves is not the Zeeman interaction but the electro-dipole interaction, which is given by [42] as below:

(2) $$H_{\mathrm{int}}=-\sum_{i,j}{\left(E_{s}\right)}_{i}\delta \varepsilon {\left(M\right)}_{ij}{\left(E_{in}\right)}_{j}$$

in which δε(M) is a magnetization-dependent dielectric constant. A phenomenological description of SW scattering with the magnetization-dependent dielectric constant was developed by Wettling, Cottam, and Sandercock [43]. For brevity’s sake, we assume there is a transparent magnet which belongs to cubic (*O*_h_) symmetry and has a dielectric constant *ε*_0_. Spontaneous magnetization $M_{z}$ is directed along the *z*-axis. Because the spontaneous magnetization appears as a result of breaking the time-reversal symmetry, not due to the symmetry lowering in the case of ferroelectrics, the dielectric tensor δε(M) should be invariant under the *O*_h_ symmetry operations. For example, we can apply the $C_{4z.}$ operation, which is *π*/2 rotation around the *z* axis, as below:

(3) $$\delta \varepsilon \left(M_{z}\right)=\left(\begin{array}{ccc} \delta \varepsilon_{xx} & \delta \varepsilon_{xy} & \delta \varepsilon_{xz}\\ \delta \varepsilon_{yx} & \delta \varepsilon_{yy} & \delta \varepsilon_{yz}\\ \delta \varepsilon_{zx} & \delta \varepsilon_{zy} & \delta \varepsilon_{zz} \end{array}\right)=\left(\begin{array}{ccc} \delta \varepsilon_{yy} & -\delta \varepsilon_{yx} & -\delta \varepsilon_{yz}\\ -\delta \varepsilon_{xy} & \delta \varepsilon_{xx} & \delta \varepsilon_{xz}\\ -\delta \varepsilon_{zy} & \delta \varepsilon_{zx} & \delta \varepsilon_{zz} \end{array}\right)=C_{4z}^{-1}\delta \varepsilon \left(M_{z}\right)C_{4z}.$$

By comparing the matrix elements before and after the operation, we can readily obtain

(4) $$\delta \varepsilon_{xx}(M_{z})=\delta \varepsilon_{yy}(M_{z})\neq \delta \varepsilon_{zz}(M_{z}),\;\delta \varepsilon_{xy}(M_{z})=-\delta \varepsilon_{yx}(M_{z})\;$$

and

(5) $$\delta \varepsilon_{xz}(M_{z})=\delta \varepsilon_{zx}(M_{z})=\delta \varepsilon_{yz}(M_{z})=\delta \varepsilon_{zy}(M_{z})=0$$

We can expand $\delta \varepsilon \left(M_{z}\right)$ into a power series of the magnetization $M_{z}$ with complex coefficients *K* and *G* as follows:

(6) $$\delta \varepsilon {\left(M_{z}\right)}_{\alpha \beta }≅\sum_{\gamma }\left({K^{\prime }}_{\alpha \beta z}+i{K^{\prime \prime }}_{\alpha \beta z}\right)M_{z}+\sum_{\gamma ,\delta }\left({G^{\prime }}_{\alpha \beta zz}+i{G^{\prime \prime }}_{\alpha \beta zz}\right)M_{z}^{2}\;\;\;\left(\alpha ,\beta ,\ldots =x,y,z\right)$$

The dielectric constant should obey the Onsager’s reciprocal theorem [43] given by, $\delta \varepsilon {\left(M_{z}\right)}_{\alpha \beta }=\delta \varepsilon {\left(-M_{z}\right)}_{\beta \alpha }$, and we obtain

(7) $$K_{\alpha \beta z}=-K_{\beta \alpha z}\;\mathrm{and}\;G_{\alpha \beta zz}=G_{\beta \alpha zz}$$

It is obvious that the diagonal elements of the dielectric matrix should be even functions of $M_{z}$, and that the expansion coefficient *K* should satisfy the following relations:

(8) $$K_{\alpha \alpha \gamma }=K_{\alpha \gamma \alpha }=K_{\gamma \alpha \alpha }=0\;\mathrm{and}\;K_{xy,z}=-K_{yx,z}$$

It is known that a second-order tensor can be decomposed into the Hermitian part ($\varepsilon_{\alpha \beta }^{H}=\varepsilon_{\beta \alpha }^{H*}$) and the anti-Hermitian part ($\varepsilon_{\alpha \beta }^{A}=-\varepsilon_{\beta \alpha }^{A*}$) as follows:

(9) $$\varepsilon_{\alpha \beta }=\frac{\varepsilon_{\alpha \beta }+\varepsilon_{\beta \alpha }^{*}}{2}+\frac{\varepsilon_{\alpha \beta }-\varepsilon_{\beta \alpha }^{*}}{2}=\varepsilon_{\alpha \beta }^{H}+\varepsilon_{\alpha \beta }^{A}$$

Here, the asterisk means the complex conjugate. Combining with the Onsager’s theorem [44], one can readily obtain

(10) $$\delta \varepsilon_{\alpha \beta }^{H}\left(M_{z}\right)=\delta \varepsilon_{\beta \alpha }^{H}{\left(M_{z}\right)}^{*}=\delta \varepsilon_{\alpha \beta }^{H}{\left(-M_{z}\right)}^{*}$$

and

(11) $$\delta \varepsilon_{\alpha \beta }^{A}\left(M_{z}\right)=-\delta \varepsilon_{\beta \alpha }^{A}{\left(M_{z}\right)}^{*}=-\delta \varepsilon_{\alpha \beta }^{A}{\left(-M_{z}\right)}^{*}$$

From Equations (6), (7), (10), and (11), one obtains

(12) $$K_{\alpha \beta ,\gamma }^{H}={K^{\prime \prime }}_{\alpha \beta ,\gamma },\;G_{\alpha \beta ,\gamma \delta }^{H}={G^{\prime }}_{\alpha \beta ,\gamma \delta },\;K_{\alpha \beta ,\gamma }^{A}={K^{\prime }}_{\alpha \beta ,\gamma },\;\mathrm{and}\;G_{\alpha \beta ,\gamma \delta }^{A}={G^{\prime \prime }}_{\alpha \beta ,\gamma \delta }.$$

Note that the real and the imaginary parts of the expansion coefficients *K* and *G* are fully separated from each other. Because we are now considering a transparent magnet, there is no optical absorption; the dielectric matrix should contain only the Hermitian components. The dielectric matrix is given as the following:

(13) $$\varepsilon \left(M_{z}\right)=\varepsilon_{0}1+\delta \varepsilon \left(M_{z}\right)=\varepsilon_{0}1+\left(\begin{array}{ccc} {G^{\prime }}_{12}M_{z}^{2} & i{K^{\prime \prime }}_{63}M_{z} & 0\\ -i{K^{\prime \prime }}_{63}M_{z} & {G^{\prime }}_{12}M_{z}^{2} & 0\\ 0 & 0 & {G^{\prime }}_{11}M_{z}^{2} \end{array}\right).$$

Here, we have introduced ${G^{\prime }}_{11}={G^{\prime }}_{zz,zz}$, ${G^{\prime }}_{12}={G^{\prime }}_{xx,zz}={G^{\prime }}_{yy,zz}$, and ${K^{\prime \prime }}_{63}={K^{\prime \prime }}_{xy,z}$ in accordance with the conventional tensor index assignment. Although Equation (13) is the fundamental equation to discuss the magneto-optic effects, it depends only on static magnetization. For discussions on SW scattering from opaque magnets, we should take into account both the contributions from the small amplitude SW variables $m_{x}$ and $m_{y}$ and from the anti-Hermitian components of the dielectric matrix as well as the Hermitian components. We can replace $M_{z}$ in Equation (6) by the magnetization vector *M* and expand Equation (6) up to the first-order terms of $m_{x}$ and $m_{y}$. In accordance with the angular momentum operators in quantum mechanics [45], we can introduce the ladder operators *m_±_* as follows: $m_{\pm }=m_{x}\pm im_{y}$. Then, the $m_{-}$ operator describes the SW creation (Stokes) process, and the $m_{+}$ operator describes the SW annihilation (anti-Stokes) process. Of course, one can introduce the magnon creation and annihilation operators through the Holstein–Primakoff representation [46]. Finally, we obtain the dielectric constant matrix, which describes SW scattering in terms of the SW operators, $m_{\pm }$, as follows:

(14) $$\delta \varepsilon_{SW}^{{}^{\left(\pm \right)}}=\frac{1}{2}\left(\begin{array}{ccc} 0 & 0 & \zeta_{13}^{\left(\pm \right)}\\ 0 & 0 & \zeta_{23}^{\left(\pm \right)}\\ \zeta_{31}^{\left(\pm \right)} & \zeta_{32}^{\left(\pm \right)} & 0 \end{array}\right)m_{\pm },$$

in which we can define:

(15) $$\zeta_{13}^{\left(\pm \right)}=\left({G^{\prime }}_{44}M_{z}\mp {K^{\prime \prime }}_{63}\right)+i\left({G^{\prime \prime }}_{44}M_{z}\pm {K^{\prime }}_{63}\right)$$

(16) $$\zeta_{23}^{\left(\pm \right)}=\left(\pm {G^{\prime \prime }}_{44}M_{z}+{K^{\prime }}_{63}\right)\mp i\left({G^{\prime }}_{44}M_{z}\mp {K^{\prime \prime }}_{63}\right)$$

(17) $$\zeta_{31}^{\left(\pm \right)}=\left({G^{\prime }}_{44}M_{z}\pm {K^{\prime \prime }}_{63}\right)+i\left({G^{\prime \prime }}_{44}M_{z}\mp {K^{\prime }}_{63}\right)$$

(18) $$\zeta_{32}^{\left(\pm \right)}=\pm \left({G^{\prime \prime }}_{44}M_{z}\mp {K^{\prime }}_{63}\right)\mp i\left({G^{\prime \prime }}_{44}M_{z}\pm {K^{\prime \prime }}_{63}\right)$$

Here we used the tensor index assignment of $G_{44}=G_{xz,xz}=G_{yz,yz}$. Based on Equation (14), we can summarize characteristic features of light scattering from SWs as follows:

1. The polarization of the SW scattered light should be cross-polarized from the polarization of the incident light. For example, we can consider the *p*-polarized incident beam in Figure 1 with the polarization vector $e_{p}=\left(\begin{array}{ccc} \sin\vartheta & -\cos\vartheta & 0 \end{array}\right)$. Then, the scattered beam should be *s*-polarized with the polarization vector $e_{s}=\left(\begin{array}{ccc} 0 & 0 & 1 \end{array}\right)$ and vice versa. Because the SAW scattering is observed in the *p*-*p* or *s*-*s* scattering geometry, we can eliminate the SAW structure from a SW BLS spectrum by inserting an analyzer in front of FPI.
2. The Stokes and anti-Stokes scattering intensities are generally different. In the above example, this can be expressed as follows:
  (19) $$\begin{array}{l} {\left|\zeta_{13}^{\left(+\right)}\right|}^{2}={\left|{G^{\prime }}_{44}M_{z}-{K^{\prime \prime }}_{63}\right|}^{2}+{\left|{G^{\prime \prime }}_{44}M_{z}+{K^{\prime }}_{63}\right|}^{2}\\ \;\;\;\;\;\;\;\;\;\neq {\left|\zeta_{13}^{\left(-\right)}\right|}^{2}={\left|{G^{\prime }}_{44}M_{z}+{K^{\prime \prime }}_{63}\right|}^{2}+{\left|{G^{\prime \prime }}_{44}M_{z}-{K^{\prime }}_{63}\right|}^{2} \end{array}$$

**Figure 2:**
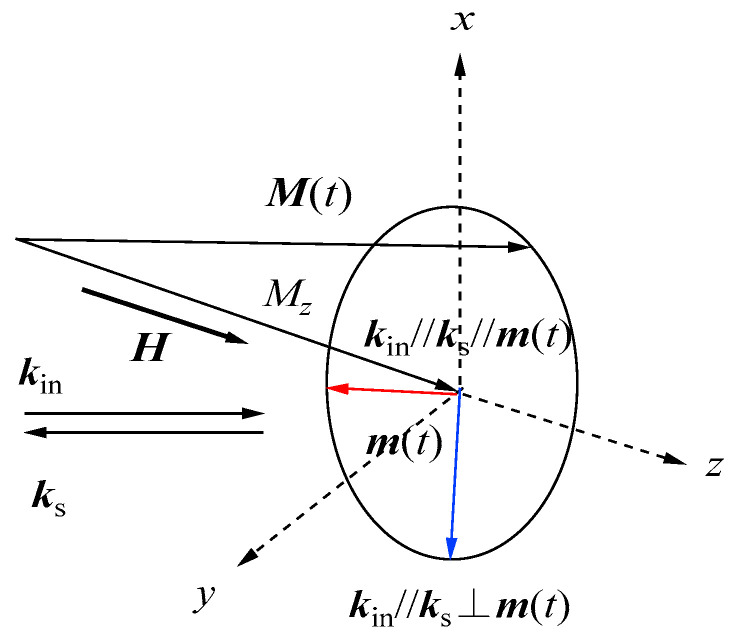
Schematic illustration of the precession motion of the magnetic moment and scattering geometry. It shows that two magneto-optical geometries take place during a cycle of precession motion.

Therefore, we can observe an asymmetrical SW spectrum around the elastic Rayleigh peak. This is in contrast with the phonon BLS spectrum, which is symmetrical around the Rayleigh peak. Furthermore, when we reverse the spontaneous magnetization $M_{z}$ to $-M_{z}$ by changing the polarity of the magnetic field, the Stokes and anti-Stokes spectra are interexchanged. Figure 2 gives a schematic illustration of the reason why the Stokes and anti-Stokes intensities are different. 

When the dynamical magnetization m(t) rotates around the static magnetization $M_{z}$, the Faraday geometry k//m(t), and the Voigt geometry k⊥m(t) that coexist during one cycle. Therefore, in SW scattering, two different magneto-optic effects simultaneously contribute to and interfere with each other. For a magnet with the real refractive index $\bar{n}$ and negligibly weak optical absorption, the real and imaginary parts of the expansion coefficients in Equation (6) can be related to the magneto-optic coefficients as follows [43]:

(20) $$\Phi_{MCB}=\frac{\pi }{\lambda \bar{n}}\Delta {\varepsilon^{\prime \prime }}_{H}=\frac{\pi }{\lambda \bar{n}}{K^{\prime \prime }}_{63}M_{z}:\mathrm{Magnetic}\mathrm{circular}\mathrm{birefringence},$$

(21) $$\Phi_{MCD}=\frac{4\pi }{\lambda \bar{n}}\Delta {\varepsilon^{\prime }}_{A}=\frac{4\pi }{\lambda \bar{n}}{K^{\prime }}_{63}M_{z}{}_{z}:\mathrm{Magnetic}\mathrm{circular}\mathrm{dichroism},$$

(22) $$\Psi_{MLB}=\frac{2\pi }{\lambda \bar{n}}\Delta {\varepsilon^{\prime }}_{H}=\frac{2\pi }{\lambda \bar{n}}{G^{\prime }}_{12}M_{z}^{2}:\mathrm{Magnetic}\mathrm{linear}\mathrm{birefringence},$$

(23) $$\Psi_{MLD}=\frac{4\pi }{\lambda \bar{n}}\Delta {\varepsilon^{\prime \prime }}_{A}=\frac{4\pi }{\lambda \bar{n}}{G^{\prime \prime }}_{12}M_{z}^{2}:\mathrm{Magnetic}\mathrm{linear}\mathrm{dichroism}.$$

3. Because the matrix components $\zeta_{13}^{\left(\pm \right)}$ and $\zeta_{23}^{\left(\pm \right)}$ are not equal to the $\zeta_{31}^{\left(\pm \right)}$ and $\zeta_{32}^{\left(\pm \right)}$ components, the *p*→*s* scattering and the *s*→*p* scattering intensities will be different in general. Because of the Brewster angle, the *p*-polarized incident arrangement is preferable to the *s*-polarized incident arrangement in Figure 1.

Quantum mechanical description of the coupling coefficient *K* was given by Fleury and Loudon [46]. Hereafter, we use the *ħ* = 1 unit for convenience’s sake. In accordance with their theory, let us consider a very simple example of a 3d electron magnetic crystal placed in the electromagnetic radiation specified by the vector potential *A*. We assume that the ferromagnetic ground state is specified by *L* = 0 and *S* = 1/2, and we also assume the *L* = 1 and *S* = 1/2 intermediate states. We took into account the orbital quenching effect at the ground state. The intermediate states split into a *J* = 3/2 quartet and a *J* = 1/2 doublet with an energy separation of 3*ζ*/2 due to the spin-orbit coupling *ζL*·*S*. The Hamiltonian interaction between an electron at *r* within a magnetic ion and the uniform or long wavelength radiation in second quantized notation is given as follows:

(24) $$H^{\prime }=-er\cdot E=er\cdot \frac{\partial A}{\partial t}\propto \sum_{\lambda }\left(r\cdot \varepsilon_{\lambda }\right)\left[a_{\lambda }^{+}\exp\left(i\omega_{\lambda }t\right)-a_{\lambda }\exp\left(-i\omega_{\lambda }t\right)\right]$$

where *a* and *a*^+^ are the photon annihilation and creation operators, $\varepsilon_{\lambda }$ is the polarization vector of the radiation, and we omitted the coefficient of the vector potential, which is not important for our discussion. We use the $|L,L_{z}\rangle |S,S_{z}\rangle$ notation for electron wave functions. The wave functions for the ground state and for the spin excited state are given by |G〉=|0,0〉|1/2,1/2〉 and $|G^{*}\rangle =|0,0\rangle |1/2,-1/2\rangle$, respectively. The intermediate wave functions responsible for the dipole transition are given by [45] as follows:

(25) $$\Phi_{3/2\;1/2}=\sqrt{\frac{2}{3}}|1,0\rangle |1/2,1/2\rangle +\sqrt{\frac{1}{3}}|1,1\rangle |1/2,-1/2\rangle$$

and

(26) $$\Phi_{3/2\;-1/2}=\sqrt{\frac{1}{3}}|1,-1\rangle |1/2,\;1/2\rangle +\sqrt{\frac{2}{3}}|1,0\rangle |1/2\;,-1/2\rangle$$

for the quartet, and

(27) $$\Phi_{1/2\;1/2}=-\sqrt{\frac{1}{3}}|1,0\rangle |1/2\;,1/2\rangle +\sqrt{\frac{2}{3}}|1,1\rangle |1/2,-1/2\rangle$$

and

(28) $$\Phi_{1/2\;-1/2}=-\sqrt{\frac{2}{3}}|1,-1\rangle |1/2,1/2\rangle +\sqrt{\frac{1}{3}}|1,0\rangle |1/2,-1/2\rangle$$

for the doublet, respectively. The photon state changes from the initial state given by $|n_{1}\rangle |n_{2}\rangle$ to the final state given by $|n_{1}-1\rangle |n_{2}+1\rangle$. Here, subscripts 1 and 2 refer to the incident and scattered field quantities. The transition from the |G〉 state to the $|G^{*}\rangle$ state is interpreted as the SW creation process (the Stokes process). We can then perform perturbation calculations on the SW creation process with these wave functions and the Hamiltonian interaction, and obtain the final result given by [22,42,47] as below:

(29) $$\frac{e^{2}\zeta }{2\sqrt{2}}\langle 00\left|z\right|10\rangle \langle 1-1|x-iy|00\rangle \left[\varepsilon_{1z}\varepsilon_{2+}-\varepsilon_{1+}\varepsilon_{2z}\right]\;\left[\;\frac{1}{{\left(\varepsilon_{\mathrm{CF}}-\omega_{1}\right)}^{2}}-\frac{1}{{\left(\varepsilon_{\mathrm{CF}}+\omega_{2}\right)}^{2}}\right]$$

where *ε*_CF_ is the crystal field splitting energy given by *ε*_J=1/2_ − *ε*_G_. It is important to recognize that the spin-orbit coupling constant *ζ* is the key parameter to determine the scattering efficiency from a 3d electron magnet. 

## 4. Experimental

The experimental setup for SW BLS is a rather standard one for conventional BLS studies with a 3 + 3 pass tandem FPI, except for the backscattering geometry. Figure 1 shows a standard scattering geometry for SW BLS and the coordination system employed in our SW calculations. The magnetic field *H* is applied along the *z*-direction within the plane of the sample and perpendicular to the *x*-*z* scattering plane defined by the incident and scattered light beams. We always measure the SWs propagating perpendicular to the magnetic field. We can employ three different scattering geometries: (A) magnetic fields applied in the in-pane easy direction (shown in Figure 1), (B) magnetic fields applied in the in-plane hard axis, and (C) a constant magnetic field rotated from the easy direction to the hard direction. Note that the magnetization ***M*** and the external magnetic field ***H*** are not collinear with each other in the (B) and (C) geometries. The incident angle ϑ is measured from the surface normal along the *x* direction and is chosen to be the same as the scattered angle. The incident angle ϑ can be arbitrarily changed between 25° and 65°. The scattering geometries (B) and (C) can be employed for the in-plane MAE studies. In general, scattering intensity from the surface SW mode increases for larger incident angles, and in contrast, the bulk SW mode intensities increase for smaller angles. 

At the early stage of our SW BLS studies, we used a laboratory-constructed Sandercock-type 3-pass (or 5-pass) FPI as an interferometer, depending on the surface quality of the prepared films. The FPI was assembled at the machine shop of the Research Institute for Scientific Measurements (RISM), Tohoku University [48,49]. For some sputtered films, we observed SW spectra by using the 3-pass FPI [50,51,52]. The spectra were excited by the 5145 Å or 4880 Å line of an argon ion laser in single-mode operation and detected by a thermoelectrically cooled photomultiplier tube (PMT) for a dark count of less than 1 cps. Later, we constructed a Sandercock-type 3 + 3 pass vernier-tandem FPI in 1994 at the machine shop of RISM, Tohoku University [53], and the multipass type FPI was replaced by the tandem FPI. The 5145 Å or 4880 Å line of argon ion laser can be replaced by the 5320 Å or 4730 Å line of diode-pumped solid-state (DPSS) single-mode laser. Various types of DPSS laser are now commercially available. The DPSS laser is much easier to use and also more economical compared with a water-cooled argon ion laser. The PMT can be also replaced by an avalanche photo-diode (APD) detector, which possesses higher quantum efficiency than the PMT detectors. 

In our system, a laser beam was introduced by a 45° right-angle prism with 3 × 3 mm^2^ input-face into an optical axis of the FPI. A camera lens (50 mm *f*1.2) focused the beam on the sample and also collected the backscattered light. We used a spatial filter that consisted of two camera lenses (135 mm *f*2.8 and 50 mm *f*1.2) and a 200 μm pinhole. To eliminate the scattering from the SAWs and most of the intense Rayleigh peaks and FPI ghost peaks, a cross-polarizing beam splitter (extinction ratio = 1/200) was inserted in front of the FPI. We found that the elastically scattered light was still intense enough in many cases even after the polarization selection. In order to protect the highly sensitive PMT or APD from optical damage due to elastically scattered light and ghosts, we introduced a tandem acousto-optic modulator (AOM), which was activated around the Rayleigh and ghost peaks as an intensity attenuator. We also added a mechanical shutter, which was activated only when the peak intensity was getting higher than a preset level.

In some cases, we experienced that a sample exposed to an intense laser beam (even less than 50 mW) in air was easily damaged by the local heating and oxidizing effects. Therefore, we found that we should place the sample inside a vacuum chamber or under an appropriate atmosphere during BLS measurements. In order to make possible the degree of atmosphere control and also the low-temperature studies under magnetic fields, we prepared a liquid He cryostat which could be used as a vacuum chamber. Furthermore, a closed-cycle refrigerator was used to generate low temperatures down to 15 K. In order to perform variable-temperature and magnetic field studies, we assembled a refrigerator tip suitable for BLS study under magnetic fields of up to 4.5 kOe at the RISM machine shop. During a spectral accumulation time over several hours, the lowest temperature of 15 K could be fully stabilized within ±0.5 K. 

## 5. BLS Results

### 5.1. Semi-Infinite Magnet 

**Figure 3:**
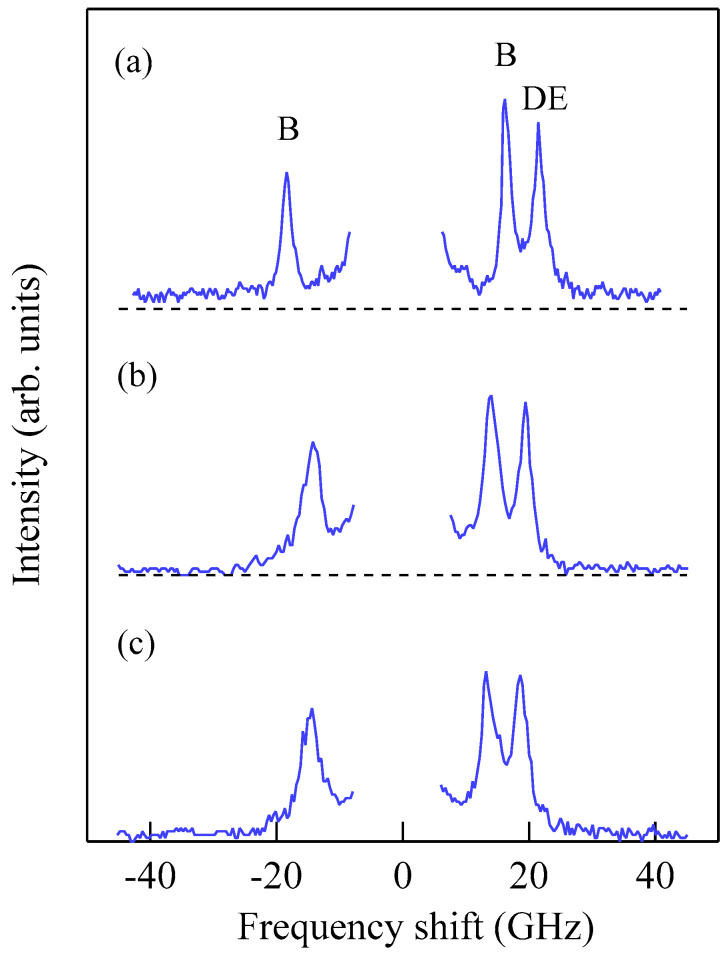
BLS spectra observed from ferromagnetic Co-Al-O nanogranular films in a magnetic field of *H* = 2.0 kOe. (**a**) Co_74_Al_18_O_18_, (**b**) Co_66_Al_11_O_23_, and (**c**) Co_63_Al_3_O_243_. The broken lines show the baselines of each spectrum [54].

Figure 3 shows typical BLS spectra obtained from nanogranular Co-Al-O films of 1~2 μm thickness prepared by means of radio frequency-reactive magnetron sputtering onto glass substrates at the Research Institute of Electric and Magnetic Materials (RIEMM) [54]. 

These spectra were excited by the *p*-polarized 4880 Å line from an Ar^+^ laser operated in a single-cavity mode with the output power below 30 mW to protect films from local heating by the laser beam. Typical spectrum accumulation time was about 4 h. An external magnetic field of *H* = 2.0 kOe was applied parallel to the film plane and perpendicular to the scattering plane (*x*-*z* plane). The incident angle was chosen to be the same as the scattered angle (*ϑ* = 45°) in these measurements. We observed very similar BLS spectra from a sputtered Fe film and from Fe-Al-O nanogranular films deposited on oxidized Si(001) substrates at the Institute of Multidisciplinary Research for Advanced Materials (IMRAM), Tohoku University. These spectra were excited by the *p*-polarized 5320 Å line from a DPSS laser [55] The TM-Al-O nanogranular films (TM = Fe, Co), which consist of crystalline TM particles with several tens of angstrom in diameter, were surrounded by the Al-O grain boundary. The Al-O grain boundary was of ~10 Å in thickness. The BLS technique observes SWs with an in-plane wavelength *λ*_//_ defined by 2*π*/*Q*_//_. The in-plane wavelength *λ*_//_ (typically ~3500 Å) is much longer than the characteristic lengths of granules but much shorter than the in-plane lengths of magnetic structures. For long-wavelength SWs observed with the BLS technique, the real magnetic structure may not be important, and the magnetic properties averaged over in-plane wavelength *λ*_//_ within the laser-illuminated area determine the SW response in BLS spectra. The peak assignment of these spectra was quite obvious as will be discussed soon. The labels DE and B refer to the Damon-Eshbach (DE) surface wave and the bulk SWs, respectively. Note that the DE peak appears only on the anti-Stokes side in these spectra in contrast to the bulk SW peaks. When we changed the polarity of the external magnetic field, the DE peak appeared on the Stokes side of a spectrum. Another interesting observation is the line shape of the bulk peaks. The bulk peaks are asymmetric with tails to higher-frequency sides. This is due to the relaxation of the momentum conservation law as already mentioned and the SW dispersion (energy as a function of the wave vector) [54]. 

In order to understand the characteristic feature of the SW spectra and the magnetic field dependence of the SW frequencies for qualitative discussions and determination of the magnetic constants, I will describe a standard magnetostatic theory rather in-detail for readers not familiar in SW BLS [22,56]. Because we are interested in the magnetization dynamics below 10^11^ Hz, which is well below the optical frequency of ~6 × 10^14^ Hz, we ignore the time-dependent terms in Maxwell’s equations. We consider a magnetic film of the magnetization *M* and thickness *L* prepared on a nonmagnetic substrate. For the sake of convenience, we can ignore the exchange coupling and the magnetic anisotropy energy (MAE) at this stage, and then set the surfaces as *x* = 0 and −*L*. Let us introduce the SW variables *m* as the small amplitude precession motion around the static magnetization ***M*** and the demagnetization field ***h***. The Landau–Lifshitz (LL) equation of motion on M(t)=M+m(t) is given as follows:

(30) $$\frac{1}{\gamma }\frac{dM}{dt}=M\times H_{eff}$$

where γ is the gyromagnetic ratio (*γ*/2*π* = 1.4*g* GHz/kOe, where *g* is the Lande’s *g* factor). The effective magnetic field $H_{eff}$ consists of the external magnetic field H and the demagnetization field ***h*** as below:

(31) $$H_{eff}=H+h$$

The demagnetization field ***h*** should satisfy Maxwell’s magnetostatic equations, given below:

(32) ∇×h=0 and ∇⋅(h+4πm)=0.

The first equation of Equation (32) guarantees the introduction of a magnetic scalar potential φ satisfying the below condition:

(33) h=−∇φ

We consider SWs propagating along the (0, cosθ,−sinθ) direction measured from the *y*-axis to be within the film plane, and we assume plane-wave-type space-time dependence for the dynamical variables:

(34) $$\left(\begin{array}{c} m\left(r,t\right)\\ \varphi \left(r,t\right) \end{array}\right)=\left(\begin{array}{c} m\\ \varphi \end{array}\right)\exp\left[i\omega t-iq_{⊥}x-i\left(Q_{//}\cos\theta \right)y+i\left(Q_{//}\sin\theta \right)z\right]$$

Furthermore, the continuity conditions of the variables *φ* and *b*_x_ = *h*_x_ + 4*πm*_x_ at x=0 and −L should be satisfied. Outside of the magnet, the scalar potentials are given

(35) $$\varphi_{\mathrm{out}}\left(r,t\right)=\varphi_{\mp }\exp\left(\mp Q_{//}x\right)\exp\left[i\omega t-i\left(Q_{//}\cos\phi \right)y+i\left(Q_{//}\sin\phi \right)z\right]$$

Here, we should choose the $\exp\left(-Q_{//}x\right)$ term for x≥0 and the $\exp\left(Q_{//}x\right)$ term for x≤−L. Inside the magnet, we have

(36) $$\begin{array}{l} \varphi_{\mathrm{in}}\left(r,t\right)=\left[\varphi \left(+\right)\exp\left(-iq_{⊥}x\right)+\varphi \left(-\right)\exp\left(iq_{⊥}x\right)\right]\\ \;\;\;\;\;\;\;\;\;\;\times \exp\left[i\omega t-i\left(Q_{//}\cos\theta \right)y+i\left(Q_{//}\sin\theta \right)z\right] \end{array}$$

We can solve the LL equation in terms of the susceptibilities:

(37) $$m_{x}\left(\omega \right)=\chi_{xx}\left(\omega \right)h_{x}+\chi_{xy}\left(\omega \right)h_{y}=\frac{-MH}{H^{2}-{\left(\omega /\gamma \right)}^{2}}\left(\frac{\partial \varphi_{\mathrm{in}}}{\partial x}\right)+\frac{iM\left(\omega /\gamma \right)}{H^{2}-{\left(\omega /\gamma \right)}^{2}}\left(\frac{\partial \varphi_{\mathrm{in}}}{\partial y}\right)$$

and

(38) $$m_{y}\left(\omega \right)=\chi_{yx}\left(\omega \right)h_{x}+\chi_{yy}\left(\omega \right)h_{y}=\frac{-iM\left(\omega /\gamma \right)}{H^{2}-{\left(\omega /\gamma \right)}^{2}}\left(\frac{\partial \varphi_{\mathrm{in}}}{\partial x}\right)+\frac{-MH}{H^{2}-{\left(\omega /\gamma \right)}^{2}}\left(\frac{\partial \varphi_{\mathrm{in}}}{\partial y}\right).$$

Combining Equations (32) and (36)–(38), we obtain

(39) $$q_{⊥}^{2}+Q_{//}^{2}+4\pi \left(Q_{//}^{2}\cos^{2}\theta +q_{⊥}^{2}\right)\chi_{xx}=0$$

The perpendicular wave vector $q_{⊥}$ is found to be

(40) $$q_{⊥}^{2}=-\frac{1+4\pi \chi_{xx}\cos^{2}\theta }{1+4\pi \chi_{xx}}Q_{//}^{2}=\frac{{\left(\omega /\gamma \right)}^{2}-H\left(H+4\pi M\cos^{2}\theta \right)}{H\left(H+4\pi M\right)-{\left(\omega /\gamma \right)}^{2}}Q_{//}^{2}$$

Because $q_{⊥}$ should be real for the bulk mode, we obtain a SW band given by

(41) $$H\left(H+4\pi M\cos^{2}\theta \right)\leq {\left(\frac{\omega }{\gamma }\right)}^{2}\leq H\left(H+4\pi M\right)$$

**Figure 4:**
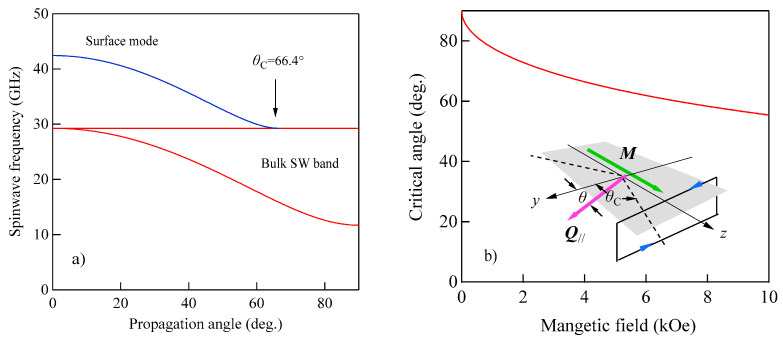
(**a**) Propagation angle development of the bulk SW band (−) and the DE mode frequency (−). We used the following set of magnetic constants: *g* = 2.09, *H* = 4.0 kOe, and 4*πM* = 21.0 kG. Here, the upper and lower bounds of the bulk SW band are displayed for clarity’s sake. (**b**) The magnetic field dependence of the critical angle *θ*_C_. The inset shows a schematic illustration of the nonreciprocal propagation characteristics of the DE mode. The critical angle is indicated by the broken lines. The allowed propagation direction of the DE mode localized on the top and bottom magnetic surfaces is indicated by the arrows on the rectangle.

The SW band became a single level at *θ* = 0 (along the *y*-axis) and gradually spread wider as *θ* approached π/2 (along the *z*-axis) as shown in Figure 4a. It is important to note that at the upper-bound SW frequency, the perpendicular component *q*_⊥_ became much larger than the in-plane component *Q*_⁄⁄_. On the contrary, we have *q*_⊥_ = 0 for the lower-bound frequency. 

There is another type of SW solution which satisfies the magnetic continuity conditions at the boundaries. This solution is the surface-localized SW and is known as the Damon–Eshbach (DE) mode. For discussions of the surface mode, it is convenient to rewrite Equations (34) and (40) as follows:

(42) $$\left(\begin{array}{c} m\left(r,t\right)\\ \varphi \left(r,t\right) \end{array}\right)=\left(\begin{array}{c} m\\ \varphi \end{array}\right)\exp\left[i\omega t+q_{⊥}x-i(Q_{//}\cos\theta )\;y+i(Q_{//}\sin\theta )\;z\right]$$

and

(43) $$q_{⊥}={\left[\frac{1+4\pi \chi_{xx}\cos^{2}\theta }{1+4\pi \chi_{xx}}\right]}^{1/2}Q_{//}=\alpha Q_{//}$$

By eliminating the potential amplitudes $\varphi_{\mp }$ outside of the magnet using the boundary conditions, we obtain a set of homogeneous equations on φ(±) to determine the surface-localized SW frequency. Per the requirement for nontrivial solutions of the homogeneous equations, we obtain:

(44) $$\begin{array}{l} -\beta {\left[\alpha \left(1+\frac{4\pi MH}{H^{2}-{\left(\omega /\gamma \right)}^{2}}\right)-1\right]}^{2}+\beta {\left[\frac{4\pi M\left(\omega /\gamma \right)}{H^{2}-{\left(\omega /\gamma \right)}^{2}}\cos\theta \right]}^{2}\\ \;\;\;\;+{\left[\alpha \left(1+\frac{4\pi MH}{H^{2}-{\left(\omega /\gamma \right)}^{2}}\right)+1\right]}^{2}-{\left[\frac{4\pi M\left(\omega /\gamma \right)}{H^{2}-{\left(\omega /\gamma \right)}^{2}}\cos\theta \right]}^{2}=0. \end{array}$$

Here, we define $\beta =\exp\left(-2Q_{//}L\right)$. To obtain the DE mode frequency, we must solve numerically Equation (44). Fortunately, we can obtain an analytical expression of the DE mode frequency in following two cases. 

Case 1: *θ* = 0 (*α* = 1)

(45) $${\left(\frac{\omega }{\gamma }\right)}^{2}={\left(H+2\pi M\right)}^{2}-{\left(2\pi M\right)}^{2}\exp\left(-2Q_{//}L\right)$$

For a film with a thickness larger than L≅λ/2, the exponential term in Equation (45) can be safely neglected. It means that a film thicker than L≅λ/2 can be treated as a semi-infinite magnet.

Case 2: *β* = 0

(46) $$\alpha \left(1+\frac{4\pi MH}{H^{2}-{\left(\omega /\gamma \right)}^{2}}\right)+1\pm \frac{4\pi M\left(\omega /\gamma \right)}{H^{2}-{\left(\omega /\gamma \right)}^{2}}\cos\theta =0$$

After some calculations, we obtain the below:

(47) $$2\pi \Delta \nu_{\mathrm{DE}}=\pm \frac{\gamma }{2}\left[\frac{H}{\cos\theta }+\left(H+4\pi M\right)\cos\theta \right]$$

Equation (47) at *θ* = 0 gives the frequency which is exactly the same as the frequencies given by Equation (45) because of *β* = 0. However, we should check whether these modes are truly eligible for the surface mode or not. From the boundary conditions, we obtain

(48) $$q_{⊥}=-Q_{//}\frac{4\pi \left(-i\chi_{xy}\right)\cos\theta +1}{1+4\pi \chi_{xx}}$$

Because the semi-infinite magnet occupies the space below x≤0, the perpendicular component $q_{⊥}$ should be positive for the eligible surface mode. By substituting the frequencies in Equation (47) into Equation (48), we find that the positive frequency in Equation (47) always gives $q_{⊥}<0$ and fails to satisfy the localization condition. We should abandon the positive frequency solution. Meanwhile, the negative frequency gives

(49) $$q_{⊥}=-Q_{//}\frac{\left(4\pi M\cos^{2}\theta +H\sin^{2}\theta \right)}{{\left[H-\left(H+4\pi M\right)\cos^{2}\theta \right]}^{2}}\left[H-\left(H+4\pi M\right)\cos^{2}\theta \right]$$

Therefore, the negative frequency mode propagating within the critical angle given by Equation (50) can be the surface-localized DE mode [24]:

(50) $$\cos\theta \geq {\left(\frac{H}{H+4\pi M}\right)}^{1/2}=\cos\theta_{C}$$

Figure 4a shows the propagation angle *θ* development of the bulk SW band given by Equation (41) and the DE mode frequency given by Equation (47). At the critical angle *θ*_C_, the DE mode frequency coincided with the upper bound of the bulk SW band. In this calculation, we used a set of the magnetic constants suitable for Fe: *g* = 2.09, *H* = 4.0 kOe, and 4*πM* = 21.0 kG. Figure 4b shows the critical angle *θ*_C_ as a function of the external magnetic field for the same set of the magnetic constants. The critical angle was gradually squeezed with the increasing magnetic field. The inset displays a schematic illustration of the nonreciprocal propagation characteristics of the DE mode. The DE mode always propagates from the left to the right across the magnetization as indicated by the arrows. At an angle *θ* beyond the critical angle *θ*_C_, the attenuation factor for the DE mode *q*_⊥_ in Equation (49) becomes negative, and the DE mode is no longer allowed above *θ*_C_. The existence of the critical angle is the reason why the DE peak appears on only one side of a SW BLS spectrum. The nonreciprocal propagation characteristics of the DE mode are schematically illustrated in Figure 4c. Note that the counterpart of the DE mode propagating along the opposite direction is located on the bottom surface of the magnet. The incident laser photon can never interact with the counterpart DE mode because of the absorption effect of visible light, as we have already mentioned. The DE mode frequency was always above the bulk SW frequency band. At the critical angle, the DE mode frequency was just on the upper bound of the bulk SW band (see Equation (41)). Sandercock and Wettling nicely presented how the DE mode behaves as the propagation angle approaches the critical angle [13]. Their results clearly show that the DE mode decays into the bulk SW band, and no surface mode is allowed beyond the critical angle.

**Figure 5:**
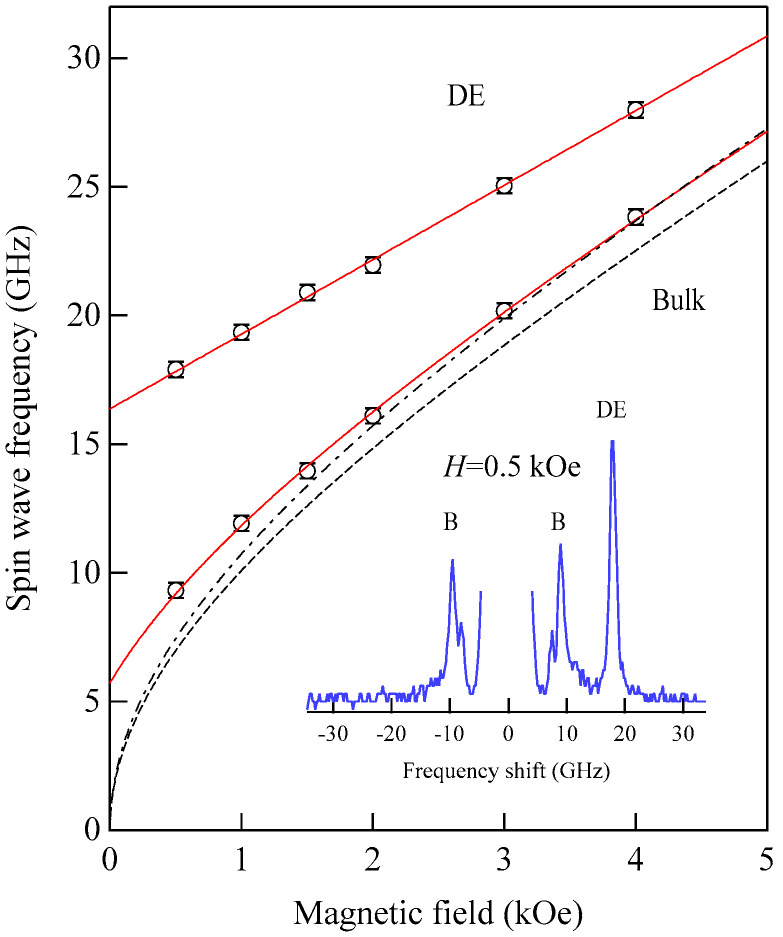
SW frequencies of Fe_64_Al_19_O_17_ film as a function of the magnetic field. A BLS spectrum at *H* = 0.5 kOe is also shown. The solid lines are the calculated SW frequencies using Equation (33) with *g* = 2.07, 4*πM*_B_ = 11.1 kG, 4*πM*_DE_ = 11.3 kG, and *H*_ex_ = 0.34 kOe. The broken line (4*πM*_B_ = 11.1 kG) and dots and dashes (4*πM*_B_ = 12.7 kG) are the calculated frequencies without the *H*_ex_ term [54].

Figure 5 shows the SW frequencies as a function of the magnetic field for Fe_64_Al_19_O_17_ nanogranular film [54]. The inset shows a BLS spectrum observed at *H* = 0.5 kOe. Because we have not performed the polarization selection for the scattered beam, SAW peaks appear as a pair of small peaks just below the B-peaks. I will soon explain the solid lines, the broken line, and the dots and dashes.

In these measurements, both the bulk and DE modes were propagating perpendicular to the magnetization, and their frequencies are given by

(51) $$\Delta \nu_{B}=\frac{\gamma }{2\pi }H^{1/2}{\left(H+4\pi M\right)}^{1/2}$$

and

(52) $$\Delta \nu_{\mathrm{DE}}=\frac{\gamma }{2\pi }\left(H+2\pi M\right)$$

Because the frequency shifts $\Delta \nu_{B}$ and $\Delta \nu_{\mathrm{DE}}$ were directly obtained from the BLS spectrum, both frequencies should be reproduced by the same magnetic constants as a function of the magnetic field. However, sometimes we encountered somewhat different 4*πM* values for the bulk and DE modes. The broken line and dots and dashes in Figure 5 are the calculated bulk SW frequencies using Equation (51) by changing the 4πM value. It is clear that Equation (51) fails to reproduce the observed magnetic field dependence of the bulk SW frequency. Because our model is oversimplified in the first attempt, we will try to include the exchange coupling term into Equation (51). For long wavelength SWs, the exchange coupling can be represented by the differential operator $H_{\mathrm{ex}}=-D\nabla^{2}$. Because the external magnetic field and the magnetization are collinear, we can replace the external magnetic field *H* with *H* + *DQ*^2^. Here, *Q* is the wave vector of the SW, and *D* is the SW stiffness constant and related to the exchange stiffness constant *A* through the relation of *D* = 2*A*/*M*. With the exchange field term, Equation (51) is replaced by

(53) $$\Delta \nu_{B}=\frac{\gamma }{2\pi }{\left(H+DQ^{2}\right)}^{1/2}{\left(H+DQ^{2}+4\pi M\right)}^{1/2}$$

The solid lines in Figure 5 are calculated bulk SW frequencies with the exchange field term (*DQ*^2^ = 0.34 kOe) in Equation (53). Although the exchange field value is usually much smaller than the other fitting parameters, typically less than 0.5 kOe, agreements between the observed and calculated bulk SW frequencies are excellent. In spite of the importance of the exchange field term for qualitative fitting of the bulk SW frequencies, as shown in Figure 5, we cannot determine the *D* constant from the fitting because we have no information on the SW wave vector *Q* in Equation (53) due to the relaxation of the momentum conservation law. The DE mode frequency is rather insensitive to the exchange term because of the linear dependence of the frequency on the external magnetic field. Furthermore, the existence of the DE mode was derived from the boundary conditions, and the negligibly small $DQ_{//}^{2}$ term was completely masked by the other quantities in Equation (52). 

Up to this stage, we considered soft ferromagnetic materials with negligibly small MAE. For such small-MAE cases, it may be an easy- or hard- axis type, and we can readily align the magnetization along the external magnetic field. Let us consider the uniaxial in-plane MAE given by

(54) $$E_{K}=-K_{//}{\left(\frac{M_{z}}{M}\right)}^{2}$$

Here, *K*_//_ is the in-plane MAE constant. When we apply the magnetic field along the easy direction and examine SWs propagating perpendicular to the magnetization (*θ* = 0), the SW frequencies are given by

(55) $$\Delta \nu_{B}=\frac{\gamma }{2\pi }{\left(H+H_{K//}+DQ^{2}\right)}^{1/2}{\left(H+H_{K//}+DQ^{2}+4\pi M\right)}^{1/2}$$

and

(56) $$\Delta \nu_{\mathrm{DE}}=\frac{\gamma }{2\pi }\left(H+H_{K//}+2\pi M\right)$$

Here, we defined the in-plane anisotropy field $H_{K//}=2K_{//}/M$. Meanwhile, for the magnetic field along the hard direction, we have

(57) $$\Delta \nu_{B}=\frac{\gamma }{2\pi }{\left[\left(H+DQ^{2}\right)\left(H-H_{K//}+DQ^{2}+4\pi M\right)-4\pi MH_{K//}{\left(\frac{Q_{⊥}}{Q}\right)}^{2}\right]}^{1/2}$$

and

(58) $$\Delta \nu_{\mathrm{DE}}=\frac{\gamma }{2\pi }\left(H+2\pi M-\frac{H_{K//}}{2}\right)$$

The bulk SW in an isotropic magnet forms the bulk SW band given by Equation (41). The bandwidth depends on the in-plane propagation direction *θ*. On the other hand, when the external magnetic field is applied along the hard direction, the MAE introduces the SW band for the bulk SW propagating even for the *θ* = 0 direction. The bandwidth given by 4*πMH*_K//_ depends on both the strength of the MAE and the perpendicular component of the SW wave vector. The main contribution for the SW BLS is from *Q*_⊥_/*Q* < 0.5, and the bandwidth 4*πMH*_K//_ is usually smaller than the bulk SW’s peak width. Hence, it is impractically difficult to determine the MAE parameters from BLS measurement by itself.

Next, we consider the out-of-plane type MAE given by

(59) $$E_{K}=-K_{⊥}{\left(\frac{M_{x}}{M}\right)}^{2}$$

We can also define the out-of-plane anisotropy field $H_{K⊥}=2K_{⊥}/M$. For a weak anisotropy field, which satisfies the in-plane magnetization condition given by $4\pi M-H_{K⊥}\geq 0$, the magnetization is confined within the film plane and aligned colinear to the external magnetic field. In this case, the upper and lower bounds of the SW band are given by

(60) $$\begin{array}{l} {\left(\frac{\omega }{\gamma }\right)}^{2}=\left(H+DQ^{2}\right)\left(H+DQ^{2}+4\pi M-H_{K⊥}\right)-4\pi MH_{K⊥}{\left(\frac{Q_{//}}{Q}\right)}^{2}\\ \;\;=\{\begin{array}{c} \left(H+DQ^{2}\right)\left(H+DQ^{2}+4\pi M-H_{K⊥}\right)\left(Q_{//}/Q=0\right)\\ \left(H+DQ^{2}-H_{K⊥}\right)\left(H+DQ^{2}+4\pi M\right)\left(Q_{//}/Q=1\right) \end{array} \end{array}$$

and the DE mode frequency is given by

(61) $$\frac{\omega }{\gamma }=H+2\pi M-\frac{H_{K⊥}}{2}$$

In this case, the main contribution for the bandwidth is from *Q*_⊥_/*Q* > 0.5. For weak magnetic fields which satisfy $H+DQ^{2}-H_{K⊥}\leq 0$, the lower bound of the SW band should be set to zero. On the contrary, the out-of-plane MAE is large enough to overcome the in-plane magnetization condition, and the perpendicular magnetization state is the ground state under zero magnetic field. We will discuss this case later. 

### 5.2. Thin Films

As already mentioned, a magnetic film thicker than ~*λ*/2 can be treated as a semi-infinite magnet. Now, what happens for thinner films? For thinner films with thicknesses less than ~1000 Å, new aspects of SWs, known as the standing SWs (SSWs), appear in a BLS spectrum. The first BLS observation of the SSWs was reported by Grimsditch and Malozemoff on metallic amorphous Fe_80_B_20_ films. They determined the SW stiffness constant of *D*_BLS_ = (1.4 ± 0.2) × 10^−9^ Oe·cm^2^ [57]. Successively, BLS from the SSWs has been reported on various ferromagnetic thin films [11]. Neutron scattering is the best technique to investigate SW dynamics in the entire Brillouin zone and can be used to determine the SW stiffness constant *D*_NS_. However, neutron scattering requires a reactor and eventually becomes a huge project. In this section, in order to distinguish the SW stiffness constant obtained from BLS and neutron scattering, we add the subscript BLS and NS to the *D* constant. Otherwise, we simply use the symbol *D* for the BLS SW stiffness constant. By observing the SSW structure, we can precisely determine the SW stiffness constant *D*_BLS_ even in a small optical laboratory. This is one of the virtues of the SW BLS technique. The research group in Brookhaven National Laboratory (NBL) extensively investigated SWs in Fe, Ni, and Co in the 1960s [58]. Note that the BLS technique gives information near the Brillouin zone center thanks to visible laser light as an excitation source. We can change the *D*_BLS_ value in the 10^−9^ Oe·cm^2^ unit to the *D*_NS_ value in the meV·Å^2^ unit used in neutron scattering and magnetization studies by a formula of *D*_BLS_ = 1.728 × 10^−2^*D*_NS_/*g*. Neutron scattering and BLS give *D*_NS_~280 meV·Å^2^ for Fe, and these results are in good agreement [58,59].

**Figure 6:**
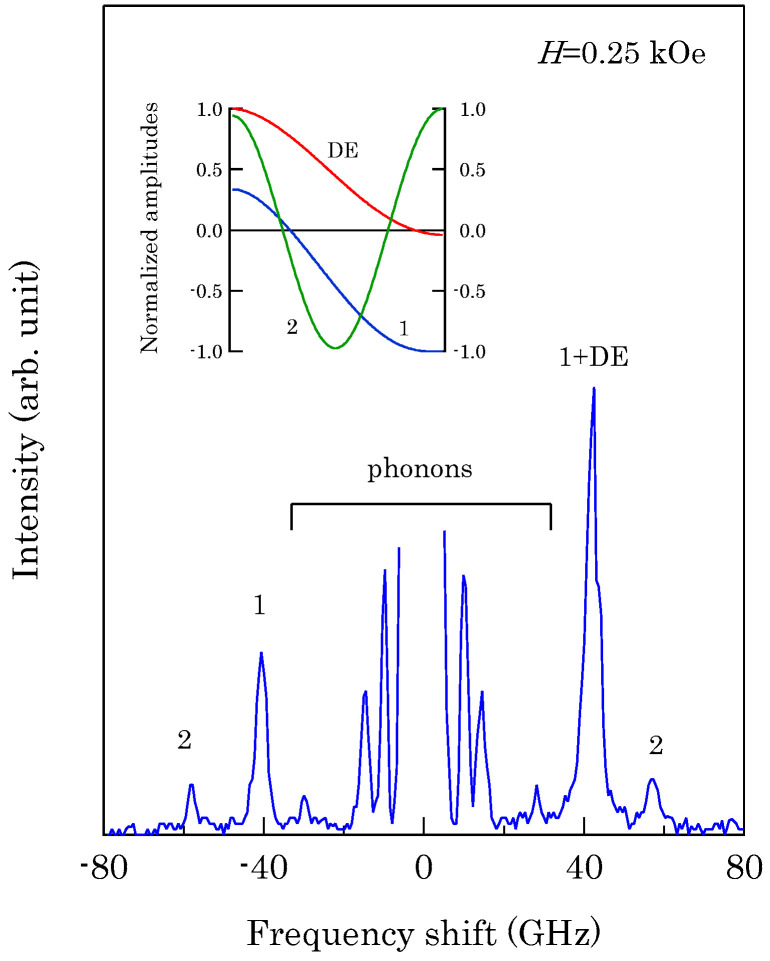
BLS spectrum observed from an epitaxial $\left(10\bar{1}0\right)$ Co thin film 45 nm thick at room temperature. Because no polarization selection has been done, scattering from SWs and SAWs was observed. The structure labeled “phonons” within ±30 GHz around the elastic RS peak was due to SAWs. These were assigned to the Rayleigh wave and the first and second order Sezawa waves with increasing frequency. SW peaks up to the second order SSW appeared above ±40 GHz. Note that the phonon peak intensities are symmetrical for the Stokes and anti-Stokes peaks. On the other hand, the SW peaks were asymmetric. The insert shows calculated *n* = 1 (−) and 2 (−) SSW and DE mode (−) profiles [60]. For clarity’s sake, I display the profiles of the DE mode localized on the top surface and the SSW modes for the bottom surface.

Figure 6 shows an example of SSW spectrum observed from a 450 ± 10 Å thick epitaxial $\left(10\bar{1}0\right)$ Co film deposited on a 500 Å thick Cr (211) buffer layer prepared on MgO (110) substrate at IMRAM, Tohoku University [60,61]. 

This spectrum was excited by the *p*-polarized 5320 Å line from a DPSS laser. Because we have not performed the polarization selection for the scattered light in this measurement, the SAW structures, indicated by phonons, were also observed. The peaks indicated by labels 1 and 2 are the first and second SSW peaks. The peak indicated by 1 + DE on the anti-Stokes side consists of the first SSW and the DE peaks. Note that the DE mode in thinner films also retained the nonreciprocal propagating character. When we define the critical angle $\theta_{C}$, at which the DE mode frequency given by Equation (45) is equal to the upper bulk band frequency, we obtain Equation (50) again by using Equation (44). For brevity’s sake we ignore the exchange term for the upper bulk band. For thinner magnetic films, the perpendicular component $q_{⊥}$ of the SW wave vector was quantized into $q_{⊥}\left(n\right)=n\pi /L$ (*n* = 1, 2, …). In this case, the perpendicular components $q_{⊥}\left(n\right)$ were well-defined, and the momentum conservation law during the scattering process recovered. This is the reason why we can observe sharp SSW peaks in our spectrum. On our BLS results from this epitaxial $\left(10\bar{1}0\right)$ Co film, we will discuss details later. 

**Figure 7:**
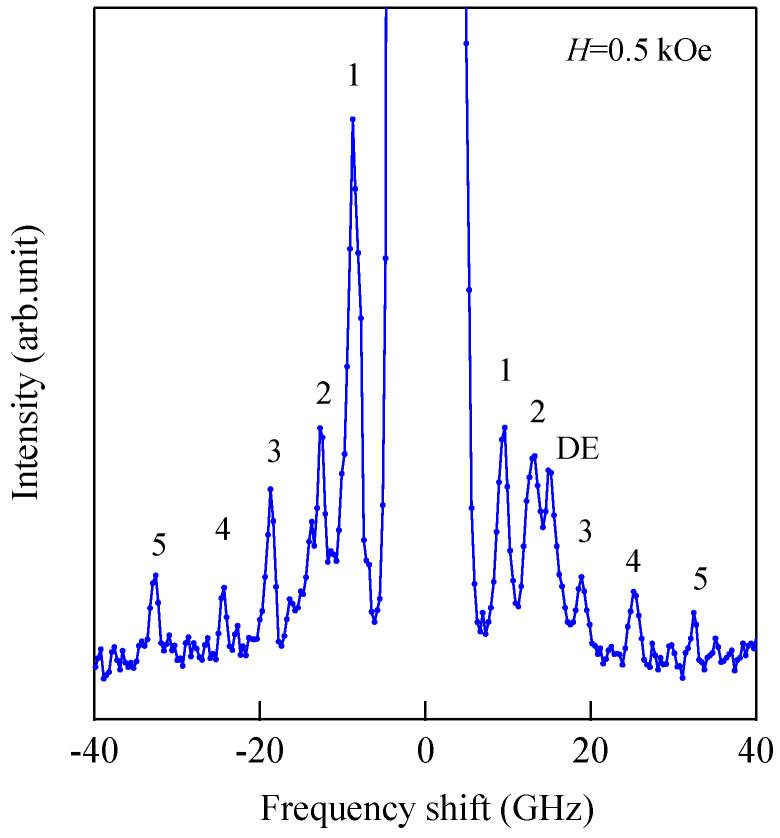
BLS spectrum observed from a 1000 ± 50 Å thick Co_85_Nb_12_Zr_3_ film prepared by means of rf-magnetron sputtering from alloy target onto glass substrate. Labels 1–5: SSW mode number; DE: DE surface mode. (Copyright (1994) The Japan Society of Applied Physics) [62].

Figure 7 shows another example of SSWs observed from 1000 ± 50 Å-thick sputtered Co_85_Nb_12_Zr_3_ film on a glass substrate prepared at RISM, Tohoku University [62]. 

**Figure 8:**
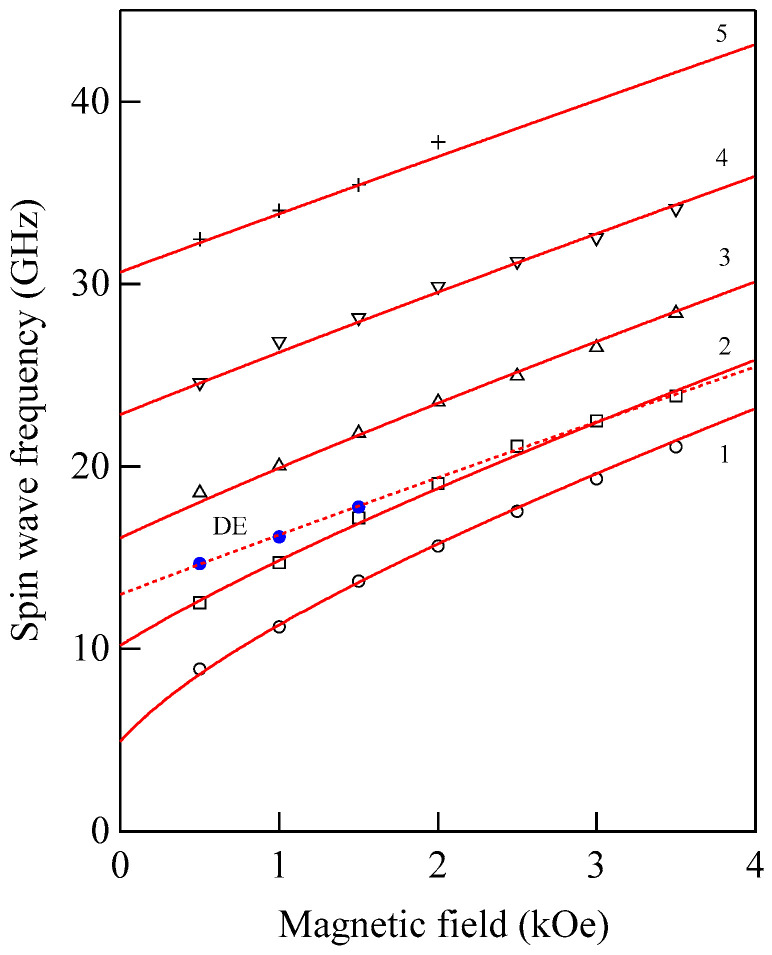
Magnetic field dependence of the SSW and DE mode frequencies. The open symbols and + symbol are for the SSW modes, and (●) is for the DE mode. Here, the labels 1 to 5 stand for the SSW mode number. The solid and broken lines were calculated from Equations (59) and (60) with the magnetic constants given in the text (Copyright (1994) The Japan Society of Applied Physics) [62].

We set ϑ = 15° and *H* = 0.5 kOe. We could observe the SSW peaks up to the fifth order in this spectrum. Note that the peak intensities are highly asymmetric between the Stokes and anti-Stokes sides. This is a characteristic feature of SW BLS, as I have already mentioned, as the interference effects it. The DE peak appears only on the anti-Stokes side. The DE peak intensity is not high compared to the SSW peaks because of the small incident angle. In fact, when we increase the incident angle *ϑ*, the DE peak intensity gradually increases. Figure 8 shows the SW frequencies as a function of the magnetic field.

The open symbols stand for the SSWs, and the filled circles stand for the DE mode. Above *H* = 1.0 kOe, we could not fully resolve the DE mode from the second-lowest-order SSW peak. In order to determine the magnetic constants of the Co_85_Nb_12_Zr_3_ film while taking into account the quantization effect on the $q_{⊥}$ components, we employed a conventional formula given below:

(62) $$\Delta \nu \left(n\right)=\frac{\gamma }{2\pi }{\left[H+D\left\{Q_{//}^{2}+{\left(\frac{n\pi }{L}\right)}^{2}\right\}\right]}^{1/2}{\left[H+D\left\{Q_{//}^{2}+{\left(\frac{n\pi }{L}\right)}^{2}\right\}+4\pi M\right]}^{1/2}\left(n=1,2,\ldots \right)$$

and

(63) $$\Delta \nu_{DE}=\frac{\gamma }{2\pi }\left[{\left(H+2\pi M\right)}^{2}-{\left(2\pi M\right)}^{2}\exp\left(-2Q_{//}L\right)\right]$$

It can be readily recognized from Equation (62) that the *D* constant governs the splitting between the SSW frequencies. The solid lines in Figure 8 are the calculated SSW frequencies from Equation (62), and the broken line is the calculated DE mode frequency from Equation (63) with the magnetic constants listed in Table 1.

We obtained $4\pi M_{\mathrm{VSM}}=10.1\pm 0.2\;\left(\mathrm{kG}\right)$ from a VSM measurement and evaluated the exchange stiffness constant *A* = 0.98 ± 0.14 (×10^−6^ erg/cm) using the magnetic constants. With these constants, an excellent agreement between the calculation and observation was obtained. This agreement is not only for this case. Usually, Equations (62) and (63) gave good agreement between calculations and observations. 

**Figure 9:**
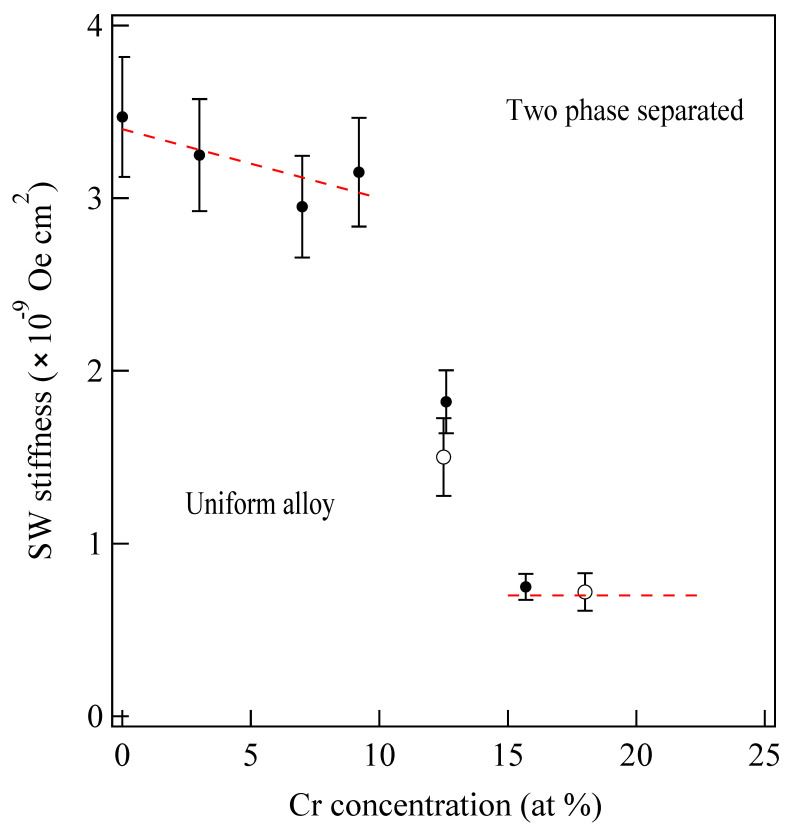
A summary of the SW stiffness constant *D* of the Co_1−x_Cr_x_ binary system (*x* in at % unit) as a function of the Cr content *x*. We added the *D*_BLS_ values for *x* = 12.5 from [65] and *x* = 18 from [66]. The colored area shows the transition region between the uniform alloy state and the two-phase separated state. The broken lines give *D* (*x*) = (3.41 − 0.043*x*) × 10^−9^ Oe·cm^2^ and 0.7 × 10^−9^ Oe·cm^2^, respectively [63,64].

Figure 9 shows the SW stiffness constant *D* in Co_100−x_Cr _x_ binary alloy of 300~500 Å in thickness as a function of the Cr at % [63,64]. 

These alloys were prepared at RISM, Tohoku University. For the CoCr binary alloy system, phase separation occurred from the Co-rich uniform state below *x*~10 at % to the phase-separated state. In the phase-separated state, the Co-rich ferromagnetic regions were surrounded by the nonmagnetic Cr-rich grain boundaries above *x*~12 at %. Because the exchange coupling is purely a quantum mechanical effect due to the electron itineracy, overlapping of the electron wave functions, or both, we can expect that the exchange coupling strength is very sensitive to the microscopic atomic structure inside a film. Figure 9 clearly shows that a drastic change of the exchange coupling scheme from the direct coupling in the Co-rich uniform state to a weak indirect coupling via Cr-rich regions took place around *x* = 10~15 at %. In this way, the BLS technique can provide quantitative information on these magnetic interactions.

Because we have phenomenologically introduced Equation (62), we must take into account the exchange coupling and the MAE and derive more rigorous descriptions of the SW frequencies. We applied the dipole-exchange model with continuum approximation to discuss the SWs. The exchange coupling can be calculated with the second derivative operator given by $H_{\mathrm{ex}}=-D\nabla^{2}$ in this model. Recent developments in film preparation techniques allowed us to examine various types of epitaxial structures. For materials with hexagonal or tetragonal structures, the MAE played more important roles than for the cubic structures. Thinking of the epitaxial Co $\left(10\bar{1}0\right)$ films, we considered the uniaxial in-plane MAE up to the fourth order as below [60]:

(64) $$E_{K}=K_{1}\sin^{2}\phi +K_{2}\sin^{4}\phi$$

**Figure 10:**
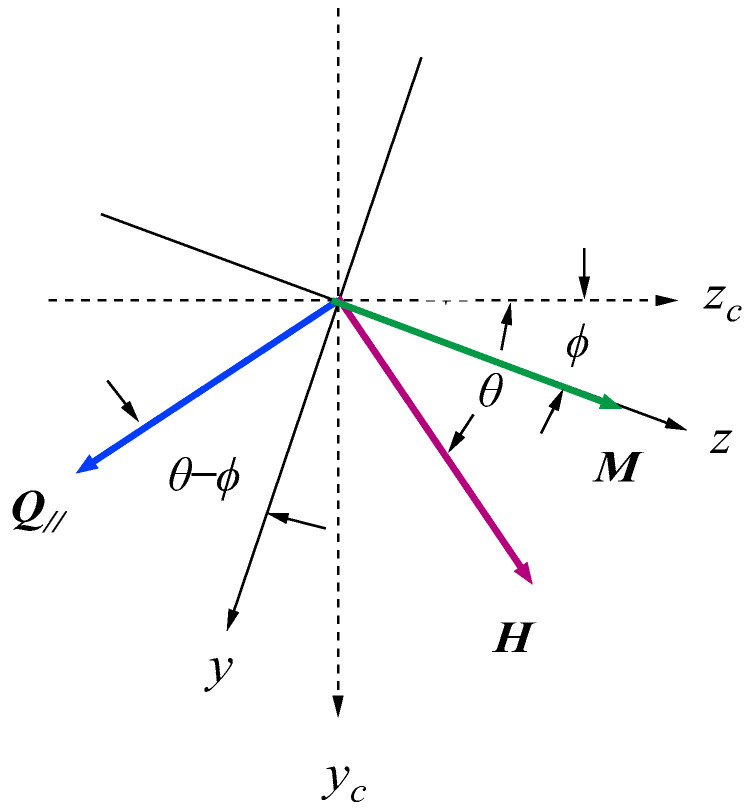
Schematic illustration on the magnetization rotation by the external magnetic field applied in the *y*–*z* plane. The magnetic field was applied along the *θ* direction measured from the crystal easy axis *z*_C_. The rotation angle of the magnetization measured from the easy axis *ϕ* can be determined by Equation (65). BLS observed the SWs propagating along the *y* direction, which is always perpendicular to the external magnetic field.

Figure 10 shows the coordinate systems and scattering geometry used in our discussions. The crystallographic coordinates are shown by $\left(x_{c},y_{c},z_{c}\right)$. 

Here, the *x*_c_ axis is along the surface normal direction, and the film surfaces are located at *x* = ±*L*/2. The easy axis is along the *z*_c_ direction. We applied a magnetic field *H* to make an angle *θ* between the magnetic field *H* and the *z*_c_ axis, and we always measured SWs propagating perpendicular to *H*, as shown in Figure 10. Then, the magnetization *M* rotates from the easy axis. However, *M* will be not collinear with *H* because of the MAE. For convenience’s sake, we introduced the magnetization coordinates (*x*,*y*,*z*) by rotating the crystallographic coordinates around the *x*_c_ axis by an angle *ϕ*. The *z* direction is along the magnetization direction. The rotation angle *ϕ* can be determined by the competition between the external magnetic field and the MAE as given by

(65) $$H\sin\left(\theta -\phi \right)=\sin2\phi \left(\frac{K_{1}}{M}+\frac{2K_{2}}{M}\sin^{2}\phi \right)$$

We introduced the SW variable *m* as the small amplitude precession motion around the static magnetization *M*. The LL equation of motion on *M*(*t*) = *M* + *m*(*t*) is already given in Equation (30). The effective magnetic field *H*_eff_ consists of the external magnetic field *H*, the uniaxial magnetic anisotropy field $H_{K}=-\nabla_{M}E_{K}$, the exchange field $H_{ex}=\left(D/M\right)\nabla^{2}M$, and the demagnetization field ***h*** as follows:

(66) $$H_{eff}=H+H_{K}+H_{ex}+h$$

When *H* and *M* are not collinear with each other, we must include the longitudinal components *m*_z_ and *h*_z_ in addition to the their transverse components. Because the MAE in Equation (39) is defined in the crystallographic coordinates, we must rewrite it in the magnetization coordinate variables in order to evaluate the anisotropy field. It is given by

(67) $$\begin{array}{l} E_{K}≅\sin2\phi \left(K_{1}+2K_{2}\sin^{2}\phi \right)\left(\frac{m_{y}}{M}\right)\\ \;\;+\left\{K_{1}\cos2\phi +2K_{2}\left(\sin^{2}2\phi -\sin^{2}\phi \right)\right\}{\left(\frac{m_{y}}{M}\right)}^{2}\\ \;\;+\cos^{2}\phi \left(K_{1}+2K_{2}\sin^{2}\phi \right){\left(\frac{m_{x}}{M}\right)}^{2}. \end{array}$$

The linearized LL equation into a compact form given by

(68) $$\frac{1}{\gamma }\frac{d}{dt}m_{x}=\left(H_{1}-D\nabla^{2}\right)m_{y}-Mh_{y},$$

(69) $$\frac{1}{\gamma }\frac{d}{dt}m_{y}=-\left(H_{2}-D\nabla^{2}\right)m_{x}+Mh_{x},$$

and

(70) $$\frac{1}{\gamma }\frac{d}{dt}m_{z}=\left(H\sin\theta \cos\phi -H_{a}\sin\phi \right)m_{x}=0$$

Here, we define *H*_a_, *H*_1_, and *H*_2_ as follows:

(71) $$H_{a}=H\cos\theta +\left(\frac{2K_{1}}{M}+\frac{4K_{2}}{M}\sin^{2}\phi \right)\cos\phi ,$$

(72) $$H_{1}=H\cos\left(\theta -\phi \right)+\frac{2K_{1}}{M}\cos2\phi +\frac{4K_{2}}{M}\left(\sin^{2}2\phi -\sin^{2}\phi \right),$$

and

(73) $$H_{2}=H\cos\left(\theta -\phi \right)+\frac{2K_{1}}{M}\cos^{2}\phi +\frac{K_{2}}{M}\sin^{2}2\phi .$$

Equation (70) confirms that the longitudinal component *m*_z_ does not contribute to the SWs. We assume plane-wave-type space-time dependence for the dynamical variables:

(74) $$\left(\begin{array}{c} m\left(r,t\right)\\ h_{d}\left(r,t\right) \end{array}\right)=\left(\begin{array}{c} m\\ h_{d} \end{array}\right)\exp\left[i\omega t-iq_{⊥}x-iQ_{//}\cos\left(\theta -\phi \right)y+iQ_{//}\sin\left(\theta -\phi \right)z\right]$$

The first equation of Equation (32) gives only two independent equations—for example,

(75) $${\left(\nabla \times h\right)}_{y}=Q_{//}\sin\left(\theta -\phi \right)h_{x}+q_{⊥}h_{z}=0$$

and

(76) $${\left(\nabla \times h_{d}\right)}_{z}=q_{⊥}h_{dy}-Q_{//}\cos\left(\theta -\phi \right)h_{dx}=0$$

Combining these equations, we obtain the ${\left(\nabla \times h\right)}_{x}$ component. The second equation of Equation (32) can be regarded as an additional equation of motion to the LL equation.

(77) $$q_{⊥}\left(h_{x}+4\pi m_{x}\right)+Q_{//}\cos\left(\theta -\phi \right)\left(h_{y}+4\pi m_{y}\right)-Q_{//}\sin\left(\theta -\phi \right)h_{z}=0$$

A set of five homogeneous equations for the five variables $\left(m_{x},m_{y},h_{x},h_{y},h_{z}\right)$ gives an equation for the nontrivial solutions:

(78) $$\begin{array}{l} {\left(P^{2}\right)}^{3}+\frac{\left(H_{1}+H_{2}+4\pi M\right)}{DQ_{//}^{2}}{\left(P^{2}\right)}^{2}\\ \;\;\;+\frac{\left\{H_{1}\left(H_{2}+4\pi M\right)-4\pi MDQ_{//}^{2}\sin^{2}\left(\theta -\phi \right)-{\left(\omega /\gamma \right)}^{2}\right\}}{{\left(DQ_{//}^{2}\right)}^{2}}\left(P^{2}\right)\\ \;\;\;\;\;+\frac{4\pi M\left\{H_{2}\cos^{2}\left(\theta -\phi \right)-H_{1}\right\}}{{\left(DQ_{//}^{2}\right)}^{2}}=0. \end{array}$$

Here, we define $P^{2}=\left(q_{⊥}^{2}+Q_{//}^{2}\right)/Q_{//}^{2}$ and adapt the partial wave technique in which SWs are constructed by a sum of bulk partial waves. By adjusting coefficients for the partial waves, we made the constructed SWs satisfy the proper boundary conditions. Equations (75) and (76) give six *q*_⊥_ solutions allowed for the bulk partial waves [34]. 

Combining the linearized LL equation with the effective magnetic field given by Equation (66) and Maxwell’s equations, as seen in Equation (32), we obtain in terms of the partial waves

(79) $$m_{x}=\sum_{j=1}^{6}M\frac{\left(H_{1}+DQ_{j}^{2}\right)h_{jx}-i\tilde{\omega }h_{jy}}{\left(H_{1}+DQ_{j}^{2}\right)\left(H_{2}+DQ_{j}^{2}\right)-{\tilde{\omega }}^{2}}=\sum_{j=1}^{6}M\frac{\left(H_{1}+DQ_{j}^{2}\right)-i\tilde{\omega }\frac{Q_{//}}{q_{⊥j}}\cos\left(\theta -\phi \right)}{\left(H_{1}+DQ_{j}^{2}\right)\left(H_{2}+DQ_{j}^{2}\right)-{\tilde{\omega }}^{2}}h_{jx}$$

and

(80) $$m_{y}=\sum_{j=1}^{6}M\frac{i\tilde{\omega }h_{jx}+\left(H_{2}+DQ_{j}^{2}\right)h_{jy}}{\left(H_{1}+DQ_{j}^{2}\right)\left(H_{2}+DQ_{j}^{2}\right)-{\tilde{\omega }}^{2}}=\sum_{j=1}^{6}M\frac{i\tilde{\omega }+\left(H_{2}+DQ_{j}^{2}\right)\frac{Q_{//}}{q_{⊥j}}\cos\left(\theta -\phi \right)}{\left(H_{1}+DQ_{j}^{2}\right)\left(H_{2}+DQ_{j}^{2}\right)-{\tilde{\omega }}^{2}}h_{jx}$$

Here, we introduced $\tilde{\omega }=\omega /\gamma$ for convenience and used the demagnetization fields instead of the magnetic potential φ. By adapting the magnetic boundary conditions at x=±L/2, we can eliminate the demagnetization fields outside of the magnet and obtain a set of two equations given by

(81) $$\sum_{j=1}^{6}\left[1+4\pi M\frac{\left(H_{1}+DQ_{j}^{2}\right)-i\tilde{\omega }\frac{Q_{//}}{q_{⊥j}}\cos\left(\theta -\phi \right)}{\left(H_{1}+DQ_{j}^{2}\right)\left(H_{2}+DQ_{j}^{2}\right)-{\tilde{\omega }}^{2}}\pm i\frac{Q_{//}}{q_{⊥j}}\right]\exp\left(\mp iq_{⊥j}L/2\right)\;h_{xj}=0$$

The upper compound symbols in Equation (81) are for the top surface at x=L/2 and the lower symbols for the bottom surface at x=−L/2, respectively. Because we have six unknown variables *h_xj_*, we need four more boundary conditions. Rado and Weertman derived generalized boundary conditions, which is known as the Rado–Weertman surface pinning conditions [26], based on the LL equation. It is given by

(82) $$M\times {\left(\frac{D}{M}\frac{\partial }{\partial n}m-\nabla_{M}E_{surf}\right)|}_{x=\pm L/2}=0$$

where $E_{surf}$ is the surface magnetic anisotropy (SMA) energy and we assume a SMA energy with the in-plane and out-of-plane terms as given by

(83) $$E_{\mathrm{surf}}=-k_{⊥}\;{\left(\frac{m_{x}}{M}\right)}^{2}-k_{//}{\left(\frac{m_{y}}{M}\right)}^{2}$$

Now, we have a set of six homogeneous equations for six unknown variables. The SW frequencies can be obtained by solving numerically a 6 × 6 boundary condition determinant (BCD) equation. When we have no SMEs (no surface pinning) and the limiting case of $Q_{//}\approx 0$, we can obtain asymptotic expression for the SW frequencies given by

(84) $$\Delta \nu \left(n\right)=\frac{\gamma }{2\pi }{\left[H+D{\left(\frac{n\pi }{L}\right)}^{2}\right]}^{1/2}{\left[H+D{\left(\frac{n\pi }{L}\right)}^{2}+4\pi M\right]}^{1/2}\;\;\;\left(n=1,2,\ldots \right)$$

and

(85) $$\Delta \nu_{DE}=\frac{\gamma }{2\pi }\left[{\left(H+2\pi M\right)}^{2}-{\left(2\pi M\right)}^{2}\exp\left(-2Q_{//}L\right)\right]$$

These are exactly the same as Equations (62) and (63). As already we have seen, these expressions can describe well the magnetic field dependence of the SW frequencies. 

**Figure 11:**
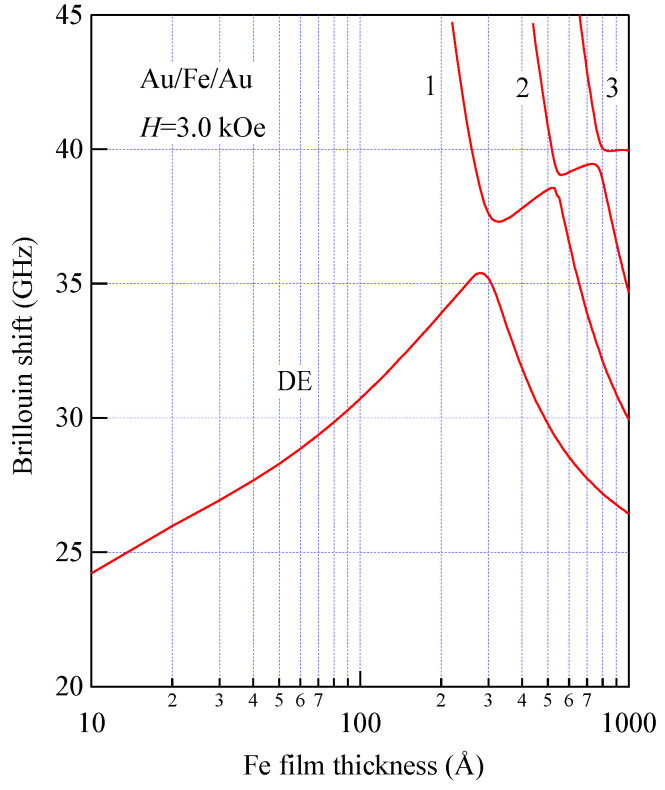
Calculated SW frequencies (−) propagating along the [110] direction on the epitaxial (001) Fe film as a function of the film thickness. The magnetic field was applied along the [1–10] direction, and the cubic MAE and the effective demagnetization factor *D*_⊥_ were included in this calculation. The broken lines are just for eye.

Figure 11 shows an example of the SW frequencies of bcc Fe propagating with *Q*_//_ = 1.85 × 10^5^ cm^−1^ along the [110] direction on the (001) surface at *H* = 3.0 kOe as a function of film thickness down to 10 Å. 

The SW frequencies were obtained by solving the 6 × 6 BCD equation. We used a set of the magnetic parameters listed Table 1 in this calculation. It is known that the demagnetization factor 4*π* for thick films should be replaced by the effective demagnetization factor 4*πf*_⊥_. The *f*_⊥_ factor was given by 1−0.4245/*n* for the bcc (001) structure and 1−0.2338/*n* for the fcc (001) structure [67]. Here, *n* is the number of the atomic layers stacked on the film. We have included the 4*πD*_⊥_ term in our calculations. In Figure 11, the label DE stands for the DE mode frequency, and the labels 1, 2, and 3 stand for the first, second, and third SSW frequencies. For Fe films with thicknesses less than 100 Å, only the DE mode appears in a BLS spectrum, and the SSW peaks appear well above 50 GHz. Another interesting observation is the anticrossing effect between the DE mode and each SSW mode. Therefore, it is clear that Equations (84) and (85) give a good description of the DE and SSW frequencies when these frequencies are well separated. For thinner films with thicknesses less than ~300 Å, the DE mode localized on the opposite surface may appear in a spectrum. The amplitude of the opposite DE mode was roughly estimated to be exp(−*Q*_//_*L*)~0.57 on the laser-illuminated surface. Of course, the spectrum exhibited quite asymmetric peak intensities. 

Various structures of thin Co films, including polycrystalline films [63,68], bcc films [69,70,71], and fcc, bcc, and hcp films [72], have been extensively investigated by BLS since the early stage of the BLS SW studies. One of the most pronounced magnetic properties of Co in the hcp structure is the large uniaxial MAE, which is about one order of magnitude larger than the MAE of cubic Fe and Ni. The MAE for hcp Co is given by Equation (64). The *K*_1_ and *K*_2_ constants take positive values of ~10^6^ erg/cm^3^ at room temperature. Because of the large MAE, hcp Co-based alloys have been widely applied for many industrial applications. As I have already mentioned, it is possible to grow epitaxial $\left(10\bar{1}0\right)$ Co films which possess both the easy direction (hcp [001] axis) and the hard direction (hcp [010] axis) within a film plane. A BLS analysis from the epitaxial $\left(10\bar{1}0\right)$ Co thin films was performed by Grimsditch, Fullerton, and Stamps [73]. The SW stiffness constant of hcp Co has been a subject of controversy. The *D*_NS_ values of 490–510 meV·Å^2^ for hcp Co crystals by the BNL group [58] were obviously larger than the recent BLS values of *D*_BLS_ = 430–470 meV·Å^2^ [63,68,71,73]. 

**Figure 12:**
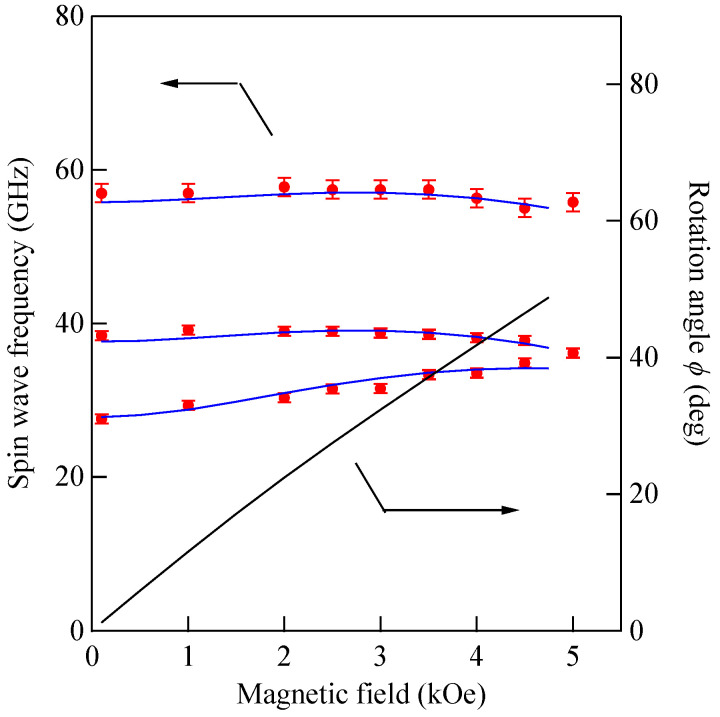
Magnetic field dependence of the SW frequencies (●) in the scattering geometry (B). The solid lines were calculated for the SW frequencies (−) and the rotation angle *ϕ* of the magnetization (−) using the full magnetic constants given in the text [74].

We performed BLS measurements on the epitaxial hcp $\left(10\bar{1}0\right)$ Co thin films [73,74]. When we adapt the scattering geometry (A) in which the magnetic fields were applied along the easy direction, only the *K*_1_ term in Equation (61) contributed to the SW frequencies because *θ* = *ϕ* = 0° in Equations (72) and (73). An example of a BLS spectrum observed from the scattering geometry (A) is shown in Figure 6. The first and second order SSWs and the DE mode can be seen. The inset shows the mode profiles of these SSWs. In order to determine the *K*_2_ constant, we must adapt the scattering geometries (B) and (C). The magnetic field dependence of the SW frequencies obtained from the scattering geometry (B) is shown in Figure 12. 

In this case, the magnetic field was always applied along the *y*_c_-direction in Figure 10. The calculated SW frequencies and the rotation angle are shown by the solid lines. We summarized the parameters used in our calculations in Table 1. These MAE constants, *K*_1_ and *K*_2_, and the SW stiffness constant are in good agreement with the ones reported by Grimsditch et al. [73], The present SW stiffness constant of *D*_BLS_ = 3.39 × 10^−9^ Oe·cm^2^ is equivalent to *D*_BLS_ = 427 meV·Å^2^ and in good agreement with the previous values. There are independent NS results on hcp Co reported by using the Kraków neutron spectrometer [75]. Both NS groups employed a two-parameter model for SW dispersion given by *ε*_Q_ = *D*_NS_*Q*^2^(1 − *βQ*^2^). The Kraków group obtained *D*_NS_ = 437 ± 20 meV·Å^2^ and *β* = 0.345 Å^2^, whereas the BNL group obtained a set of *D*_NS_ = 510 meV·Å^2^, *β* = 1.8 Å^2^, *D*_NS_ = 490 meV·Å^2^, and *β* = 3.3 Å^2^. The calculated dispersion curves with these three parameter sets are very close in the limited range of *Q* between 0.08Å and 0.25 Å. More extensive studies on bulk Co are strongly recommended.

**Figure 13:**
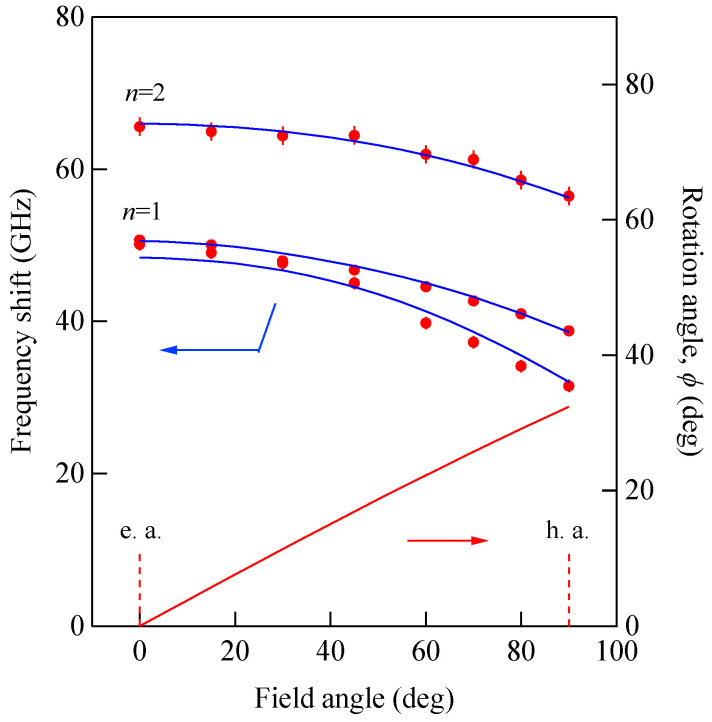
Field angle dependence of the SW frequencies (●) in the scattering geometry (C). The solid lines were calculated for the SW frequencies (−) and the rotation angle *ϕ* of the magnetization (−) using the full magnetic constants given in the text [74]. Here, e.a. and h.a. mean the easy axis (*y*_C_-direction) and the hard axis (*z*_C_-direction) in Figure 10.

The mode profile calculation revealed complicated mode conversion schemes as a function of the magnetic field. The lowest mode possessed a uniform amplitude across the film (*n* = 0 SSW mode) under zero magnetic field and gradually changed into the DE-like mode as field strength increased, finally changing into the *n* = 1 SSW mode at well above *H* = 5.0 kOe. The second-lowest mode retained the *n* = 1 SSW character up to *H*~5.0 kOe and finally changed into the DE mode at well above *H* = 5.0 kOe. Figure 13 shows the SW frequencies obtained from the scattering geometry (C) as a function of the field direction at *H* = 3.0 kOe. 

The solid lines give the calculated SW frequencies and the rotation angle *ϕ* of the magnetization. We also performed the mode profile calculation in this geometry. The lowest-frequency mode at *θ* = 0° was the *n* = 1 SSW, which gradually changed into the 0th SSW mode at *θ* = 90°. The second-lowest mode changed the character from the generalized DE mode at *θ* = 0° into the *n* = 1 SSW mode at *θ* = 90°. As shown in Figure 6, the *n* = 1 SSW mode and the De mode frequencies were very close. In this interpretation, we took account of the anti-crossing effect shown in Figure 11. 

The SW stiffness constants of the Heusler compounds and alloys, Co_2_MnSi, Co_2_MnAl_x_Si_1−x_, Co_2_FeAl, and Co_2_Cr_0_._6_Fe_0_._4_Al were intensively investigated with BLS [76,77,78]. 

### 5.3. Ultrathin Films

Let us define magnetic films with thicknesses less than the exchange length $\ell_{ex}={\left(D/4\pi M\right)}^{1/2}$ [67], which is estimated to be ~30 Å for Fe as an ultrathin film. It is possible to prepare epitaxial ultrathin films by means of the molecular beam epitaxy (MBE) technique. Magnetic properties of such ultrathin films will be strongly affected by the SMA [67]. Here we have a question: what does BLS observe from such ultrathin films? When the film thickness *L* goes to zero, the DE mode frequency is well-separated from the bulk SSW frequencies as shown in Figure 11. Because the surface dispersion parameter *Q*_//_*L* is quite small, the DE mode amplitude is almost uniform across the film. It seems to be preferable to name this the uniform DE (UDE) mode or simply as the uniform mode. I will use the term “UDE mode” in this section. The DE mode was originally discussed for an isotropic slab taking account of the magnetic boundary conditions. The UDE mode is quite different from the slab DE mode because of the SMAs. Then, how can we take into account the SMAs in our discussions? If it is possible, we can positively apply the BLS technique to investigate the SMAs of ultrathin films. 

We consider an ultrathin film with surfaces at *x* = 0 and −*d,* and the LL equation given by

(86) $$\frac{1}{\gamma }\frac{dM}{dt}=M\times \left(H+H_{K}+h_{d}+\frac{D}{M}\frac{d^{2}M}{dx^{2}}\right)$$

Here we ignored the in-plane exchange term. For an ultrathin film, the UDE mode profile is regarded as uniform across the film. Then, we can readily integrate the LL equation across the film and obtain

(87) $$\frac{1}{\gamma }\frac{dM}{dt}=M\times \left(H+H_{K}+h_{d}\right)+\frac{1}{d}\cdot \frac{D}{M}M\times {\frac{dm}{dx}|}_{x=-d}^{x=0}$$

When we notice ∂/∂n=−d/dx in this case, the last term in the right-hand side can be replaced by the Rado-Weertman pinning boundary condition given by Equation (82), thus producing

(88) $$\frac{1}{\gamma }\frac{dM}{dt}=M\times \left(H+H_{K,eff}+h\right)$$

and

(89) $$H_{K,eff}=-\nabla_{M}E_{K}-\frac{2}{d}\nabla_{M}E_{surf}$$

where $H_{K,eff}$ is the effective magnetic anisotropy field consisting of the MAE and IMA SMA terms. We assumed the same SMA for both surfaces. When we adapt the bulk MAE given by Equations (54) and (59) and the SMA given by Equation (83), we obtain

(90) $$\frac{1}{\gamma }\frac{dm_{x}}{dt}-\left[H-\frac{2}{M}\left(K_{//}+\frac{2k_{//}}{d}\right)\right]m_{y}+Mh_{dy}=0$$

and

(91) $$\frac{1}{\gamma }\frac{dm_{y}}{dt}+\left[H-\frac{2}{M}\left(K_{⊥}+\frac{2k_{⊥}}{d}\right)\right]m_{x}-Mh_{dx}=0$$

With these results, we obtain the UDE frequency as follows:

(92) $${\left(\frac{\omega }{\gamma }\right)}^{2}=\left[H-\frac{2}{M}\left(K_{//}+\frac{2k_{//}}{d}\right)\right]\left[H-\frac{2}{M}\left(K_{⊥}+\frac{2k_{⊥}}{d}\right)+4\pi M\right]$$

Here, we define the effective saturation magnetization as

(93) $$4\pi M_{\mathrm{eff}}=4\pi M-\frac{2}{M}\left(K_{⊥}+\frac{2k_{⊥}}{d}\right)$$

The 1/*d* factor in Equation (93) gives a multiplication factor of 10^8^/*d* in the angstrom unit, and the SMA term dominates the out-of-plane magnetic anisotropy field for the UDE mode. As already discussed, the 4*πM*_eff_ variable is the essential parameter to distinguish the magnetization state under zero magnetic field. For a positive 4*πM*_eff_, the film is in the in-plane magnetization state under zero magnetic field, and it is in the perpendicular magnetized state for a negative 4*πM*_eff_. 

**Figure 14:**
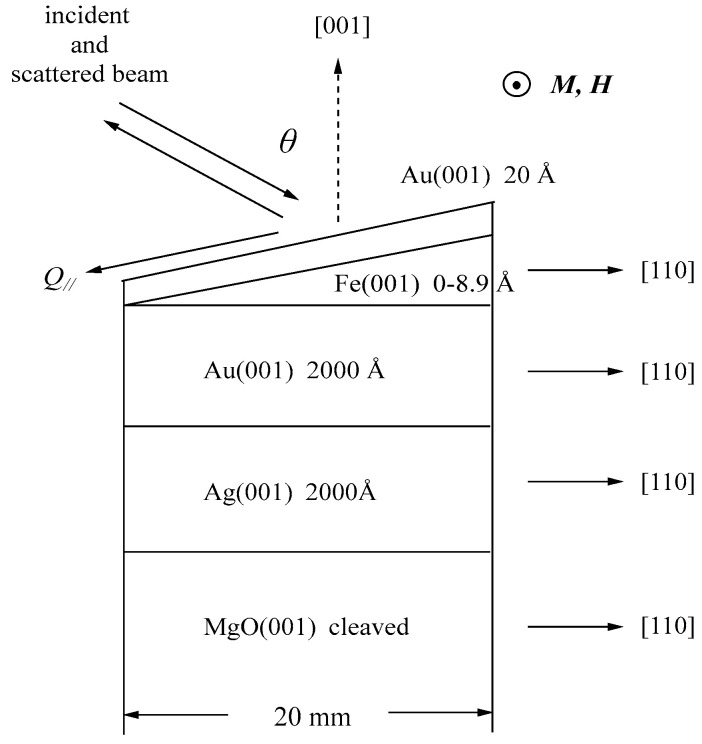
Schematic illustration of the crystallographic wedge structure and scattering geometry [79].

We performed a BLS study on an ultrathin epitaxial Fe wedge with Fe layer thicknesses up to 8.9 Å under the magnetic fields of up to 4.5 kOe [79]. The wedge was prepared at the Electrotechnical Laboratory (ETL), Tsukuba using the MBA technique. Figure 14 shows a schematic illustration of the structure of the MBE-prepared wedge with the crystallographic coordinate systems showing the epitaxial relations.

A 20 Å-thick Au (001) cap layer was deposited to protect the wedge from crucial surface deterioration. We applied the magnetic field along the crystallographic [1–10] direction, and the SWs propagating along the [110] direction were measured. For a cubic symmetry crystal, the MAE is given by

(94) $$E_{K}=\frac{K_{1}}{M^{4}}\left(M_{x}^{2}M_{y}^{2}+M_{y}^{2}M_{z}^{2}+M_{z}^{2}M_{x}^{2}\right)$$

When the magnetization is directed along the [1–10] direction, in terms of the SW variables, the MAE can be written as [35]

(95) $$E_{K}≅\mathrm{const}.-\frac{K_{1}}{2M^{2}}\left(2m_{y}^{2}-m_{x}^{2}\right)$$

Taking account of the possible tetragonal distortion along the surface normal direction, the SMA energy can be reduced into a simple form given by

(96) $$\begin{array}{l} E_{surf}≅-k_{//}^{\left(s\right)}{\left(\frac{m_{y}}{M}\right)}^{2}-\left(k_{u}^{\left(s\right)}-\frac{k_{//}^{\left(s\right)}}{2}\right){\left(\frac{m_{x}}{M}\right)}^{2}\\ \;\;=-k_{//}^{\left(s\right)}{\left(\frac{m_{y}}{M}\right)}^{2}-k_{⊥}^{\left(s\right)}{\left(\frac{m_{x}}{M}\right)}^{2}, \end{array}$$

in which $k_{u}^{\left(s\right)}$ is the uniaxial out-of-plane SMA constant due to tetragonal distortion. 

**Figure 15:**
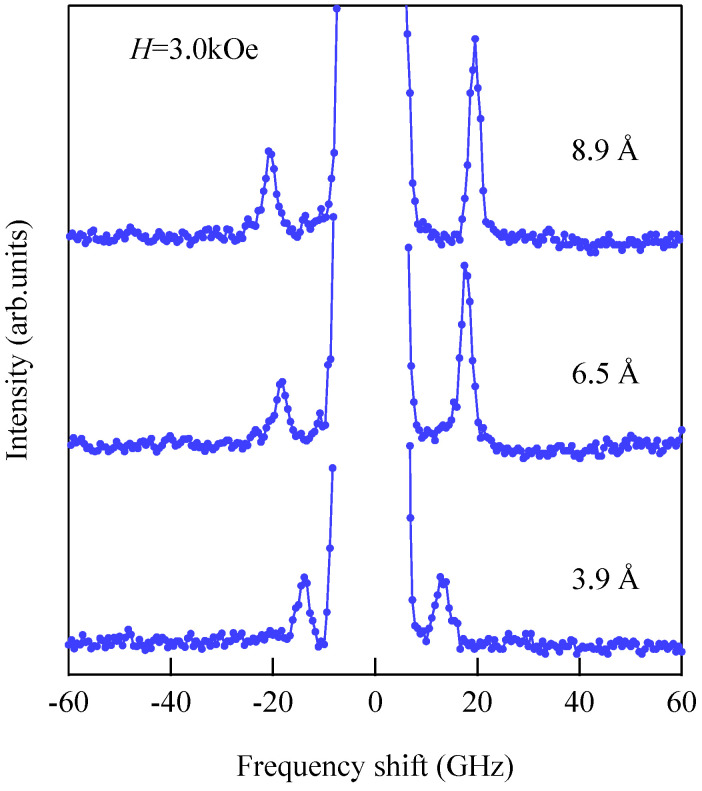
Thickness development of BLS spectra observed at *H* = 3.0 kOe. The thickness was indicated on each spectrum [79].

Figure 15 shows the thickness development of the BLS spectra of the wedge at *H* = 3.0 kOe. The thickness was indicated on each spectrum. 

**Figure 16:**
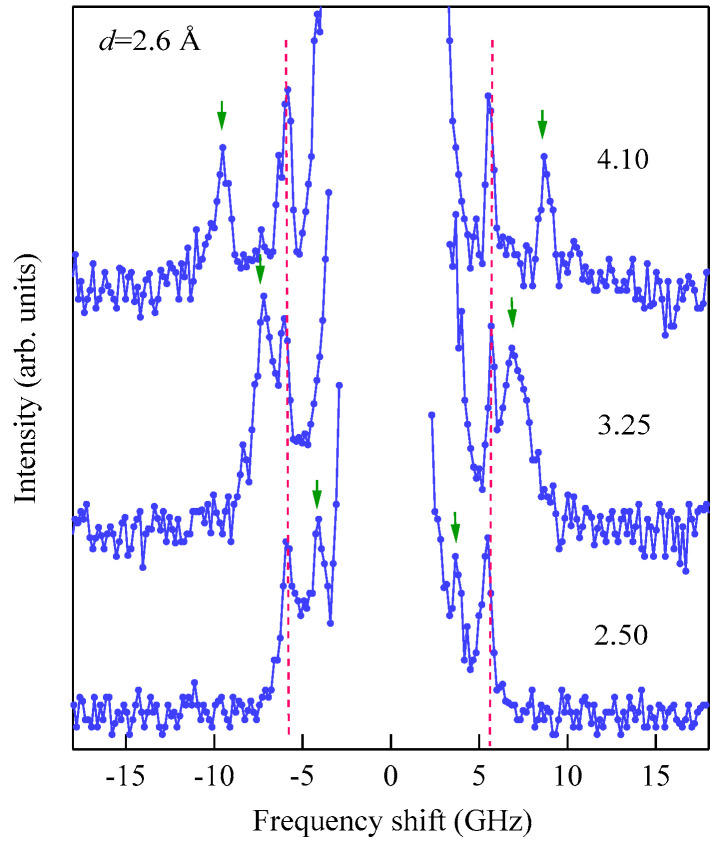
Magnetic field development of BLS spectra observed at *d* = 2.6 Å. The applied field is indicated on each spectrum. SW peaks are indicated by arrows. The peaks on the broken lines are due to the Rayleigh surface acoustic waves, which mainly reflect the elastic properties of the Au/Ag/MgO structure [79].

These spectra were excited by the *p*-polarized 4880 Å line of an Ar^+^ ion laser in a single-cavity mode with a power of 80 mW directed at the wedge. The incident angle ϑ was fixed to 45° (*Q*_//_ = 1.82 × 10^5^ cm^−1^). The Fe layer thickness was thin enough to observe scattering from the UDE modes existing on both surfaces. Typical accumulation time for a spectrum was only less than 1 h. Because we had not performed polarization selection, both the USW and SAW peaks were observed in a spectrum. The SAW peaks were masked by the Rayleigh peak in Figure 15. The intensity asymmetry between the Stokes side and the anti-Stokes side is one of the characteristic features of SW scattering, as already discussed. The UDE frequency rapidly decreased from about 20 GHz to 12 GHz with decreasing Fe thickness. Figure 16 shows the external field development of BLS spectra observed at an Fe thickness of *d* = 2.6 Å. 

**Figure 17:**
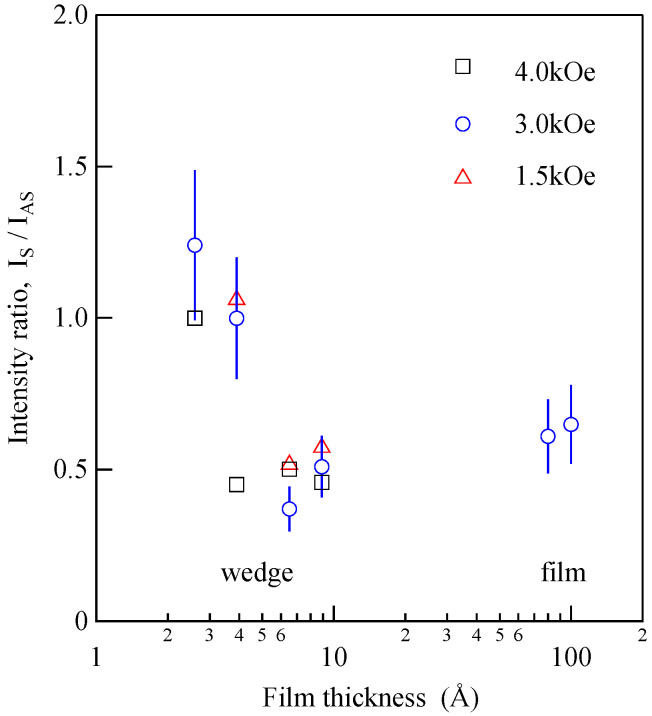
Film thickness and magnetic field dependences of the intensity ratio between the Stokes peak and the anti-Stokes peak of the Au/Fe/Au films and the wedge [61].

The applied magnetic field was indicated on each spectrum. The UDE peaks are indicated by the arrows, and the SAW peaks are indicated by the broken lines. The sharp UDE peaks shown in Figure 15 and Figure 16 indicate that a well-defined ferromagnetic order was realized in the wedge at room temperature even at 2.6 Å, which corresponds to a ~1.8 atomic layer. Taking into account the extremely short photon–UDE interaction length, although multiple reflections would take place, the large BLS efficiency seems to have been closely related to the enhancement of the magneto-optical Kerr rotation and ellipticity observed around 2.5 eV of incident photon energy due to the plasma-edge effect of the Au layers [80]. This energy is close to our 4880 Å laser photon energy (2.54 eV). Figure 17 shows the intensity ratio between the Stokes peak and the anti-Stokes peak. 

Above *d* > 40 Å, the intensity ratio was about 0.5 in a wide range of Fe thicknesses, whereas it became larger than 1 below *d* < 40 Å. The intensity ratio for the 45° incident *p*-polarized geometry can be written as follows in terms of the matrix components defined in Equations (15)–(18):

(97) $$\frac{I_{S}}{I_{aS}}=\frac{{\left|\xi_{31}^{-}-\varsigma_{32}^{-}\right|}^{2}}{{\left|\xi_{31}^{+}-\varsigma_{32}^{+}\right|}^{2}}$$

As shown in Equation (29), the *K* coefficient and possibly also the *G* coefficient are sensitive to the spin-orbit coupling constant and the wave functions for the ground state and intermediate state. Because the ultrathin Fe layer was sandwiched by the thicker Au layers, the Fe wave functions were strongly modified by mixing between the Au wave functions at *d* < 40 Å. This situation can be regarded as a quantum well effect.

**Figure 18:**
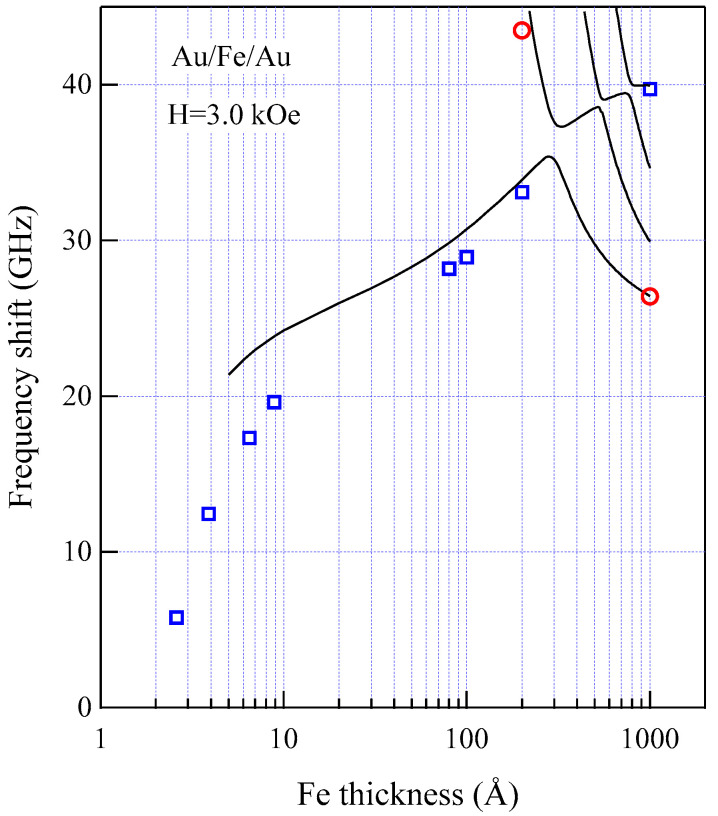
SW frequencies (□ for the DE mode, ○ for the *n* = 1 SSW mode) observed from the SWs propagating along the [110] direction on the epitaxial (001) thin Fe films and Fe wedge as a function of the Fe thickness. The solid lines are the calculated SW frequencies, and the results above *d* ≥ 1 nm are already shown in Figure 11. Although the MAE and the effective demagnetization factor were included in the calculation, the surface perpendicular anisotropy term was not included [61]. The broken lines are just for eye.

Figure 18 shows the thickness development of the SW frequencies of the Au/Fe/Au films, including the wedge and the epitaxial films, with thicknesses between 80 and 1000 Å for *H* = 3.0 kOe. 

**Figure 19:**
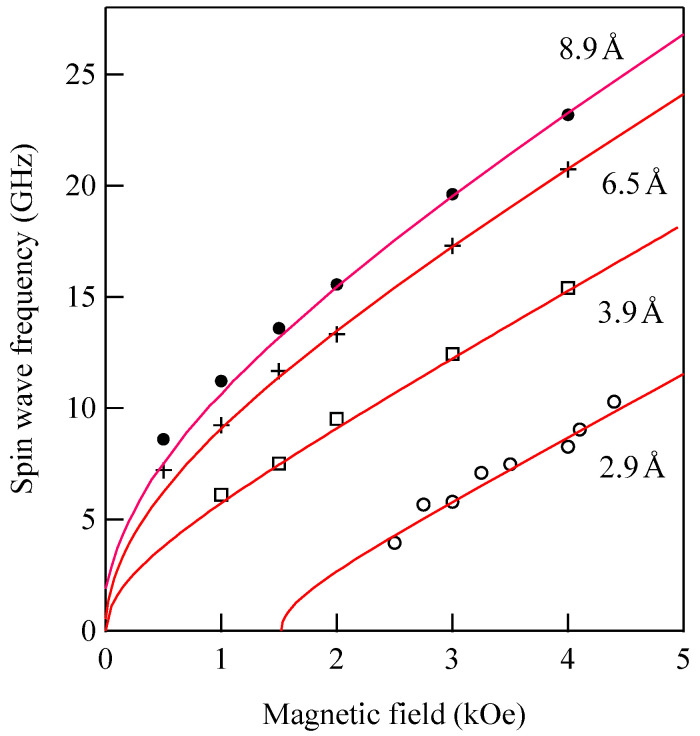
SW frequencies for four Fe thicknesses, 8.9, 6.5, 3.9, and 2.6 Å, as a function of the magnetic field. The solid lines are the SW frequencies calculated by solving numerically the BCD equation [79].

These thin Au/Fe/Au epitaxial films were also prepared at ETL, Tsukuba using the MBE technique. The solid circles are the DE and UDE mode frequencies, and the open circles are the 1-st SSW frequencies. The solid lines are the calculated SW frequencies with the same magnetic constants used in Figure 11. It is obvious that the observed UDE frequencies decreased more rapidly than the calculated frequency as the Fe thickness decreased below 100 Å. In contrast to the present Au/Fe/Au films, for the ultrathin Fe (110) film deposited on the W (110) substrate, the UDE frequency increased with decreasing Fe thickness because of the negative $k_{⊥}^{\left(s\right)}$ constant (this means that the surface normal is the hard direction) [34,81]. Because we have not included the SMA terms in Equations (92) and (93) in our calculations, our calculations gave higher SW frequencies below 100 Å. Figure 19 shows the UDE frequencies as a function of the external magnetic field for four Fe thicknesses: 8.9, 6.5, 3.9, and 2.6 Å. 

**Figure 20:**
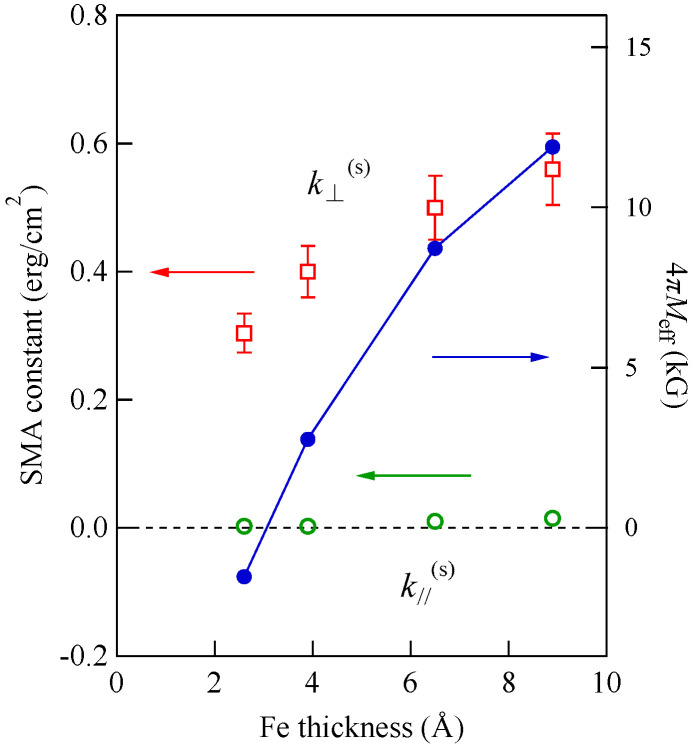
SMA constants for $k_{⊥}^{\left(s\right)}$ (□) and $k_{//}^{\left(s\right)}$ (○) and 4*πM*_eff_ (●) for four Fe thicknesses. The solid lines are just for eye [79].

In order to analyze these results, we solved numerically the 6 × 6 BCD equation with the SMA terms given by Equation (95). The solid lines in Figure 19 are the calculated UDE frequencies by solving the BCD equation as a function of the magnetic field. Because we found that the bulk MAE was negligibly small, we retained only the SMA terms and a bulk spin wave stiffness constant of *D* = 2.34 × 10^−9^ Oe·cm^2^ throughout the present BCD analyses, which can also be applied for the in-plane magnetized films. For *d* = 2.6 Å, we can solve the BCD equation above *H* = 1.57 kOe. Figure 20 shows the in-plane and out-of-plane SMA constants and the 4*πM*_eff_ defined in Equation (93) as a function of Fe thickness. Because the in-plane SMA constant $k_{//}^{\left(s\right)}$ was negligibly small, as shown in Figure 20, the out-of-plane SMA constant $k_{⊥}^{\left(s\right)}$ stems from the uniaxial SMA constant $k_{u}^{\left(s\right)}$, which reflects the tetragonal distortion of the bcc Fe layer. Although $k_{⊥}^{\left(s\right)}$ gradually decreased with decreasing Fe thickness, the *d*^−1^ factor in Equation (93) increased much faster than the decrease of $k_{⊥}^{\left(s\right)}$. Therefore, 4*πM*_eff_ changes its sign from positive to negative at around *d*~3 Å, and the stable direction of the magnetization under zero magnetic field turns over from the in-plane direction to the surface normal direction. The in-plane to out-of-plane transition of the stable magnetization configuration under zero magnetic field has been observed in several ultrathin films [81,82,83,84].

**Figure 21:**
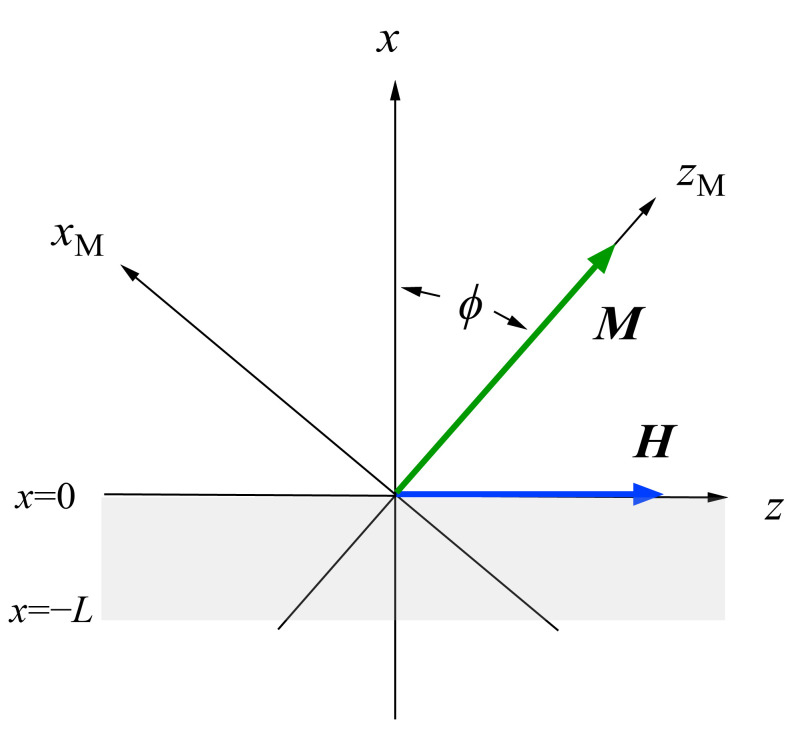
Schematic illustration on the magnetization rotation by the external magnetic field *H* applied the in-plane *z* direction (*H* ≤ *H*_C_). The equilibrium angle *ϕ* can be determined by Equation (103). By counterclockwise *π*/2-*ϕ* rotation around the *y*-axis, we can introduce the magnetization-based coordinates (*x*_M_, *y*_M_, *z*_M_). In this coordinate choice, we can decouple the LL equation of motion on the *z*_M_ component from the set of equations on the *x*_M_ and *y*_M_ components.

Apart from the ultrathin films, let us consider SWs in magnetic films with the out-of-plane MAE as given by Equation (59). Dutcher et al. [82] and Rahman and Mills [85] treated this problem using the magnetostatic framework. Here, the *K*_⊥_ coefficient can be regarded as an effective constant including both the bulk and surface terms, which depend on the crystallographic structure of the magnet and the film thickness. The magnet occupies the *x*–*z* plane between *x* = 0 and –*L*, and the applied magnetic field is always fixed to the *z*-axis. The magnetization always lies in the *x*–*z* plane with the equilibrium angle *ϕ* measured from the *x*-axis, as shown in Figure 21. 

We adapted the LL equation of motion with the effective fields given by Equation (66). Because the magnetization ***M*** is tilted in the *x*–*z* plane, we should add the demagnetization field *H*_d_ term given by

(98) $$H_{d}=-4\pi \left(M\cos\phi +m_{x},\;0,\;0\right)$$

Then, we obtain a set of linearized LL equations:

(99) $$\frac{1}{\gamma }\frac{dm_{x}}{dt}=\left(H-D\sin\phi \nabla^{2}\right)m_{y}-M\sin\phi \;h_{y}$$

(100) $$\begin{array}{l} \frac{1}{\gamma }\frac{dm_{y}}{dt}=M\cos\phi \left[H_{C}\sin\phi -H\right]\\ \;\;\;+\cos\phi \;\left(H_{C}-D\nabla^{2}\right)\;m_{z}+D\sin\phi \nabla^{2}m_{x}+M\left(\sin\phi \;h_{x}-\cos\phi \;h_{z}\right), \end{array}$$

and

(101) $$\frac{1}{\gamma }\frac{dm_{z}}{dt}=-\cos\phi \;\left(H_{C}-D\nabla^{2}\right)\;m_{y}+M\cos\phi \;h_{y}$$

Here, we define the critical field *H*_C_ as

(102) $$H_{C}=\frac{2K_{⊥}}{M}-4\pi M=H_{K⊥}-4\pi M$$

The equilibrium angle ϕ is determined from the torque-free condition around the *y* axis given by

(103) $$\cos\phi \left[H_{C}\sin\phi -H\right]=0$$

We obtain three angles:

(1) *ϕ* = π/2 for 4*πM* > *H*_K⊥_,
(2) *ϕ* = sin^−1^(*H*/*H*_C_) for *H*_C_ ≥ *H* > 0,

and

(3) *ϕ* = π/2 for *H* > *H*_C_ > 0.

We introduce a 3 × 3 susceptibility matrix *χ* by *m* = χ⋅*h* through the LL equation. For the present scattering geometry shown in Figure 21, among the nine components of *χ*, four components,$\chi_{xx},\;\chi_{xy},\;\chi_{yx},\;\mathrm{and}\;\chi_{yy}$, are relevant, and their explicit expressions are given as

(104) $$\chi_{xx}\left(\omega \right)=M\frac{\left(H+DQ^{2}\sin\phi \right)\;\sin\phi }{{\left(\Omega /\gamma \right)}^{2}-{\left(\omega /\gamma \right)}^{2}},$$

(105) $$\chi_{yy}\left(\omega \right)=M\frac{\left(H_{K}-4\pi M+DQ^{2}\right)\cos^{2}\phi +\left(H-\left(H_{K}-DQ^{2}\right)\sin\phi \right)\sin\phi }{{\left(\Omega /\gamma \right)}^{2}-{\left(\omega /\gamma \right)}^{2}},$$

and

(106) $$\chi_{yx}=-\chi_{xy}=M\frac{i\left(\omega /\gamma \right)\sin\phi }{{\left(\Omega /\gamma \right)}^{2}-{\left(\omega /\gamma \right)}^{2}}$$

We also define

(107) $${\left(\Omega /\gamma \right)}^{2}=\left[H-\left(H_{K}-DQ^{2}\right)\sin\phi \right]{\left(H+DQ^{2}\sin\phi \right)}^{}+\cos^{2}\phi \;{\left(H_{K}-4\pi M+DQ^{2}\right)}^{2}$$

We have obtained the relevant susceptibilities, and we can follow our calculations performed for the in-plane thin films. Therefore, we will not repeat them here. When we ignore the exchange terms, we can summarize our results on the bulk SW as follows:

For 4*πM* > *H*_K⊥_

(108) $${\left(\frac{\omega }{\gamma }\right)}^{2}=H\left(H+4\pi M-H_{K}\right)-4\pi MH_{K}\frac{Q_{//}^{2}}{Q^{2}}$$

The upper and lower bound frequencies are given by

(109) $$H\left(H+4\pi M-H_{K⊥}\right)\;\;\;\;\;\;\text{:}Q_{//}^{2}/Q^{2}=0\;$$

and

(110) $$\left(H-H_{K⊥}\right)\left(H+4\pi M\right)\;\;\;\;\;\;\text{:}Q_{//}^{2}/Q^{2}=1$$

For *H* > *H*_C_ > 0

(111) $${\left(\frac{\omega }{\gamma }\right)}^{2}=H\left(H-H_{C}\right)-4\pi MH_{K}\frac{Q_{//}^{2}}{Q^{2}}$$

The upper and lower bound frequencies are given by

(112) $$H\left(H-H_{C}\right)\;\;\;\;\;\;\text{:}Q_{//}^{2}/Q^{2}=0\;$$

and

(113) $$\left(H-H_{K⊥}\right)\left(H+4\pi M\right)\;\;\;\;\;\;\text{:}Q_{//}^{2}/Q^{2}=1$$

and for *H*_C_ ≥ *H* > 0

(114) $${\left(\frac{\omega }{\gamma }\right)}^{2}=H_{C}{}^{2}-H^{2}+\frac{Q_{//}^{2}}{Q^{2}}\left[H_{C}\left(H_{K}-H_{C}\right)-\frac{H_{K}^{2}}{H_{C}^{2}}H^{2}+H^{2}\right]$$

We have two boundary frequencies given by

(115) $$H_{C}^{2}-H^{2}\;\;\;\;\;\;\text{:}Q_{//}^{2}/Q^{2}=0\;$$

and

(116) $$H_{C}H_{K}-\frac{H_{K}^{2}}{H_{C}^{2}}H^{2}\;\;\;\;\;\;\text{:}Q_{//}^{2}/Q^{2}=1$$

Note that these boundaries are crossed at $H=H_{C}{\left[H_{C}/\left(H_{C}+H_{K⊥}\right)\right]}^{1/2}$ in Equation (114). We also performed numerical fits using the upper bounds in Equations (108) and (111) with our BLS results shown in Figure 19, and we obtained reasonable agreement between the calculated and observed SW frequencies.

The discussions on the DE and UDE modes are a little more complicated. The surface mode frequency is determined by a BCD equation given as

(117) $$\left|\begin{array}{cc} \alpha \left(1+4\pi \chi_{xx}\right)-\left(1-i4\pi \chi_{xy}\right) & \alpha \left(1+4\pi \chi_{xx}\right)+\left(1-i4\pi \chi_{xy}\right)\\ \alpha \left(1+4\pi \chi_{xx}\right)+\left(1+i4\pi \chi_{xy}\right) & \left[\alpha \left(1+4\pi \chi_{xx}\right)-\left(1+i4\pi \chi_{xy}\right)\right]\beta^{2} \end{array}\right|=0$$

Here we define $q_{⊥}=\alpha Q_{//}={\left[\left(1+4\pi \chi_{yy}\right)/\left(1+4\pi \chi_{xx}\right)\right]}^{1/2}Q_{//}$ and $\beta^{2}=\exp\left(-2\alpha Q_{//}L\right)$. We rewrite Equation (117) as

(118) $$\;\tanh\left(\alpha Q_{//}L\right)=-\frac{2\alpha \left(1+4\pi \chi_{xx}\right)}{\alpha^{2}{\left(1+4\pi \chi_{xx}\right)}^{2}+\left(1-i4\pi \chi_{xy}\right)\left(1+i4\pi \chi_{xy}\right)}$$

Note that the case of tanh(*αQ*_//_*L*) = 0 corresponds to an ultrathin film, and the case of tanh(*αQ*_//_*L*) = 1 corresponds to a semi-infinite magnet. It is not difficult to show that the DE and UDE modes are always allowed for in-plane films (*ϕ* = *π*/2) with the frequency given by Equation (62). On the other hand, because of negative *α*, these surface modes are forbidden for the out-of-plane films above *H* > *H*_C_.

Next let us consider an out-of-plane film under a magnetic field of *H* ≤ H_C_. For a semi-infinite magnet, Equation (115) becomes a simple form given by

(119) $$\left[\alpha \left(1+4\pi \chi_{xx}\right)+\left(1+i4\pi \chi_{xy}\right)\right]\;\left[\alpha \left(1+4\pi \chi_{xx}\right)+\left(1-i4\pi \chi_{xy}\right)\right]=0$$

**Figure 22:**
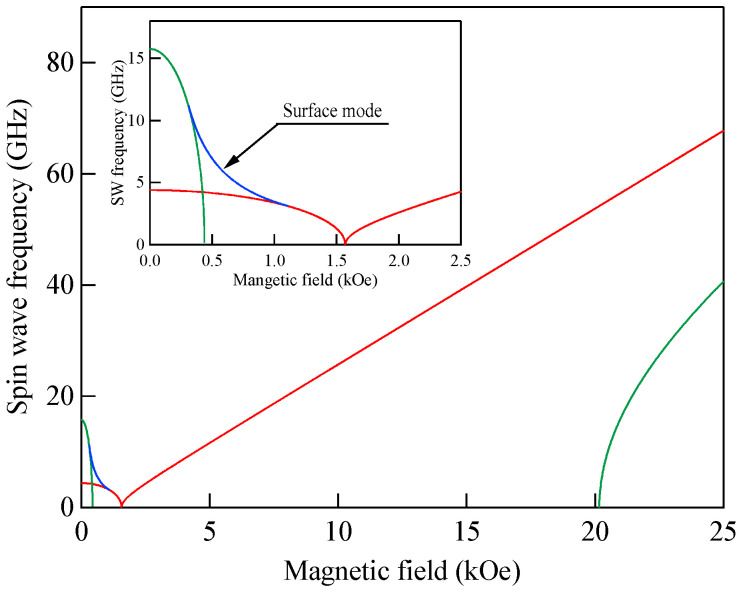
A simulation of the SW frequencies for an out-of-plane magnetized ferromagnetic slab as a function of the magnetic field. Here, the red lines and the green lines show the upper and lower bound of the bulk SW band. We used the magnetic constants obtained from the Fe wedge given in the text. The inset shows the lower field result below 2.5 kOe. Note the boundary crossing below *H*~0.4 kOe.

These equations can be analytically solved and give solutions with opposite signs. We solved the first one here and obtained a surface mode solution with a frequency given by $\omega /\gamma =H_{C}^{2}/2H$ in a limited range of the external field $\sqrt{H_{C}/2H_{K}}<H/H_{C}<1/\sqrt{2}$. Figure 22 shows a simulation of the SW frequencies as a function of the magnetic field for an out-of-plane, magnetic, semi-infinite slab calculated using the magnetic constants obtained from the Fe wedge:

$$\gamma /2\pi =2.8\;\mathrm{GHz}/\mathrm{kOe},\;H_{C}=1.57\;\mathrm{kOe},\;4\pi M=18.6\;\mathrm{kG},\;\mathrm{and}\;H_{k}=20.17\;\mathrm{kOe}.$$

For an ultrathin film, Equation (118) merely gives the bulk upper and lower bounds, and we have no UDE solution, as already discussed. For a finite thickness film, we must solve Equation (118) numerically, but we will discuss no more on this case.

### 5.4. Multilayers and Superlattices

**Figure 23:**
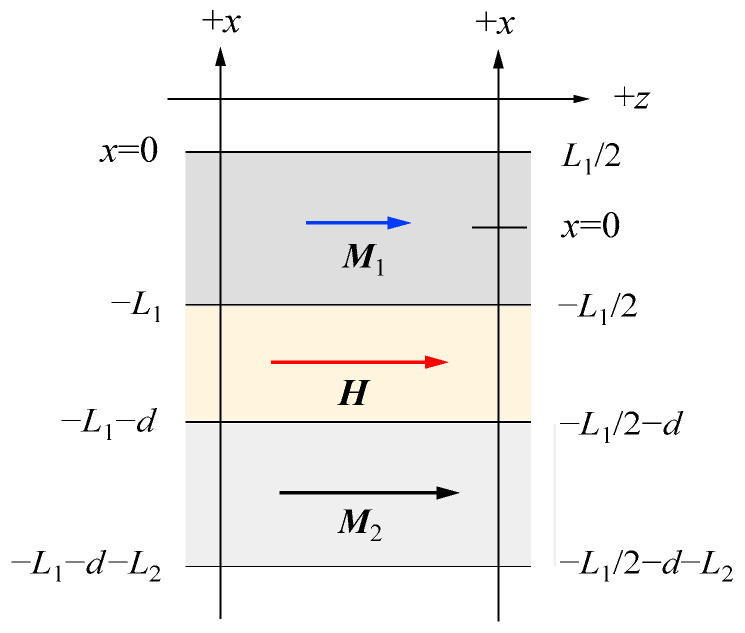
Schematic illustration of a (M1/spacer/M2) trilayer structure and the *x*-axis setting. We set *x* = 0 on top of the M1 layer in the first setting and in the center of the M1 layer in the second setting.

Figure 23 shows an example of a trilayer structure consisting of a nonmagnetic layer sandwiched between magnetic layers [35].

These magnetic layers need not be the same with respect to their thicknesses and materials [36]. The thicknesses of the magnetic layers are usually set to be less than 100 Å in order to fully separate the bulk SSWs and the DE mode as shown in Figure 11. In our following discussions based on [36], we consider the SWs in the trilayer by combining the DE modes in each magnetic layer. We have two choices on the origin of the *x*-coordinate. In this example, we use the first setting shown on the left-hand side of Figure 23. 

At first, we consider the SWs propagating along the *y*-axis in Figure 23 and solve the LL equation for each magnetic layer in terms of the susceptibilities and the magnetic potentials as follows:

(120) $$m_{x}^{\left(j\right)}=\chi_{xx}^{\left(j\right)}h_{x}^{\left(j\right)}+\chi_{xy}^{\left(j\right)}h_{y}^{\left(j\right)}=-\left(\chi_{xx}^{\left(j\right)}\frac{\partial }{\partial x}+\chi_{xy}^{\left(j\right)}\frac{\partial }{\partial y}\right)\;\varphi^{\left(j\right)}$$

and

(121) $$m_{y}^{\left(j\right)}=\chi_{yx}^{\left(j\right)}h_{x}^{\left(j\right)}+\chi_{yy}^{\left(j\right)}h_{y}^{\left(j\right)}=-\left(\chi_{yx}^{\left(j\right)}\frac{\partial }{\partial x}+\chi_{yy}^{\left(j\right)}\frac{\partial }{\partial y}\right)\;\varphi^{\left(j\right)}$$

Here, the superscript *j* (=1, 2) specifies the magnetic layer. The magnetic potentials outside and inside of the trilayer are given as follows:

(122) $$\varphi =\varphi_{>}\exp\left(i\omega t-Q_{//}x\pm iQ_{//}y\right)$$

(123) $$\varphi_{M1}=\left(A\exp\left(-Q_{//}x\right)+B\exp\left(Q_{//}x\right)\right)\exp\left(i\omega t\pm iQ_{//}y\right)$$

(124) $$\varphi_{NM}=\left(C\exp\left(-Q_{//}x\right)+D\exp\left(Q_{//}x\right)\right)\exp\left(i\omega t\pm iQ_{//}y\right)$$

(125) $$\varphi_{M2}=\left(E\exp\left(-Q_{//}x\right)+F\exp\left(Q_{//}x\right)\right)\exp\left(i\omega t\pm iQ_{//}y\right)$$

and

(126) $$\varphi =\varphi_{<}\exp\left(i\omega t+Q_{//}x\pm iQ_{//}y\right)$$

The magnetic boundary conditions at each surface and interface give us a set of six homogeneous equations for the unknown variables *A* to *F*. To obtain the non-trivial solutions of the set of the homogeneous equations, the BCD should vanish at the SW frequencies.

(127) $$\left|\begin{array}{cccccc} \Lambda_{1}^{\left(+\right)}-1 & -\left(\Lambda_{1}^{\left(-\right)}+1\right) & 0 & 0 & 0 & 0\\ 1 & \alpha & -1 & -\alpha & 0 & 0\\ \Lambda_{1}^{\left(+\right)} & -\alpha \Lambda_{1}^{\left(-\right)} & -1 & \alpha & 0 & 0\\ 0 & 0 & 1 & \beta & -1 & -\beta \\ 0 & 0 & 1 & -\beta & -\Lambda_{2}^{\left(+\right)} & \beta \Lambda_{2}^{\left(-\right)}\\ 0 & 0 & 0 & 0 & \Lambda_{2}^{\left(+\right)}+1 & -\gamma \left(\Lambda_{2}^{\left(-\right)}-1\right) \end{array}\right|=0$$

in which we defined

(128) $$\alpha =\exp\left(-2Q_{//}L_{1}\right)$$

(129) $$\beta =\exp\left(-2Q_{//}\left(L_{1}+d\right)\right)$$

(130) $$\gamma =\exp\left(-2Q_{//}\left(L_{1}+d+L_{2}\right)\right)$$

and

(131) $$\Lambda_{\pm }^{\left(j\right)}=1+4\pi \chi_{xx}^{\left(j\right)}\pm i4\pi \chi_{xy}^{\left(j\right)}$$

For isotropic (no MAE) magnetic layers, the SW frequencies are given by the solution of Equation (127) as below:

(132) $$\begin{array}{l} \exp\left(-2Q_{//}d\right)\left[1-\exp\left(-2Q_{//}L_{1}\right)\right]\;\left[1-\exp\left(-2Q_{//}L_{2}\right)\right]\left(2\pi M_{1}\right)\left(2\pi M_{2}\right)\\ \times \left\{\left(H+2\pi M_{1}\pm \frac{\omega }{\gamma_{1}}\right)\left(H+2\pi M_{2}\mp \frac{\omega }{\gamma_{2}}\right)\right\}\\ =\left\{{\left(H+2\pi M_{1}\right)}^{2}-{\left(2\pi M_{1}\right)}^{2}\exp\left(-2Q_{//}L_{1}\right)-{\left(\frac{\omega }{\gamma_{1}}\right)}^{2}\right\}\\ \times \left\{{\left(H+2\pi M_{2}\right)}^{2}-{\left(2\pi M_{2}\right)}^{2}\exp\left(-2Q_{//}L_{2}\right)-{\left(\frac{\omega }{\gamma_{2}}\right)}^{2}\right\}. \end{array}$$

**Figure 24:**
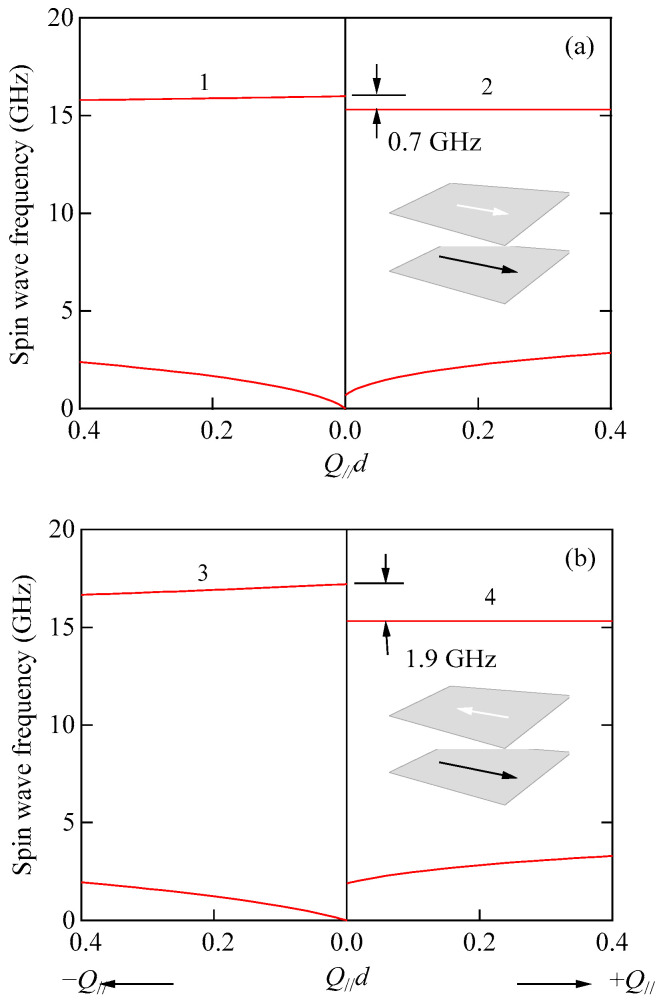
The solid lines are calculated SW frequencies in the (M1/spacer/M2) trilayer at zero magnetic field as a function of *Q*_//_*d* in which *d* is the spacer thickness. (**a**) Parallel arrangement and (**b**) anti-parallel arrangement. The magnetic and layer parameters used in the calculations are given in the text.

The composite symbols in Equations (131) and (132) correspond to the SW propagation directions given by exp(±*iQ*_//_*y*). It is clear that for identical magnetic layers, Equation (132) gives two SW frequencies which are independent of the propagation direction. When the surface dispersion parameter *Q*_//_*d* is zero, the SW frequencies are given by the DE mode frequency for a 2*L* thick film (set *L*→2*L* in Equation (45)), and the bulk SW frequency is given by Equation (51). For the large *Q*_//_*d* values, the SW frequencies approach to the DE mode frequency is given by Equation (45) for two different isolated magnetic layers. Now, we consider parallel and anti-parallel arrangements of two different isotropic magnetic layers [35]. Figure 24a shows the SW frequencies as a function of the *Q*_//_*d* parameter for the parallel arrangement under zero magnetic field. 

In the calculations, we set the surface wave vector *Q*_//_ to 2.0 × 10^−7^ m^−1^ and used 4*πM*_1_ = 10.6 kG, *g* = 2.13, and *L*_1_ = 50 Å for layer 1 (CoNbZr) and 4*πM*_2_ = 19.8 kG, *g* = 2.17, and *L*_2_ = 150 Å for layer 2 (Co). Figure 24b shows the SW frequencies as a function of the *Q*_//_*d* parameter for the anti-parallel arrangement under zero magnetic field. It is important to note that the anti-parallel arrangement of the magnetizations is unstable even for weak external magnetic fields. We have various spin valve devices [86]. The spin valve structure consists of the pinned layer, in which the magnetization is pinned to prevent free motion against the external magnetic field, and the free layer, in which the magnetization can be freely aligned along the external field even under a weak magnetic field. Because the trilayer consists of two different magnets, the BLS spectrum is eventually asymmetric between the Stokes and anti-Stokes sides for the *Q*_//_*d* parameters below ~1. The frequency difference between levels 3 and 4 in the anti-parallel arrangement was 1.9 GHz, whereas the difference between levels 1 and 2 was 0.7 GHz in the parallel arrangement. Note that the frequency difference of 0.7 GHz is rather difficult to detect by means of the BLS technique; of course, it thus depends on the free spectral range setting. The lower frequency peaks were be masked by the intense Rayleigh peak. When the magnetization arrangement was changed from the parallel to anti-parallel arrangements, the frequency difference between the levels 3 and 1 was 2 GHz, and it was easily detected by the BLS technique.

For thinner spacer layers with thicknesses below the exchange length, we must take into account the interlayer exchange coupling (IEC) between the magnetic layers across the spacer layer [40]. The simplest form of IEC is given by the Heisenberg-type coupling given as

(133) $$H_{\mathrm{IEC}}=-\frac{A_{12}}{M_{1}M_{2}}\left(M_{1}\cdot M_{2}\right)$$

in which the IEC constant *A*_12_ depends on the spacer layer thickness *d* and changes its sign similar to the Ruderman–Kittel–Kasuya–Yoshida (RKKY) coupling [87]. For a positive *A*_12_, the parallel arrangement of *M*_1_ and *M*_2_ is preferable, and the anti-parallel arrangement is preferable for negative *A*_12_. The equations of motion for *M*_1_ and *M*_2_ are given as

(134) $$\frac{1}{\gamma }\frac{dM_{1}}{dt}=\frac{A_{12}}{M_{1}M_{2}}\left(M_{1}\times M_{2}\right)$$

and

(135) $$\frac{1}{\gamma }\frac{dM_{2}}{dt}=\frac{A_{12}}{M_{1}M_{2}}\left(M_{2}\times M_{1}\right)$$

The ICE acts as torque for the magnetization on each layer. We can take into account the torque through the Hoffmann boundary conditions [27] at the magnet–spacer interfaces. The Hoffmann boundary conditions are given by

(136) $$M_{1}\times \left[\frac{2A_{1}}{M_{1}^{2}}\frac{\partial M_{1}}{\partial n}-\nabla_{M}E_{\mathrm{int}}^{\left(1\right)}\right]+\frac{2A_{12}}{M_{1}M_{2}}M_{1}\times M_{2}=0$$

and

(137) $$M_{2}\times \left[\frac{2A_{2}}{M_{2}^{2}}\frac{\partial M_{2}}{\partial n}-\nabla_{M}E_{\mathrm{int}}^{\left(2\right)}\right]+\frac{2A_{12}}{M_{1}M_{2}}M_{2}\times M_{1}=0$$

Here, *A*_j_ (=*D*_j_*M*_j_/2) is the intra-layer exchange stiffness constant in the *j*-th magnetic layer, and ∂/∂*n* is always directed to the inside of the magnets. For brevity’s sake, we adapted the same energy form for both of the interfacial magnetic anisotropy energy (IME) and the SME.

(138) $$E_{\mathrm{int}}^{\left(j\right)}=-\lambda_{⊥}^{\left(j\right)}\;{\left(\frac{m_{x}^{\left(j\right)}}{M_{j}}\right)}^{2}-\lambda_{//}^{\left(j\right)}{\left(\frac{m_{y}^{\left(j\right)}}{M_{j}}\right)}^{2}\left(j=1,2\right)$$

After some calculations, we obtained the explicit expressions of the linearized Hoffmann boundary conditions as follows [40]:

For the magnetic layer 1:

(139) $${\left(\frac{A_{1}}{M_{1}}\frac{\partial m_{y}^{\left(1\right)}}{\partial x}+\frac{\lambda_{//}^{\left(1\right)}}{M_{1}}m_{y}^{\left(1\right)}-\frac{A_{12}}{M_{1}}m_{y}^{\left(1\right)}\right)|}_{x=-L_{1}}+\frac{A_{12}}{M_{2}}{m_{y}^{\left(2\right)}|}_{x=-L_{1}-d}=0$$

and

(140) $${\left(\frac{A_{1}}{M_{1}}\frac{\partial m_{x}^{\left(1\right)}}{\partial x}+\frac{\lambda_{⊥}^{\left(1\right)}}{M_{1}}m_{x}^{\left(1\right)}-\frac{A_{12}}{M_{1}}m_{x}^{\left(1\right)}\right)|}_{x=-L_{1}}+\frac{A_{12}}{M_{2}}{m_{x}^{\left(2\right)}|}_{x=-L_{1}-d}=0$$

For the magnetic layer 2:

(141) $${\left(\frac{A_{2}}{M_{2}}\frac{\partial m_{y}^{\left(2\right)}}{\partial x}-\frac{\lambda_{//}^{\left(2\right)}}{M_{2}}m_{y}^{\left(2\right)}+\frac{A_{12}}{M_{2}}m_{y}^{\left(2\right)}\right)|}_{x=-L_{1}-d}-\frac{A_{12}}{M_{1}}{m_{y}^{\left(1\right)}|}_{x=-L_{1}}=0$$

and

(142) $${\left(\frac{A_{2}}{M_{2}}\frac{\partial m_{x}^{\left(2\right)}}{\partial x}-\frac{\lambda_{⊥}^{\left(2\right)}}{2M_{2}}m_{x}^{\left(2\right)}+\frac{A_{12}}{M_{2}}m_{x}^{\left(2\right)}\right)|}_{x=-L_{1}-d}-\frac{A_{12}}{M_{1}}{m_{x}^{\left(1\right)}|}_{x=-L_{1}}=0$$

Because the Hoffmann boundary conditions were adapted at the magnet–spacer interfaces and the Rado–Weertman boundary conditions at the top and bottom magnetic surfaces, we must use the dipole-exchange framework which has been already discussed for thin films and results in a 6 × 6 BCD equation to determine the SW frequencies for a single magnetic film. In the present case, we have two mutually coupled magnetic layers and must solve a 12 × 12 BCD equation to obtain the SW frequencies. We have already given the explicit expressions of *m*_x_ and *m*_y_ in Equations (37) and (38). Grünberg and his coworkers successfully showed the oscillatory behavior of the *A*_12_ constant as a function of the spacer layer thickness in Fe/Cr/Fe trilayers using the BLS technique [40]. They found the giant magnetoresistance (GMR) effect in the anti-parallel state of the layer magnetizations [88]. In the anti-parallel magnetization state, the spin-flop phenomena can be induced by the external magnetic field [89,90]. 

In magnetic multilayers (MMLs) and superlattices (MSLs), a fundamental structure, for example magnetic layer 1 on magnetic layer 2, is stacked an arbitrary number of times. SWs, as a whole of the multilayers and superlattices, are constructed from the DE or UDE mode in each magnetic layer. As an example, we consider a (M1/M2)_3_ multilayer. Here, the (M1/M2)_3_ symbol means that the fundamental unit consisting of magnetic layers M1 and M2 is repeated three times. We can successively apply the appropriate boundary conditions at each interface and solve a BCD equation to obtain the MML and MSL SW frequencies [37]. In this example, we employed the second setting for the origin of the *x*-axis shown in the right-hand side of Figure 23. We set *x* = 0 at the center of the top M1 layer. When we set susceptibilities to zero in the M2 layers, the M2 layers can be treated as nonmagnetic spacer layers. In contrast, when a nonmagnetic layer undergoes a magnetic phase transition, for example Fe/Gd multilayers, we can examine SW dynamics near the phase transition. In order to make our discussions clear and easy, we assumed no MAE and IEC for the magnetic layers. Because the calculations are rather straightforward but tedious even under these simplifications, I only show the final result. It is a 12 × 12 BCD equation given by Equation (143).

(143) $$\left|\begin{array}{cccccccccccc} m_{a} & m_{b} & & & & & & & & & & \\ m_{1} & m_{2} & m_{3} & m_{4} & & & & & & & & \\ m_{5} & m_{6} & m_{7} & m_{8} & & & & & & & & \\ & & m_{9} & m_{10} & m_{11} & m_{12} & & & & & & \\ & & m_{13} & m_{14} & m_{15} & m_{16} & & & & & & \\ & & & & m_{1} & m_{2} & m_{3} & m_{4} & & & & \\ & & & & m_{5} & m_{6} & m_{7} & m_{8} & & & & \\ & & & & & & m_{9} & m_{10} & m_{11} & m_{12} & & \\ & & & & & & m_{13} & m_{14} & m_{15} & m_{16} & & \\ & & & & & & & & m_{1} & m_{2} & m_{3} & m_{4}\\ & & & & & & & & m_{5} & m_{6} & m_{7} & m_{8}\\ & & & & & & & & & & m_{c} & m_{d} \end{array}\right|=0$$

Here, we give $m_{a}$ to $m_{d}$ elements in a matrix form for convenience’s sake.

(144) $$\begin{array}{l} \left(\begin{array}{cccccc} m_{a} & m_{b} & & & & \\ m_{1} & m_{2} & m_{3} & m_{4} & & \\ m_{5} & m_{6} & m_{7} & m_{8} & & \\ & & m_{9} & m_{10} & m_{11} & m_{12}\\ & & m_{13} & m_{14} & m_{16} & m_{16}\\ & & & & m_{c} & m_{d} \end{array}\right)\\ \;=\left(\begin{array}{cccccc} 1-\Lambda_{+}^{\left(1\right)} & \alpha^{-1}\left(1+\Lambda_{-}^{\left(1\right)}\right) & & & & \\ 1 & \alpha & -1 & -\alpha & & \\ \Lambda_{+}^{\left(1\right)} & -\alpha \Lambda_{-}^{\left(1\right)} & -\Lambda_{+}^{\left(2\right)} & \alpha \Lambda_{-}^{\left(2\right)} & & \\ & & \gamma^{-1} & \beta & -1 & -\alpha^{-1}\\ & & \gamma^{-1}\Lambda_{+}^{\left(2\right)} & -\beta \Lambda_{-}^{\left(2\right)} & -\Lambda_{+}^{\left(1\right)} & \alpha^{-1}\Lambda_{-}^{\left(1\right)}\\ & & & & \gamma \left(1+\Lambda_{+}^{\left(2\right)}\right) & \beta \left(\Lambda_{-}^{\left(2\right)}-1\right) \end{array}\right), \end{array}$$

in which $\alpha =\exp\left(-Q_{//}L_{1}\right)$, $\beta =\exp\left(-Q_{//}L_{2}\right)$, $\gamma =\exp\left(-Q_{//}\Lambda \right)$ (*Λ* = *L*_1_ + *L*_2_), and $\Lambda_{\pm }^{\left(j\right)}$ (*j* = 1, 2) have been defined in Equation (131). The rest of the determinant and matrix elements are all zero. It is clear that the elements *m*_1_ to *m*_16_ appear as a set in Equation (143). This set is the algebraic description of the M1/M2 structure. When we insert an additional set into Equation (143), we have SWs in the (M1/M2)_4_ multilayer. In this way, we can generate an arbitrary number of (M1/M2) stacking MMLs. This approach is quite intuitive and, of course, it is possible to take into account the MAEs and IECs into the above framework of the SW frequency calculations; to do so, we must solve a huge and complicated BCD equation.

Another approach to obtain the MSL SWs was developed by Camley, Rahman, and Mills [39]. For an infinite stack of periodic structures with the periodic length *Λ*, the property of translational invariance gives us Bloch’s theorem. In the present case, Bloch’s theorem requires the magnetic potential *φ*(*x*) to satisfy

(145) $$\varphi \left(x+\Lambda \right)=\exp\left(-iQ_{⊥}\Lambda \right)\varphi \left(x\right)$$

and

(146) $$\varphi \left(x\right)=\exp\left(-iQ_{⊥}x\right)u\left(x\right)$$

where *u*(*x*) is a periodic function which satisfies *u*(*x* + *Λ*) = *u*(*x*), and *Q*_⊥_ is a wave vector confined to the first Brillouin zone, 0 ≤ *Q*_⊥_ ≤π/*Λ*. For *n*th layer, we can write the magnetic potential in the *n*th layer as

(147) $$u^{\left(M1\right)}\left(x\right)=\exp\left[iQ_{⊥}\left(x+n\Lambda \right)\right]\left[A\exp\left\{-Q_{//}\left(x+n\Lambda \right)\right\}+B\exp\left\{Q_{//}\left(x+n\Lambda \right)\right\}\right]$$

for 0 ≤ *x* + *nΛ* ≤ −*L*_1_ and

(148) $$u^{\left(M2\right)}\left(x\right)=\exp\left[iQ_{⊥}\left(x+n\Lambda \right)\right]\left[C\exp\left\{-Q_{//}\left(x+n\Lambda \right)\right\}+D\exp\left\{Q_{//}\left(x+n\Lambda \right)\right\}\right]$$

for –*L*_1_ ≤ *x* + *nΛ* ≤ −*Λ*, respectively. By virtue of Bloch’s theorem, four amplitude variables, *A* to *D*, are enough for our discussions. We consider MSL SWs propagating along the y-direction and apply the magnetic boundary conditions at *x* = −*Λ* and −*Λ*−*L*_1_. Finally, we obtain a 4 × 4 BCD equation to determine the bulk SL SW frequencies.

(149) $$\left|\begin{array}{cccc} \alpha \delta & \delta & -\beta^{-1} & -\gamma \\ 1 & \alpha & -1 & -\alpha \\ \alpha \delta \Lambda_{+}^{\left(1\right)} & -\delta \Lambda_{-}^{\left(1\right)} & -\beta^{-1}\Lambda_{+}^{\left(2\right)} & \gamma \Lambda_{-}^{\left(2\right)}\\ \Lambda_{+}^{\left(1\right)} & -\alpha \Lambda_{-}^{\left(1\right)} & -\Lambda_{+}^{\left(2\right)} & \alpha \Lambda_{-}^{\left(2\right)} \end{array}\right|=0$$

in which $\alpha =\exp\left(-Q_{//}L_{1}\right),\beta =\exp\left(-Q_{//}L_{2}\right),\gamma =\exp\left(-Q_{//}\Lambda \right),$ and $\delta =\exp\left(iQ_{⊥}\Lambda \right)$. When we replace magnetic layer 2 with a nonmagnetic layer, $\Lambda_{\pm }^{\left(2\right)}=1$, Equation (149) can be reduced into

(150) $$\left[1+\Lambda_{+}\Lambda_{-}\right]\sinh Q_{//}L_{1}\sinh Q_{//}L_{2}+\left(\cosh Q_{//}L_{1}\cos Q_{//}L_{2}-\cos Q_{⊥}\Lambda \right)\left[\Lambda_{+}+\Lambda_{-}\right]=0$$

We write $\Lambda_{\pm }^{\left(1\right)}$ as $\Lambda_{\pm }$ for convenience’s sake. The bulk MSL SW frequency is found to be

(151) $${\left(\frac{\omega }{\gamma }\right)}^{2}=\frac{H\left(H+4\pi M\right)}{1+\Delta }+\frac{\Delta }{1+\Delta }\left[H^{2}+{\left(H+4\pi M\right)}^{2}\right]$$

Here, the MSL band factor Δ(*Q*_//_, *Q*_⊥_) is defined by

(152) $$\Delta \left(Q_{//},\;Q_{⊥}\right)=\frac{\sinh Q_{//}L_{1}\sinh Q_{//}L_{2}}{\cosh Q_{//}L_{1}\cos Q_{//}L_{2}-\cos Q_{⊥}\Lambda }$$

For a semi-infinite MSL, we cannot apply Bloch’s theorem because of the MSL surface, which violates the translational invariance. Taking into account the infinite MSL discussions, let us assume the magnetic potentials at the *n*th period are as follows:

(153) $$\varphi_{n}^{\left(M1\right)}\left(x\right)=\exp\left(-\varepsilon n\Lambda \right)\left[A\exp\left\{-Q_{//}\left(x+n\Lambda \right)\right\}+B\exp\left\{Q_{//}\left(x+n\Lambda \right)\right\}\right]$$

for 0 ≤ *x* + *nΛ* ≤ −*L*_1_ and

(154) $$\varphi_{n}^{\left(M2\right)}\left(x\right)=\exp\left(-\varepsilon n\Lambda \right)\left[C\exp\left\{-Q_{//}\left(x+n\Lambda \right)\right\}+D\exp\left\{Q_{//}\left(x+n\Lambda \right)\right\}\right]$$

for –*L*_1_ ≤ *x* + *nΛ* ≤ −*Λ*. Here, *ε* is a positive attenuation parameter of MSL surface mode. We can eliminate *C* and *D* from the boundary conditions and obtain another homogeneous equation on *A* and *B* given as

(155) $$\begin{array}{l} \left(\Lambda_{+}^{\left(2\right)}-\Lambda_{+}^{\left(1\right)}\right)\left[1-\exp\left(-Q_{//}L_{1}\right)\exp\left(-\varepsilon \Lambda \right)\exp\left(Q_{//}L_{2}\right)\right]A\\ \;\;\;\;+\left(\Lambda_{+}^{\left(2\right)}+\Lambda_{-}^{\left(1\right)}\right)\left[\exp\left(-Q_{//}L_{1}\right)-\exp\left(-\varepsilon \Lambda \right)\exp\left(Q_{//}L_{2}\right)\right]B=0, \end{array}$$

and

(156) $$\begin{array}{l} \left(\Lambda_{+}^{\left(1\right)}+\Lambda_{-}^{\left(2\right)}\right)\left[\exp\left(-Q_{//}L_{1}\right)\exp\left(-\varepsilon \Lambda \right)-\exp\left(Q_{//}L_{2}\right)\right]A\\ \;\;\;\;+\left(\Lambda_{-}^{\left(2\right)}-\Lambda_{-}^{\left(1\right)}\right)\left[\exp\left(-\varepsilon \Lambda \right)-\exp\left(Q_{//}L_{2}\right)\exp\left(-Q_{//}L_{1}\right)\right]B=0. \end{array}$$

From Equations (155) and (156), the attenuation parameter *ε* is required to satisfy

(157) $$\begin{array}{l} 2\left[\Lambda_{+}^{\left(2\right)}\Lambda_{-}^{\left(2\right)}+\Lambda_{+}^{\left(1\right)}\Lambda_{-}^{\left(1\right)}\right]\sinh\left(Q_{//}L_{2}\right)\sinh\left(Q_{//}L_{1}\right)\\ \;\;+\left(\Lambda_{+}^{\left(2\right)}\Lambda_{-}^{\left(1\right)}+\Lambda_{+}^{\left(1\right)}\Lambda_{-}^{\left(2\right)}\right)\left[\cosh\left\{Q_{//}\left(L_{2}-L_{1}\right)\right\}-\cosh\left(\varepsilon \Lambda \right)\right]\\ \;\;\;\;+\left(\Lambda_{+}^{\left(1\right)}\Lambda_{+}^{\left(2\right)}+\Lambda_{-}^{\left(1\right)}\Lambda_{-}^{\left(2\right)}\right)\left[\cosh\left\{Q_{//}\left(L_{2}+L_{1}\right)\right\}-\cosh\left(\varepsilon \Lambda \right)\right]=0. \end{array}$$

Finally, we must include the boundary conditions at the surface. By eliminating the magnetic potential outside MSL, we obtain

(158) $$\alpha \left[\Lambda_{+}^{\left(1\right)}-1\right]A-\left[\Lambda_{-}^{\left(1\right)}+1\right]B=0$$

The dispersion relation of the MSL surface SWs is given as

(159) $$\left|\begin{array}{cc} \left(\Lambda_{+}^{\left(2\right)}-\Lambda_{+}^{\left(1\right)}\right)\left(1-\alpha \beta^{-1}\kappa \right) & \left(\Lambda_{-}^{\left(1\right)}+\Lambda_{+}^{\left(2\right)}\right)\left(\alpha -\beta^{-1}\kappa \right)\\ \alpha \left(\Lambda_{+}^{\left(1\right)}-1\right) & -\left(\Lambda_{-}^{\left(1\right)}+1\right) \end{array}\right|=0.$$

Here, we defined κ=exp(−εΛ). Equation (159) gives the dispersion relation of the surface modes.

(160) $$\left(\Lambda_{-}^{\left(1\right)}+1\right)\left(\Lambda_{+}^{\left(2\right)}-\Lambda_{+}^{\left(1\right)}\right)\left(1-\alpha \beta^{-1}\kappa \right)+\left(\Lambda_{+}^{\left(1\right)}-1\right)\left(\Lambda_{-}^{\left(1\right)}+\Lambda_{+}^{\left(2\right)}\right)\left(\alpha^{2}-\alpha \beta^{-1}\kappa \right)=0$$

Now, we replace magnetic layer 2 by a nonmagnetic layer again and write $\Lambda_{\pm }^{\left(1\right)}$ as $\Lambda_{\pm }$ for convenience’s sake. Equation (160) gives

(161) $$\left(\Lambda_{-}+1\right)\left(1-\Lambda_{+}\right)\left(1-\alpha \beta^{-1}\kappa \right)+\left(\Lambda_{+}-1\right)\left(\Lambda_{-}+1\right)\left(\alpha^{2}-\alpha \beta^{-1}\kappa \right)=0\;\left(\Lambda_{+}-1\right)\left(\Lambda_{-}+1\right)\sinh Q_{//}L_{1}=0$$

Equation (161) gives us three solutions: $\Lambda_{+}-1=0$, $\Lambda_{-}+1=0$, and $\sinh Q_{//}L_{1}=0$. However, all three of these solutions cannot be the eligible surface SWs. Readers should note that $\Lambda_{\pm }$ in this review corresponds to $\Lambda_{\mp }$ in the original paper. For example, we obtained *A*≠0 and *B* = 0 for the $\Lambda_{+}-1=0$ solution from Equation (158). Equation (155) is automatically satisfied, and Equation (156) gives $\exp\left(-Q_{//}L_{1}\right)\exp\left(-\varepsilon \Lambda \right)-\exp\left(Q_{//}L_{2}\right)=0$. Because this equation gives a negative *ε*, the $\Lambda_{+}-1=0$ solution cannot be an eligible surface mode. On the other hand, the $\Lambda_{-}+1=0$ solution gives a positive ε for *L*_1_ > *L*_2_ and thus can be an eligible surface mode. In fact, Grimsditch et al. observed BLS from the surface mode only for an MSL with a magnetic layer thicker than the nonmagnetic spacer layer [91]. The surface mode frequency was found to be [39]

(162) $$\frac{\omega }{\gamma }=H+2\pi M$$

This frequency is exactly the same as the DE mode frequency given by Equation (52) for a semi-infinite magnet. We can readily reject the $\sinh Q_{//}L_{1}=0$ solution for SWs propagating along the *y* direction because of the real quantity *Q*_//_*L*_1_≠0. This mode can exist within the bulk SW band given by Equation (41).

Because a large number of reports on BLS from MMLs and MSLs have been already published, I have not enough space to mention them. I recommend that the reader refer to the references in this review and the most recent publications. 

**Figure 25:**
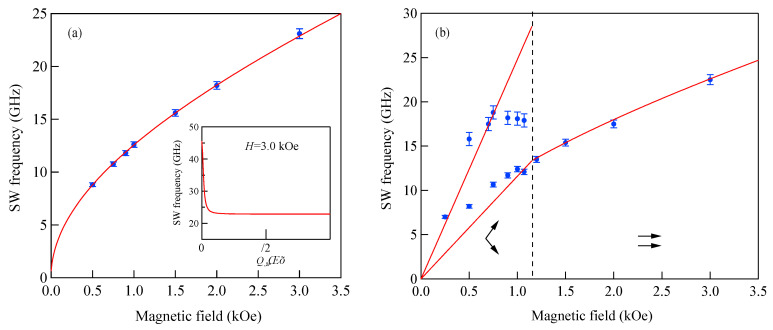
(**a**) SW frequencies as a function of the magnetic field for the [Fe(30 Å)/Cr(21 Å)] MML. The solid lines show the calculated SW frequencies by using Equation (151) with *Q*_//_ = 0.67 × 10^5^ cm^−1^, *γ*/2*π* = 2.88 GHz/kOe, and 4*πM* = 18.0 kG. The inset shows the SW frequencies as a function of the *Q*_//_*Λ* parameter. (**b**) SW frequencies as a function of the magnetic field for [Fe(30 Å)/Cr(13 Å)] MML. The solid lines were calculated by using Equations (162)–(164) below *H* = 1.15 kOe and by Equation (151) above *H* = 1.15 kOe [92].

We examined the SWs in [Fe (30 Å)/Cr(*x* Å)] (*x* = 8–60 Å) MMLs prepared at IMR by rf-sputtering on quartz substrate [92]. The total thickness of each MML was fixed at ~1000 Å. BLS spectra were excited by the 4880 Å line from an Ar^+^ laser with a power of ~80 mW with *ϑ* = 15^°^ (*Q*_//_ = 0.67 × 10^5^ cm^−1^). Figure 25a shows the SW frequencies as a function of the magnetic field for Fe (30 Å)/Cr(21 Å) MML. The Fe (30 Å)/Cr(21 Å) MML shows a typical ferromagnetic loop. 

The solid line in Figure 25a was calculated by using Equations (151) and (152) with *g* = 2.06 (*γ*/2*π* = 2.88 GHz/kOe), 4*πM* = 18.0 kG, *L*_1_ = 30 Å, and *L*_2_ = 21 Å. The calculated line shows excellent agreement with the observed frequencies. The inset shows the SW frequencies at *H* = 3.0 kOe as a function of the $Q_{⊥}\Lambda$ parameter in Equation (152). It is clear that the SW band frequency became independent of the *Q*_⊥_*Λ* parameter above *Q*_⊥_*Λ*~*π*/10. It means that BLS observed the lower bound of the bulk SW band. Because the magnetic layer thickness of the tested MML was thicker than the nonmagnetic Cr spacer layer, we expected to observe scattering from the surface SW peak at the frequency given by Equation (162). The surface SW frequency was expected to be ~27 GHz at *H* = 0.5 kOe and ~35 GHz at *H* = 3.0 kOe. Because our FPI mirror spacing was set to 5 mm in this study, the surface SW peak was unfortunately masked by the intense ghost peak from the adjacent interference order. Figure 25b shows the SW frequencies as a function of the magnetic field for Fe (30 Å)/Cr(13 Å) MML. I performed an additional measurement at *H* = 0.9 kOe and added the result in Figure 25b. The in-plane hysteresis loop from the Fe (30 Å)/Cr(13 Å) MML indicates the antiferromagnetic structure of the magnetizations between adjacent Fe layers under zero magnetic field. The loop indicates the in-plane canted structure below ~1.2 kOe and the ferromagnetic aligned structure above ~1.2 kOe. We can define the transition of the magnetic field *H*_C_ from the canted state to the ferromagnetic state. We observed an SW doublet on both frequency sides below *H* = 1.1 kOe. To the contrary, we observed an SW singlet above 1.2 kOe. When we take account the hysteresis loop result, the SW doublet is clearly related to the canted magnetization state. Generally speaking, the magnetic unit cell in the canted state is essentially the same as in the antiferromagnetic state and is double the unit cell in the ferromagnetic state. Therefore, we can expect a doublet of SW peaks in the canted state and a singlet bulk SW peak in the ferromagnetic state. Nörtemann et al. calculated SW frequencies of the dipole modes in an exchange-coupled MML with a canted ground state in terms of the effective-medium theory [90]. The most striking feature of their results is the mode crossing between the upper bulk SW band and the surface mode around ~*H*_C_/2. For a semi-infinite canted stack without the MAE and the SWs propagating perpendicular to the net magnetization, the SW frequencies, except the mode crossing region around ~*H*_C_/2, in the canted state are given as

(163) $$\omega_{1}/\gamma ={\left(H\right)}^{1/2}{\left(H+4\pi M_{\mathrm{SL}}\cos\alpha \right)}^{1/2}$$

and

(164) $$\omega_{2}/\gamma =H+2\pi M_{\mathrm{SL}}\cos\alpha$$

The equilibrium canting angle *α* (note that 2*α* is the true canting angle between the adjacent magnetizations) is given as

(165) $$\cos\alpha =\frac{H}{2\left(H_{E}/N\right)}=\frac{H}{H_{C}}$$

Here, *H*_E_ is the interlayer exchange field and *N* is the number of atomic layers in the MML. The solid lines in Figure 25b were calculated by Equations (163)–(165) below *H*_C_ and by Equation (151) above *H*_C_ with the magnetic constants *g* = 2.06 (*γ*/2*π* = 2.88 GHz/kOe), 4*πM* = 17.5 kG, *H*_C_ =1.15 kOe, *L*_1_ = 30 Å, and *L*_2_ = 13 Å. Agreement in the canted state below *H*_C_ was not satisfactory. For a possible reason, we consider that our MML consists of only 11~12 canted units, which is given by 2[Fe (30 Å)/Cr(13 Å)]. On the other hand, the effective-medium theory assumes a semi-infinite or a large number of canted stacks with ideal sharp interfaces.

So far, our discussion on IEC has been based on the assumption that the asymptotic limit is applicable. The term “asymptotic limit” means that the coupling is independent of the ferromagnetic layer thickness, and that the interlayer thickness is large as compared to the Fermi wavelength *λ*_F_ = 2*π*/*k*_F_, which is typically either ~5 Å or a few monolayers (MLs) in many metals [93]. For thicknesses below *λ*_F_, IEC cannot be described by our previous theoretical framework, and we should apply more a fundamental numerical method, for example, ab initio, by the self-consistent full-potential linearized augmented-plane-wave (FLAPW) method. Fine-layered [Fe (*n* ML)/Au (*n* ML)]_m_ SLs with *n* = 1 to 5, for which we use the abbreviation (*n*)_m_, were prepared by means of the MBE on MgO(001) substrates at IMR, Tohoku University. The total numbers of Fe and Au atomic planes were kept constant. We use the term “fine-layered” SLs (FLSLs) for SLs with layer thicknesses comparable or smaller than the Fermi wavelength. The Fe(1 ML)/Au(1 ML) FLSL corresponds to the ordered alloys with the tetragonal *L*1_0_ structure, which exists in the equilibrium phase diagram for Fe_1_Pt_1_ alloy but not for the Fe_1_Au_1_ alloy.

**Figure 26:**
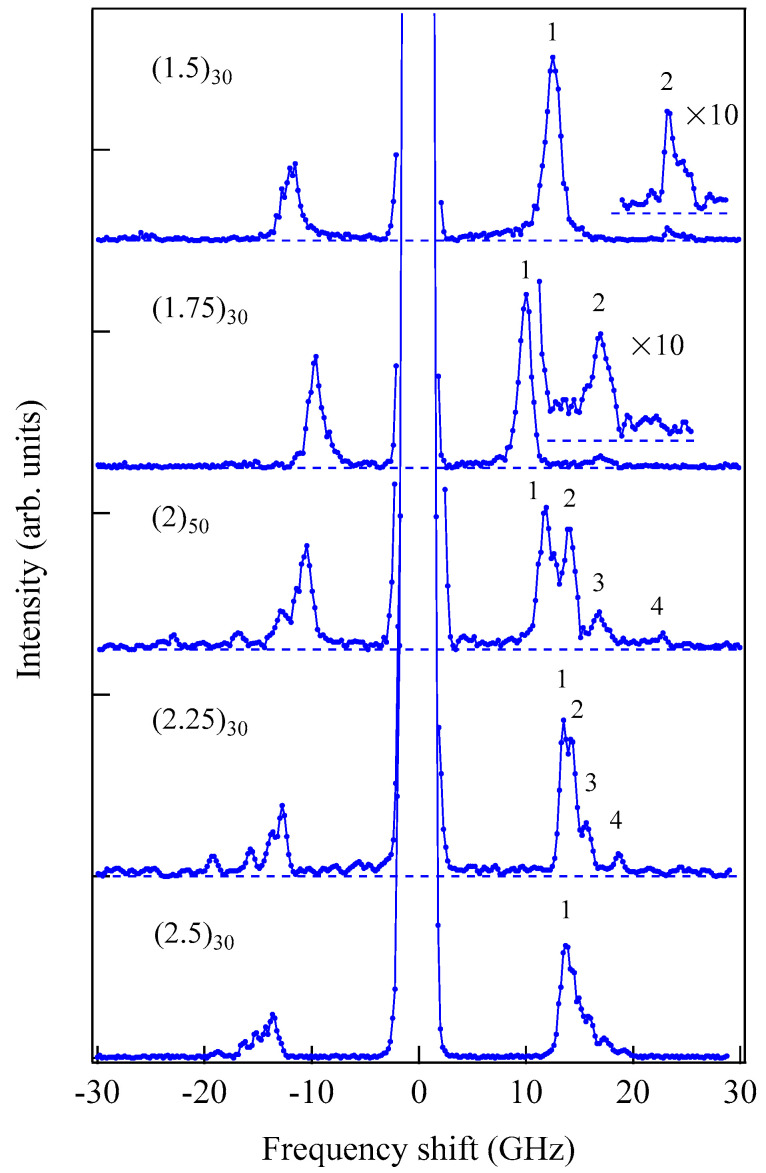
BLS spectra observed from 2 ± *δ* ML FLSLs (*δ* = 0.25 and 0.5) at *H* = 3.0 kOe. The labels 1 to 4 stand for the corresponding SSW number [95].

BLS spectra at 300 K were excited by the *p*-polarized 5320 Å/150 mW line from a DPSS laser, and a cross-polarized analyzer was inserted in front of a tandem FPI to eliminate scattering from SAWs [94,95]. Magnetic fields of up to 7 kOe were applied along the crystallographic $\left(1\bar{1}0\right)$ direction, and the SWs propagating along the (110) direction were examined. Typical spectrum accumulation time was around 2h. Figure 26 displays BLS spectra observed from (*n*)_m_ FLSLs in an external magnetic field of 3.0 kOe. 

**Figure 27:**
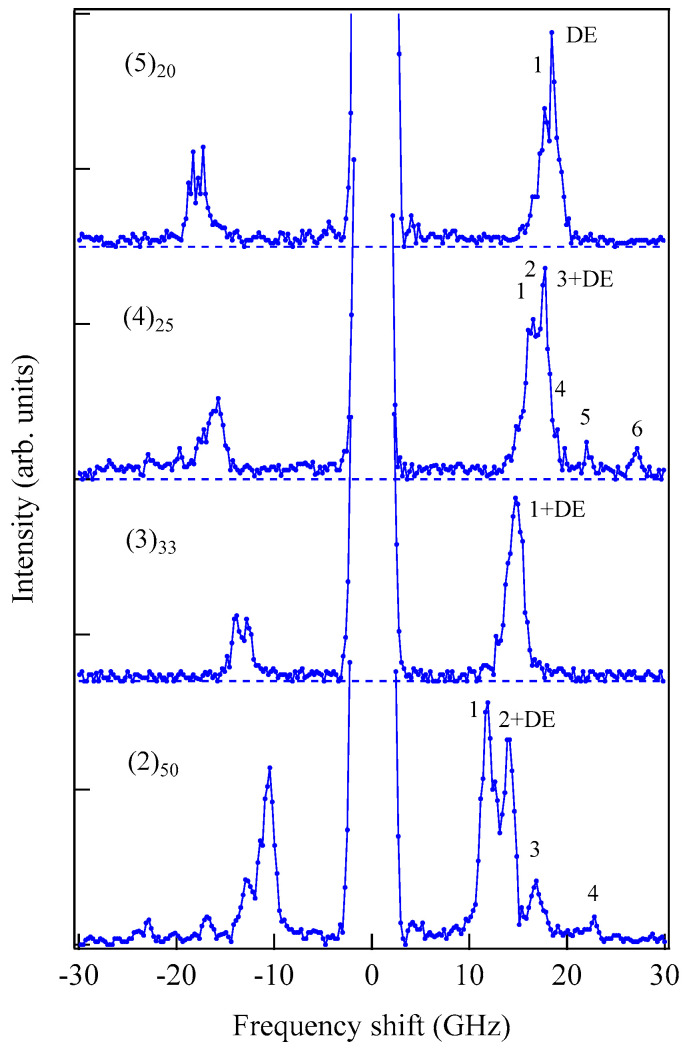
A comparison of BLS spectra observed from the integer-type FLSLs. The experimental conditions were the same as the ones given in Figure 25. The labels 1 to 5 and DE stand for the SSW modes and the DE mode, respectively [95].

Here, the symbol of *n* = 2 ± *δ* indicates an average ML, and *δ* = 0.25 and 0.5. The total thickness *L*_SL_ of the (1.5)_30_ FLSL is 145 Å, and it is 269 Å for the (2.5)_30_ FLSL. The SSW structure can be clearly seen, and the labels 1 to 4 on each spectrum stand for the corresponding SSW mode number. Figure 27 shows a comparison of the BLS spectra observed from the integer-type FLSLs. 

The even integer-type FLSLs exhibited clear SSW structures, but not the odd integer-type FLSLs. Furthermore, the noninteger-type spectra shown in Figure 26 can be smoothly connected between the (2)_50_ and (3)_33_ spectra. These observations indicate that the interactions leading to the occurrence of SSW systematically changed as a function of the ML number *n*. In order to analyze these results, we regarded the FLSLs as diluted magnetic Fe/Au alloy films with uniaxial MAEs perpendicular to the film plane and anisotropic exchange couplings. The magnetization of the alloy was assumed to be given by *M* = *M*_Fe_*d*_Fe_/(*d*_Fe_ + *d*_Au_) = *M*_Fe_*d*_Fe_/*Λ*_SL_. Here, *d* is the thickness of each layer (*d*_Fe_ = 3.00 Å and *d*_Au_ = 4.27 Å) and *M*_Fe_ = 2.65 ± 0.2 *μ*_B_ per Fe atom. The *n*th SSW frequency is given as

(166) $$\Delta \nu \left(n\right)=\frac{\gamma }{2\pi }{\left(H+H_{ex}\left(n\right)\right)}^{1/2}{\left(H+H_{ex}\left(n\right)+4\pi M_{eff}\right)}^{1/2}$$

and

(167) $$H_{ex}\left(n\right)=D_{//}Q_{//}^{2}+D_{⊥}{\left(\frac{n\pi }{L_{SL}}\right)}^{2}≅D_{⊥}{\left(\frac{n\pi }{L_{SL}}\right)}^{2}$$

**Figure 28:**
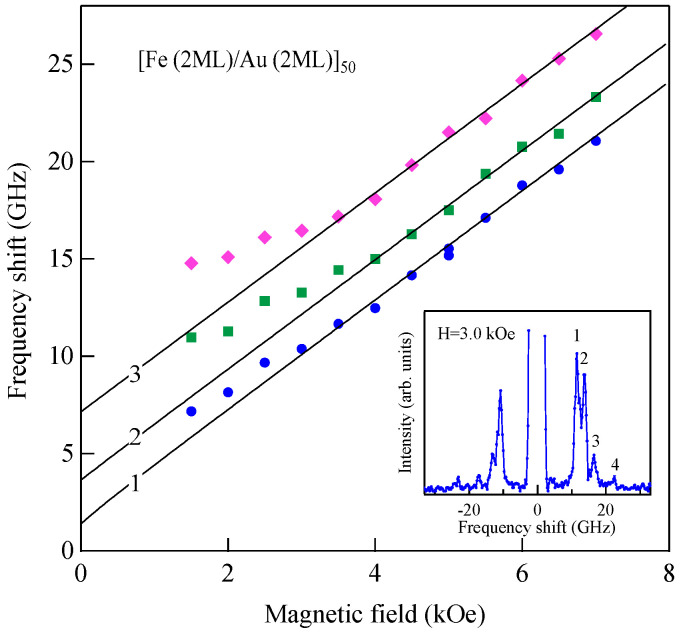
Magnetic field dependence of the SW frequencies for the (2)_50_ FLSL. The inset shows the BLS spectrum from the (2)_50_ FLSL at *H* = 3.0 kOe. The solid lines were calculated for the SSWs by using Equations (166) and (167) with the magnetic constants *γ*/2*π* = 2.8 GHz/kOe (*g* = 2.0), 4*πM*_eff_ = 0.75 kG, and *D*_⊥_ = 2.4 × 10^−10^ Oe·cm^2^ [94].

Here, 4*πM*_eff_ has been already given by Equation (93), and *L*_SL_ is the total thickness of the FLSL, which was found to be 364 Å with XRD measurement. Figure 28 shows the SSW frequencies as a function of the external magnetic field for the (2)_50_ FLSL. 

**Figure 29:**
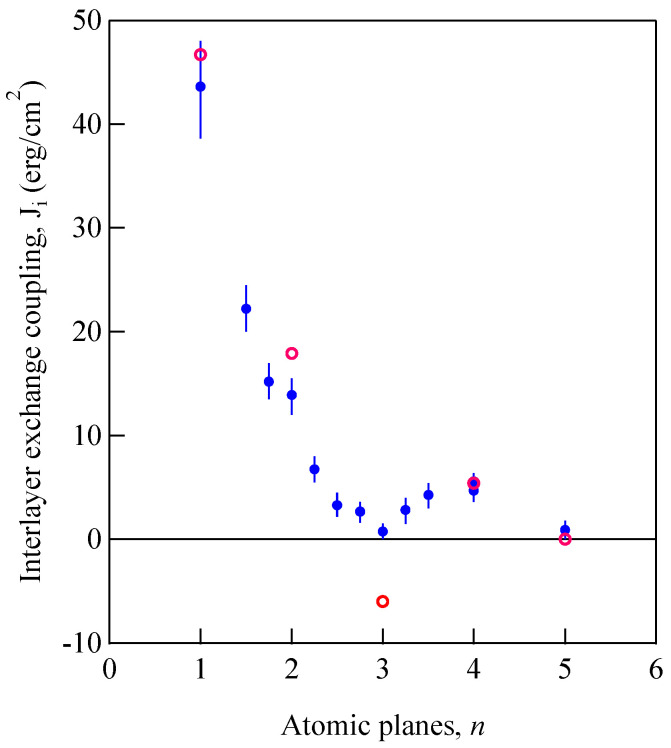
The IEC constant *J*i as a function of *n*. The solid circles (●) are the BLS results, and the open circles (○) are the ab initio results [95].

It can be readily seen that the magnetic moment was not fully saturated below *H* < 4 kOe. Because we confirmed that the DE mode was superimposed on the *n* = 2 SSW peak through the surface dispersion examination by changing the scattering angle ϑ in Equation (1) from 15° (*Q*_//_ = 0.61 × 10^5^ cm^−1^) to 45° (*Q*_//_ = 1.67 × 10^5^ cm^−1^) and that the DE mode does not contribute to the information on the exchange, the DE mode was therefore not included in the present analysis. We obtained a set of the magnetic constants given by *γ*/2*π* = 2.8 GHz/kOe (*g* = 2.0), 4*πM*_eff_ = 0.75 kG, and *D*_⊥_ = 2.4 × 10^−10^ Oe·cm^2^. Figure 29 shows the IEC constant *J*i as a function of the atomic plane *n*. 

We observed a pair of DE peaks for a (1)_30_ FLSL but observed the SSW structure up to *n* = 3 SSW for a (1)_100_ FLSL. The *J*_i_ constant was evaluated by using *J*i = 2*MD*_⊥_/*a*_Fe_, in which *a*_Fe_ = 2.87 Å is the lattice constant of Fe. The open symbols stand for the ab initio results. Overall agreement between the BLS and ab initio results was fairly good, except for *n* = 3. The ab initio calculation predicted an antiferromagnetic ground state. We consider the discrepancy between the BLS and ab initio results for *n* = 3 stemmed from interface roughness. Because the IECs for *n* = 2 and 4 were ferromagnetic, the antiferromagnetic IEC for the ideal *n* = 3 FLSL may have been smeared for long-wavelength SWs detected with the BLS technique. For pure Fe films in full contact, the *J*_i_ value was expected to be 140 erg/cm^2^. Hence, the present value of *J*_i_~44 erg/cm^2^ for the (1)_100_ FLSL seems to be reasonable. According to the ab initio calculations, *d* electrons from the Fe atom were almost isolated even by 1 ML of the Au layer. The IEC was transmitted by itinerant *sp* electrons via second-order processes. 

**Figure 30:**
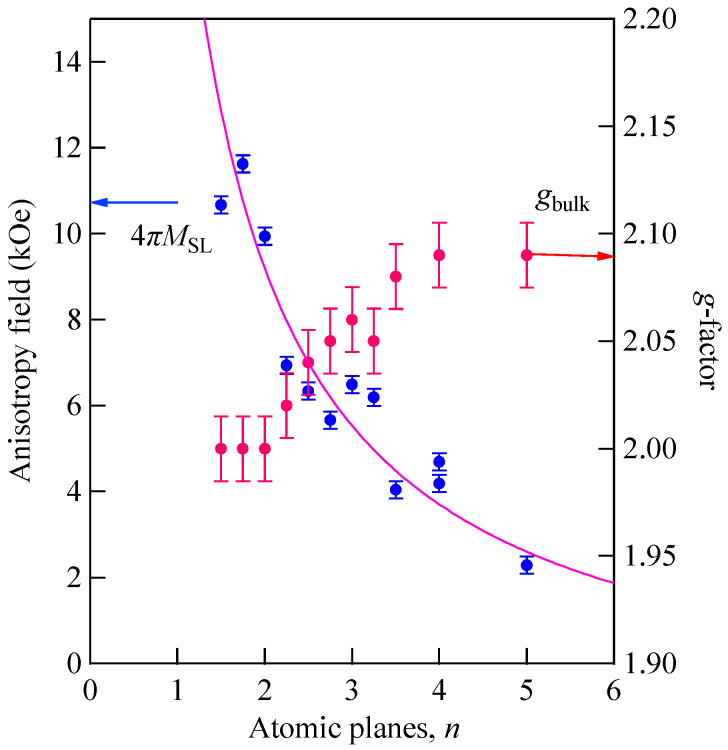
*g*-factor (●) and anisotropy field (●) as a function of *n*. The solid line shows the perpendicular anisotropy field *H*_A_(*n*) = 22/*n* − 1.8 (kOe).

Figure 30 shows the perpendicular anisotropy field and the *g*-factor as a function of the atomic layer. 

The solid line represents the 1/*n* dependence given by *H*_A_(*n*) = 22/*n* − 1.8 (kOe). The 1/*n* dependence was expected from the interface out-of-plane anisotropy, as we have already discussed for the Fe wedge sandwiched by the Au layers. The anisotropy field *H*_A_(*n*) is defined as

(168) $$H_{A}\left(n\right)=\frac{8\pi }{4\pi M_{\mathrm{SL}}}\left(K_{⊥}+\frac{2k_{⊥}^{\left(s\right)}}{\Lambda_{\mathrm{SL}}n}\times 10^{8}\right)$$

When we ignore the bulk term in Equation (168) and use 4*πM*_SL_ = 10.7 kG, we obtain a value of $k_{⊥}^{\left(s\right)}\approx 0.34$ erg/cm^2^. This value is in reasonable agreement with the values determined from the Fe wedge (see Figure 20) [79]. It is also clear that 4*πM*_eff_ = 4*πM*_SL_ − *H*_A_(*n*) changes its sign from positive to negative around *n*~2. As we have already discussed for the Fe wedge, it means the in-plane magnetization state changes into the perpendicular magnetization state under zero magnetic field. The present Fe/Au FLSL and the Fe wedge sandwiched by the Au layers gave consistent results on the in-plane and out-of-plane transitions of the zero field magnetization state. Another interesting observation is the rather rapid change of the g-factor from the bulk value of 2.09 above *n* ≥ 4 to the free electron value of 2.00 below *n* ≤ 2. We also found that the *g*-factor of the Fe wedge at 2.6 Å was very close to 2.00. 

### 5.5. Nanogranular Films

**Figure 31:**
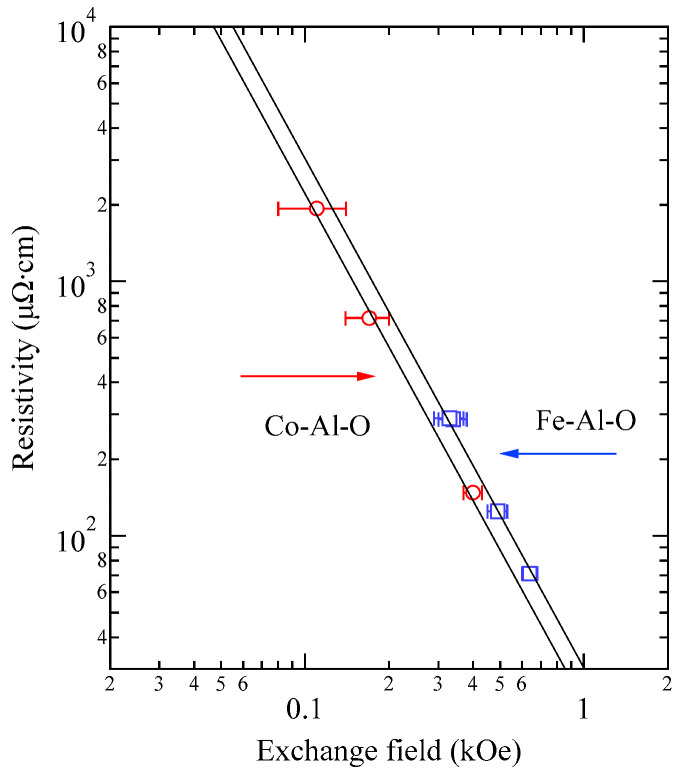
A log–log plot between *ρ* and *H*_E_ for Co-Al-O (○) and Fe-Al-O (□) granular films. The solid lines give the power law given by *ρ* = *a*(*H*_ex_)^−2^. We obtained *a*_Fe_ = 30.3 μΩ⋅cm⋅(kOe)^2^ for the Fe-Al-O films and *a*_Co_ = 22.1 μΩ⋅cm⋅(kOe)^2^ for the Co-Al-O films [54].

Transition metal (TM = Fe, Co)-based granular films are higher-potential materials for various magnetic applications, for example, high-density longitudinal magnetic recording media (CoPt-SiO_2_), high-frequency micromagnetic cores (CoFeB)-(SiO_2_), GMR sensors (Co-Al-O), and so on [55]. Among these granular materials, TM-Al-O granular films are interesting materials for both basic magnetic research and for technological applications. Over the last twenty years, we have performed systematic studies on the magnetic and transport properties of TM-Al-O nanogranular films with the research groups of IMRAM and IMR, Tohoku University, and RIEMM, Sendai [54,55,96,97,98,99,100,101]. Readers who are interested in our results on transport and magnetization properties can refer to our references; here, I concentrate on our BLS results. It is well-known that the magnetic properties of TM-Al-O (TM = Fe, Co) granular films strongly depend on the TM composition. For example, for the Co composition *x*(Co) above ~70 at. %, Co-Al-O films are in a ferromagnetic (FM) ground state with a lower coercive field of *H*_C_ > 10 Oe. On the other hand, for *x*(Co) = 60~70 at %, TM-Al-O films exhibit reasonable soft magnetic properties with *H*_C_ < 10 Oe, whereas Co-Al-O films with *x*(Co) = 40~60 at % are in a superparamagnetic (SPM) state. I have already shown a BLS spectrum obtained from FM Co-Al-O films prepared at RIEMM in Figure 3 and the SW frequencies as a function of the magnetic field for a FM Fe_64_-Al_19_-O_17_ film prepared at RIEMM in Figure 5 [54]. The TM-Al-O nanogranular films consist of TM crystalline particles of up to several nm in diameter. The TM particles are surrounded by a nonmagnetic Al-O grain boundary of ~1 nm in thickness. In spite of the granular structure, we could observe well-defined SW spectra as shown in Figure 3, and we found that the SW frequencies, as a function of the magnetic field, are fully described by using Equations (49) and (50) as developed for uniform FM films (see Figure 5). As already shown in Figure 5, the small exchange field term *H*_ex_ = *DQ*^2^ = 0.32 kOe is important to reproduce the observed bulk SW frequency. As I have already mentioned, we cannot determine the SW stiffness constant *D* from the exchange field term. We found the resistivity ρ of the FM TM-Al-O granular films obeys the *T*^2^ law in a wider temperature range below 200 K. Although the *T*^2^ law can be expected from magnon scattering of conduction electrons at low temperatures, it has not been fully confirmed yet for FM metals, probably due to the much larger *T*^5^ term by phonon scattering. Because magnon resistivity also depends on the exchange stiffness constant *D*, and the magnon *T*^2^ term can be replaced by (*T*/*D*)^2^, we can therefore expect the inverse-square law *ρ*∝(*H*_ex_)^−2^. Figure 31 shows a log *ρ* vs log *H*_ex_ plot. We obtained *ρ*_Fe_ = 30.3(*H*_ex_)^−2^ μΩ·cm and *ρ*_Co_ = 22.1(*H*_ex_)^−2^ μΩ·cm, respectively. Here, *H*_ex_ is in the kOe unit [54].

**Figure 32:**
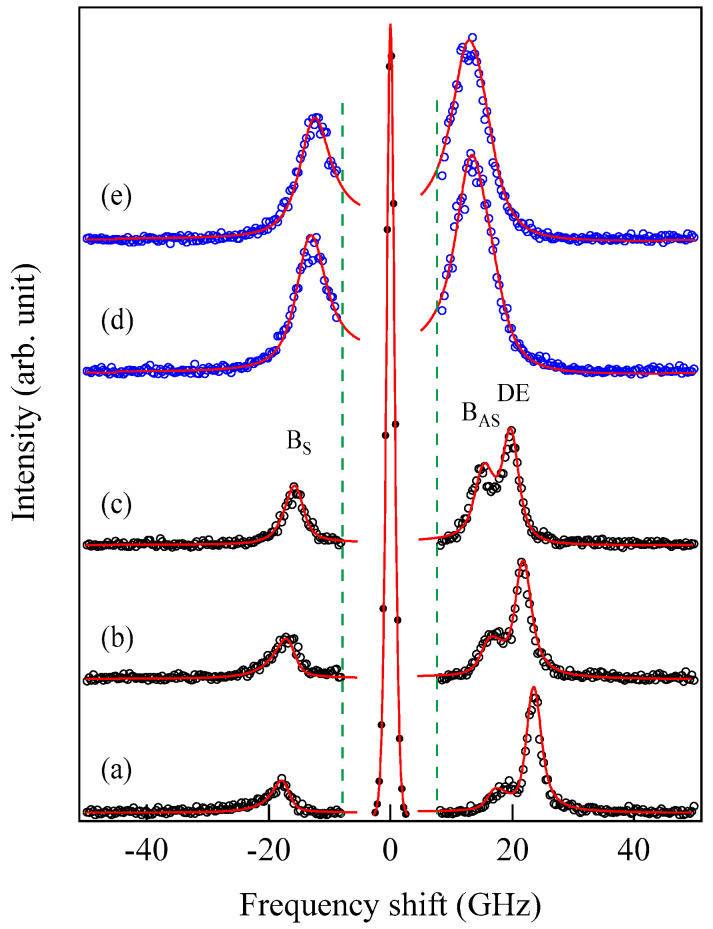
BLS spectrum from the SPM and FM Co-Al-O films at room temperature under an external magnetic field of 2.0 kOe. Here, (**a**) *x*(Co) = 72.4, (**b**) *x*(Co) = 67.5, (**c**) *x*(Co) = 63.1, (**d**) *x*(Co) = 50.3, and (**e**) *x*(Co) = 49.9, respectively. The baseline for each spectrum was properly shifted for clarity’s sake. The solid lines give the numerical fits by the CM theory. The vertical broken lines indicate the frequency area in which the AOMs were activated to protect the PMT. An intensity-attenuated elastic Rayleigh peak around zero frequency is also displayed. This peak gives the instrumental function [101].

Hereafter, I would like to concentrate on the Co-Al-O granular films prepared at RIEMM. Figure 32 shows a series of cross-polarized BLS spectra observed at room temperature from the FM and SPM Co-Al-O granular films in a magnetic field of *H* = 2.0 kOe [101]. 

**Figure 33:**
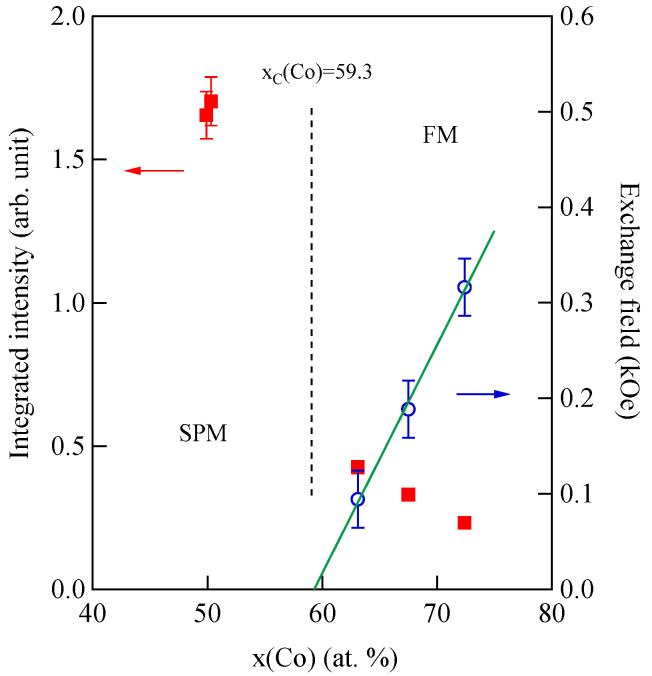
Integrated intensity (solid square) of the Stokes peak of BLS spectra shown in Figure 32 and the exchange field *H*_ex_ (○) calculated from the inverse-square law for the FM films as a function of *x*(Co). The green solid line gives *H*_ex_ = 23.9 × 10^−3^ (*x*(Co) − 59.3) kOe obtained from a least-squares fitting [101]. The broken line indicates the FM/SPM boundary given by *H*_ex_ = 0.

These spectra were excited by the *p*-polarized 4730 Å line from a DPSS laser with an output power of 30 mW and accumulated over 5 h to improve the S/N ratio. The peak intensity of each spectrum was normalized by the total spectrum accumulation times. By virtue of the *p* → *s* polarization selection, the SAW scattering was completely suppressed. The solid squares in Figure 33 show the integrated intensity of the Stokes peak. The integrated intensity of the bulk SW in the FM state suddenly jumped in the FIM state. In order to determine the FM–SPM boundary, the exchange field *H*_ex_ proved a good guide. The inverse-square relation between the exchange field *H*_ex_ (kOe) and the resistivity *ρ* (μΩ⋅cm) gives us *H*_ex_ = (22.1/*ρ*)^1/2^ kOe for FM Co-Al-O films [54]. We calculated the exchange field *H*_ex_ using our resistivity data *ρ*, and we show the calculated exchange fields by the open circles in Figure 33. 

We also expected a linear relation *H*_ex_∝ *x* − *x*_C_, in which *x*_C_ is the FM–SPM boundary concentration. The solid line in Figure 33 displays *H*_ex_ = 23.9 × 10^−3^(*x*(Co) − 59.3) (kOe) determined using the least-squares method. With this result, we determined the SPM-FM boundary in the Co-Al-O nanogranular system was located at *x*_C_(Co) = 59.3 ± 1.3. The SPM–FM boundary can be characterized as a Co atomic concentration at which the exchange stiffness constant *D* vanishes. Therefore, we should take into account both the exchange and dipole coupling for the FM films. On the other hand, we can ignore the exchange coupling in the BLS spectrum calculation for the FIM state of the SPM films. 

There is a distinct difference between the bottom three FM spectra, (a) through (c), and the top two SPM spectra, (d) and (e). The FM spectrum exhibited a characteristic dual peak structure on the positive frequency anti-Stokes (SW annihilation process) side and a single peak on the negative frequency Stokes (SW creation process) side under the present experimental conditions. These spectral features are typical for a SW spectrum from a thick FM film. An opaque magnetic film with a thickness of ~*λ*/2 can be treated as a semi-infinite magnet in the BLS experiment, as I have already mentioned. Here, the labels DE and B refer to the DE and bulk SW peaks, and the subscript S and AS refer to the Stokes and anti-Stokes processes. Note that the DE peak height is higher than that of the FM bulk peaks in (a) through (c). On the other hand, it is quite interesting to note that only a broad but intense peak appears on both frequency sides in the SPM spectra. It seems to be a general feature of BLS spectra from the SPM state. In fact, broad BLS peaks have been also observed in CoPt-SiO_2_ granular films [102] and (SiO_2_)_100−x_Co_x_ granular films [103]. Hereafter, for convenience’s sake, let us define the magnetization-induced state under an external magnetic field in an SPM film as the field-induced magnetization (FIM) state. These FIM peak frequencies were quite sensitive to the external magnetic field *H*. The peak frequencies increased with the increasing magnetic field. It is also an interesting observation that the peak frequency in the anti-Stokes side was about 1 GHz higher at most than the peak frequency in the Stokes side. Damon and Eshbach discussed a dipole-coupled ferromagnetic slab and obtained a nonreciprocal DE mode in addition to the bulk SW band [24]. This means that we can expect a singlet-doublet SW structure for a BLS spectrum from an FIM slab. The peak frequency difference of ~1 GHz between the Stokes and anti-Stokes sides is probably due to the DE mode, which only appears in the anti-Stokes side in our scattering geometry (see Figure 32a–c). In order to separate the bulk and DE peaks and determine the peak frequency, peak width, and intensity from the observed broad peak, numerical spectrum analysis is strongly required. For quantitative analyses of BLS spectra beyond the peak frequency discussions we have performed so far, we employed the CM theory, which has been developed for semi-infinite magnets, by taking into account both the dipole and exchange couplings [29]. The CM theory can fully reproduce the singlet-doublet SW structure for a BLS spectrum from a ferromagnetic slab, as shown in Figure 32a–c. According to the CM theory, the SW response function *S*(*Q*_//_, *ω*) is given by rather complicated formulae:

(169) S(Q//,ω)∝n(ω, T) cosϑ |γs(ϑ)|2(ω0c)4Im∫0∞dz∫0∞dz′exp[iΔk⊥z−iΔk⊥*z′]  ×{Rzzχzz(z,z′|ω)+Rxxχxx(z,z′|ω)+Rxzχxz(z,z′|ω)+Rzxχzx(z,z′|ω)}.

**Figure 34:**
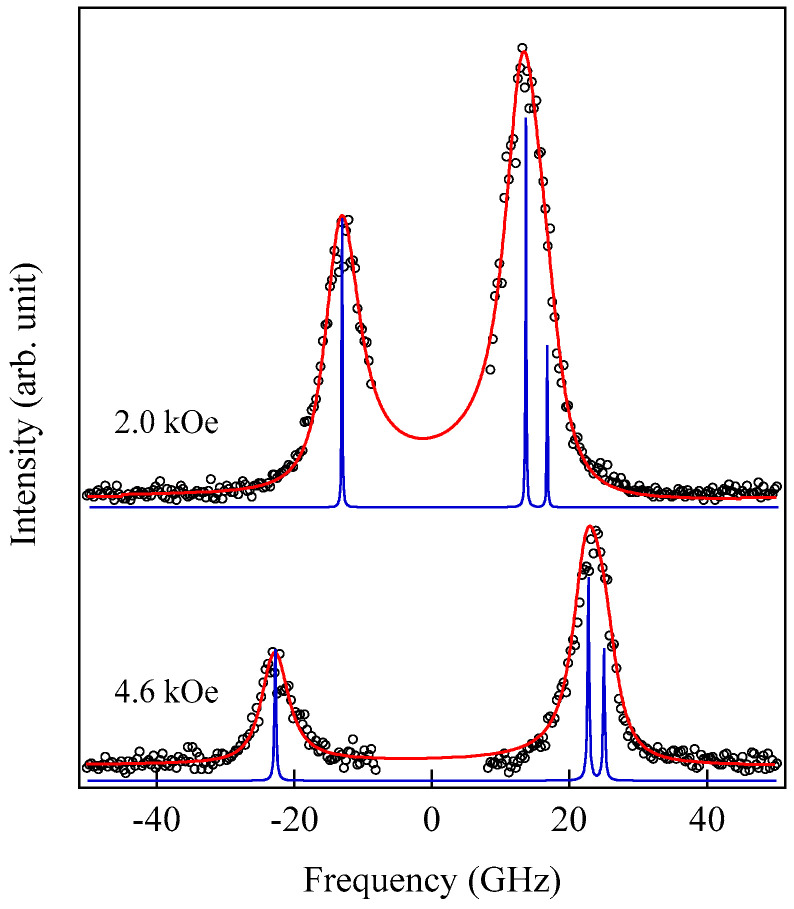
Comparison of BLS spectra observed from the *x*(Co) = 50.3 SPM film at *H* = 2.0 and 4.6 kOe. The solid lines on the observed spectra (○) give the numerical fits obtained via the CM theory. To show the singlet-doublet structures, the calculated response function *S*(*Q*_//_,*ω*) with small damping of 0.1 ≤ *Γ*/2π ≤ 0.15 GHz is indicated by the solid lines [101].

Here, *n*(*ω*, *T*) is the Bose–Einstein (BE) factor, *R*_zz_, *R*_xx_, *R*_zx_, and *R*_xz_ are the SW-photon coupling constants, and *χ*_zz_, *χ*_xx_, *χ*_zx_, and *χ*_xz_ are the dynamical susceptibilities defined in Equations (79) and (80). The observed BLS spectrum should be compared with a convoluted spectrum between the SW response function $S\left(Q_{//},\omega \right)$ and an instrumental function. We employed the intensity-attenuated Rayleigh peak as the instrumental function (see Figure 32). The solid lines in Figure 32 and Figure 34 display a comparison between the observed spectra and the calculated spectra in the FM state and in the FIM state. 

As shown in Figure 32 and Figure 34, we could fully reproduce the observed BLS spectrum by taking into account only the dipole coupling for SPM spectra with the damping constant of Γ/2π = 2.66 GHz for the *H* = 2.0 kOe spectrum and 2.26 GHz for the 4.6 kOe spectrum. In order to clarify the singlet-doublet structure, we recalculated the response function *S*(*Q*_//_,*Ω*) with a small damping constant of 0.07 ≤ *Γ*/2*π ≤* 0.15 GHz. We adjusted the peak height of the Stokes peak of each spectrum in Figure 34. We found the doublet peaks located at 16.5 GHz and 13.4 GHz at *H* = 2.0 kOe, and at 25.0 GHz and 22.8 GHz at *H* = 4.6 kOe. Because the frequency splitting of 2~3 GHz between the doublet peaks and the peak width were comparable, the FIM doublet actually appears as a single peak in a BLS spectrum. The present numerical analysis reasonably explains why the anti-Stokes peak frequency is higher than the Stokes peak frequency.

**Figure 35:**
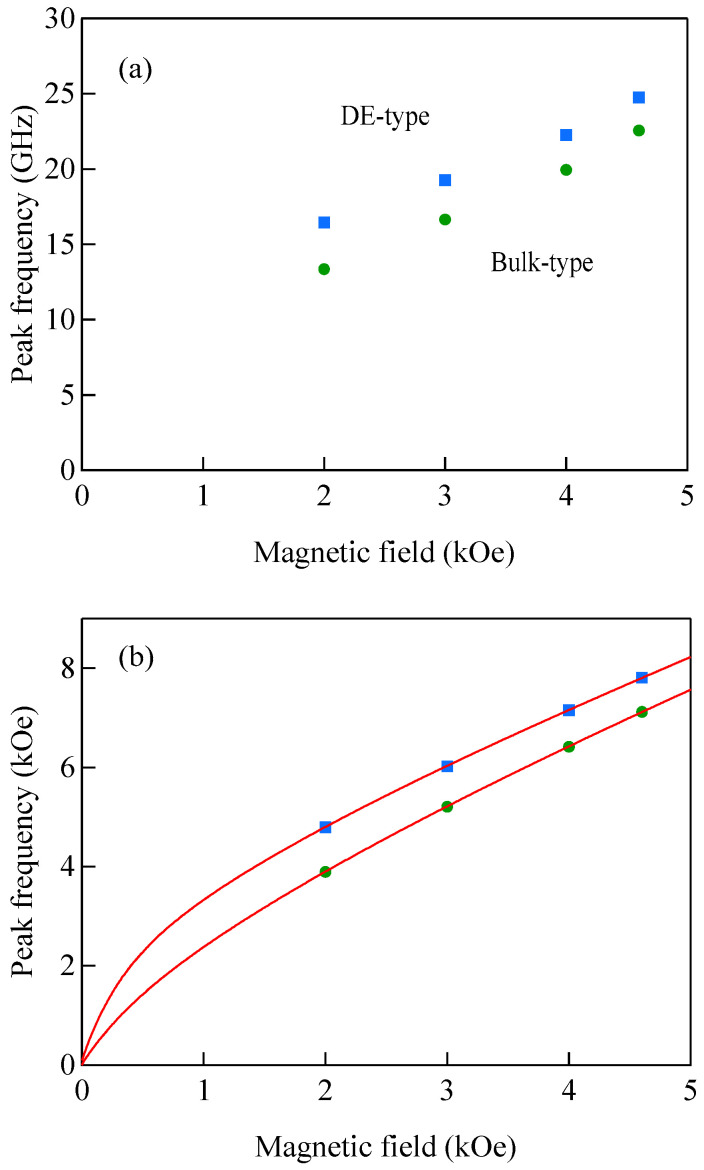
(**a**) FIM peak frequencies obtained from the response function *S*(*Q*_//_,*ω*) with a small damping constant of 0.1 ≤ *Γ*/2π ≤ 0.15 GHz as a function of the magnetic field. (**b**) Field dependence of the FIM excitation frequencies divided by *γ*(*H*)/2*π* as a function of the magnetic field: (●) bulk-type mode and (■) DE-type mode. The solid lines show the frequencies calculated by using the right side of Equations (170) and (171) incorporating the 4π*M*_//_(*H*) result [101].

Figure 35a is the magnetic field dependence of the bulk-type and DE-type peak frequencies in the FIM state determined by the numerical spectrum fitting with small damping constants. 

Because we have confirmed no remanent magnetization at zero magnetic field, the FIM modes should be forbidden at *H* = 0. This means that the FIM mode frequencies should approach zero as the magnetic field approaches zero. On the other hand, the peak frequencies displayed in Figure 35a seem to stay finite even at zero magnetic field. As an attempt to solve this difficulty, we replaced the gyromagnetic ratio *γ* with a field-dependent gyromagnetic ratio *γ*(*H*), and the magnetization *M* with a field-induced magnetization *M*_//_(*H*), which is given by a sum of the Langevin functions. We thus rewrote Equations (51) and (52) as follows:

(170) $$\frac{2\pi \Delta \nu_{B}\left(H\right)}{\gamma \left(H\right)}=H^{1/2}{\left[H+4\pi M_{//}(H)\right]}^{1/2}$$

and

(171) $$\frac{2\pi \Delta \nu_{DE}\left(H\right)}{\gamma (H)}=H+2\pi M_{//}(H)$$

The corrected results are shown as a function of the magnetic field in Figure 35b by filled circles for the bulk-type mode and squares for the DE-type mode. The solid lines show the right-hand side of Equations (170) and (171) with the calculated 4*πM*_//_(*H*) values. Although agreement looks excellent as shown in Figure 35b, it should be recognized that Equations (170) and (171) are for the small-amplitude precession motion around field-induced magnetization *M*_//_(*H*).

So far, I have shown that BLS is a unique technique for investigation of the magnetization dynamics of various opaque magnetic structures in the GHz frequency range. However, most of the BLS studies of opaque magnetic structures have been performed at room temperature, and few BLS studies have been performed at low temperature [12,104,105,106,107]. BLS studies of magnetization dynamics as a function of temperature is quite an interesting subject. As I have already pointed out, BLS intensity from opaque surfaces is much weaker compared to the phonon scattering in transparent materials, even at room temperature. Another inevitable difficulty for scattering experiments stems from the BE factor, which appears in the response function given by Equation (169). For conventional BLS studies performed above 15 K and with a narrow frequency range below 30 GHz, we can reasonably approximate the BE factor as follows:

(172) $$n\left(\omega ,T\right)=\frac{1}{\exp\left(\omega /k_{B}T\right)-1}\approx \frac{k_{B}T}{\omega }$$

This approximation is known as the high-temperature approximation (HTA). Now, BLS intensity is directly proportional to the absolute temperature *T*. It is obvious that weak BLS intensity from opaque surfaces, even at room temperature, gets weaker at lower temperatures. Of course, we can apply an intense laser beam to increase BLS signals. However, the intense laser beam results in a local-heating effect at the beam spot. The local heating effect will be critical for phase transition studies. We must overcome these difficulties to step forward into new frontiers of BLS studies on spin dynamics or magnetization dynamics at low temperatures.

**Figure 36:**
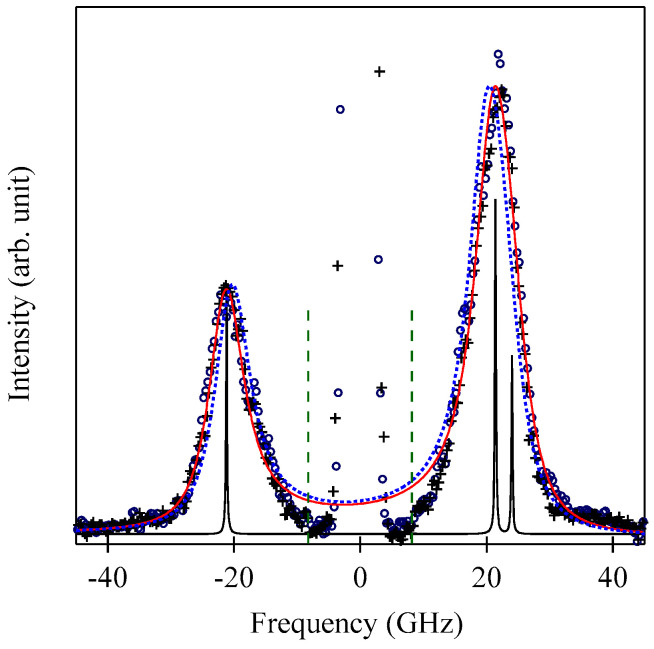
A comparison of the ZFC and FC BLS spectra observed at 20 K and *H* = 4.0 kOe. The solid line shows numerical fits by the CM theory with the ZFC fitting parameters given in the text. The broken line shows the numerical fit with the same ZFC parameters, except *H*_K_ = 0. To show the peak positions, the calculated response function $S\left(Q_{//},\omega \right)$ with a small damping of Γ/2*π* = 0.1 GHz is included with a solid line [108].

Figure 36 shows a comparison of the spectra observed under field-cooling (FC) (**+**) and zero-field-cooling (ZFC) (○) conditions with an external magnetic field of 4.0 kOe at 20 K [108]. 

After spectrum accumulation times over 6 h, we observed FC and ZFC spectra with reasonable signal to noise ratios at 20 K. We found no substantial difference between these spectra, as shown in Figure 36. For more detailed comparisons of these spectra, we properly adjusted the peak heights of the singlet Stokes peak. The vertical broken lines indicate a frequency range in which the AOMs were activated to protect the PMT from optical damage by an intense Rayleigh peak. From our ZFC and FC magnetization measurements at IMR, we estimated the blocking temperature *T*_B_ of our sample to be ~110 K. The FIM changed the temperature dependence from the SPM Langevin type above *T*_B_ to the FM power-law type below *T*_B_. An effective magnetic anisotropy with an easy axis along the applied magnetic field appeared in SPM granular systems below *T*_B_. For convenience’s sake, we assumed a uniaxial-type anisotropy field *H*_K_ (=0.27 kOe). The solid lines in Figure 36 show the calculated ZFC spectrum with the anisotropy field, and the broken lines are the calculated ZFC spectrum without the anisotropy field term. It is obvious that the anisotropy field term improves agreement between the observed and calculated spectra. Although the *H*_K_ term was much smaller than the other fitting parameters, it plays an essential role, as we will discuss later. The inclusion of the *H*_K_ term is equivalent to replacing the external magnetic field *H* with the effective field *H* + *H*_K_ in Equations (170) and (171). In order to display the SPM peak frequencies, we included a calculated spectrum for a small peak-width of Γ/2*π* = 0.1 GHz in Figure 36. 

**Figure 37:**
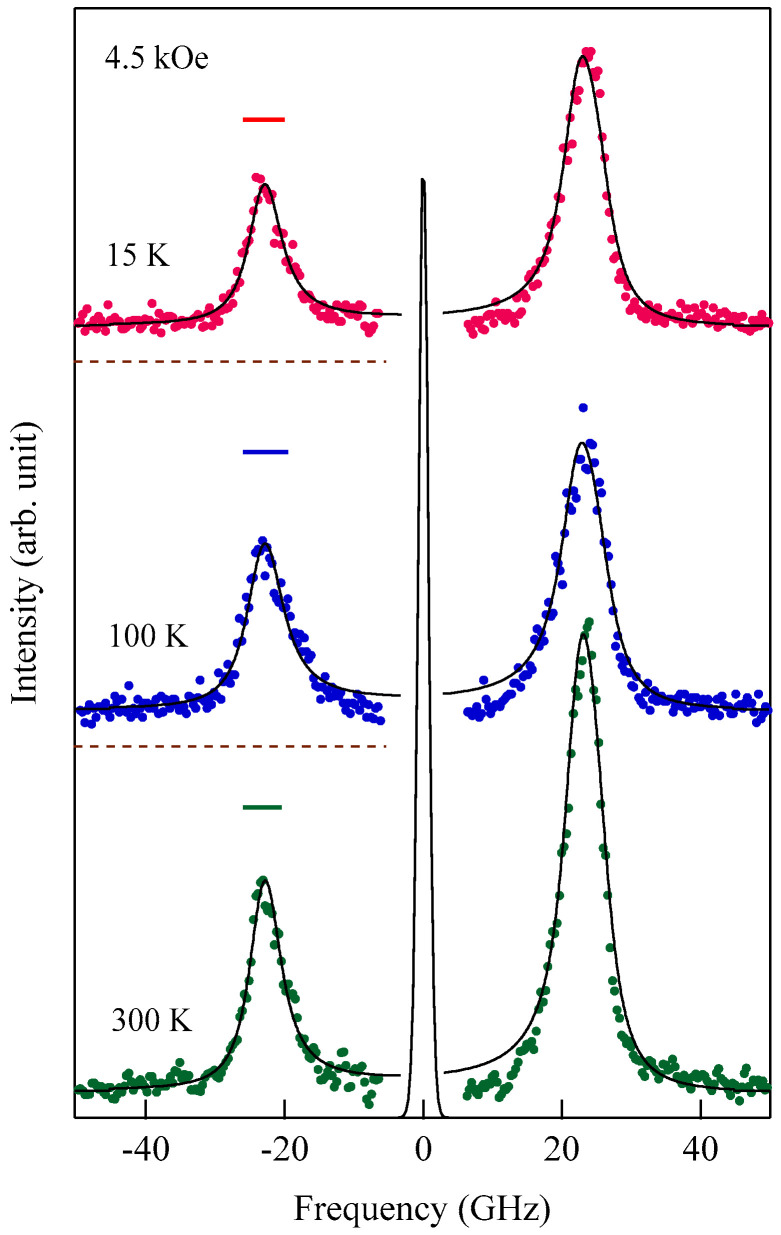
Temperature development of the BLS spectrum in a temperature range between 300 and 15 K under a magnetic field of 4.5 kOe. The baseline of each spectrum, indicated by the broken line, was properly shifted for clarity. The solid lines give the numerical fits obtained by the CM theory, and the horizontal bars show the damping constant of the calculated spectra [108].

Figure 37 shows the temperature development of the BLS spectrum at 300, 100, and 15 K at *H* = 4.5 kOe. 

**Figure 38:**
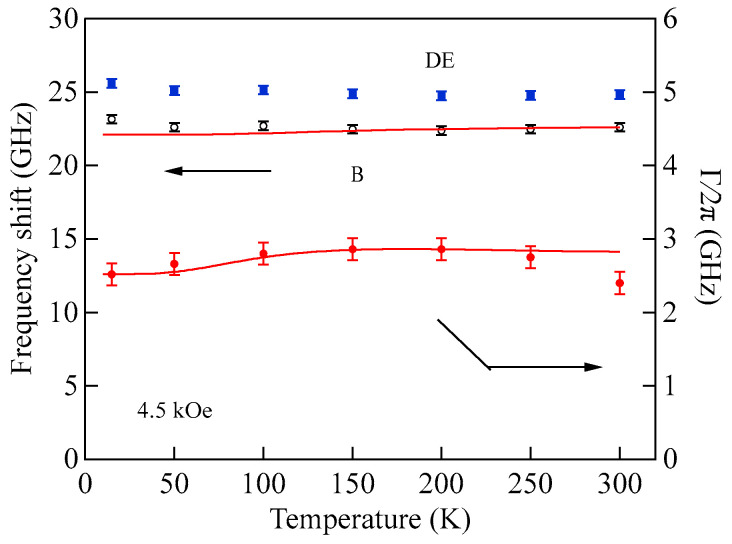
Temperature development of the SPM peak frequencies (Bulk-type (○) and DE-type (■) modes) and the damping constant (●) at 4.5 kOe. The SPM peak frequencies are determined from the calculated response function $S\left(Q_{//},\omega \right)$ with Γ/2*π* = 0.1 GHz as shown in Figure 36. The solid lines show the calculated SPM peak frequency and peak width when taking into account the magnetization relaxation dynamics [108].

I included in Figure 32 an intensity-attenuated (×1/5000) Rayleigh peak for the 300 K spectrum. The solid lines on each spectrum give the calculated BLS spectra. In the calculations, we used a common peak width of Γ/2π for both the bulk and DE-type modes. In spite of this simplification, agreements between the observed and calculated spectra were reasonable. The horizontal bar on each spectrum shows the peak width for each calculated spectrum. We found that the peak width at 100 K was wider than the peak widths at 300 and 15 K. Figure 38 shows the SPM excitation frequencies and the peak width for a magnetic field *H* = 4.5 kOe as a function of temperature. 

**Figure 39:**
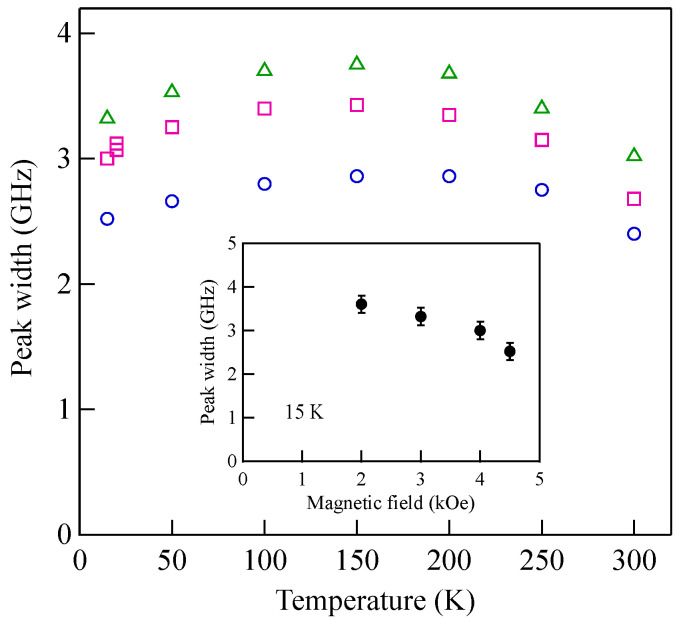
The peak width Γ/2*π* as a function of temperature at *H* = 3.0 kOe (Δ), 4.0 kOe (□), and 4.5 kOe (○). The inset shows a magnetic field dependence of the peak widths at 15 K [108].

The labels B and DE refer to the bulk and DE-type peak frequencies, respectively. These peak frequencies were nearly insensitive to temperature but slightly increased below 50 K. The *H*_K_ term in Equations (170) and (171) gives the increasing frequencies at lower temperatures. To the contrary, the peak width Γ/2*π* exhibited a broad peak centered at ~200 K, as shown in Figure 39. Figure 39 displays the peak width for the external fields of *H* = 3.0 kOe (Δ), 4.0 kOe (□), and 4.5 kOe (○) as a function of temperature. 

The peak width clearly depends on both temperature *T* and the magnetic field *H*. We observed a narrower width for a higher magnetic field. We performed an additional BLS measurement under *H* = 2.0 kOe at 15 K. The inset shows a summary of the magnetic field development of the peak width at 15 K. From the results shown in the inset, we can estimate a limiting value of Γ(0, 15K)/2*π*~4 GHz for the peak width at 15 K and with zero magnetic field. With these observations, it seems to be reasonable to decompose the peak width into the following terms:

(173) Γ(H, T)/2π=φ/2π+ς(H)/2π+ξ(T)/2π

Here, the *ϕ*/2*π* term describes the peak width due to the scattering of the FIM excitations by the nonuniformity of granule sizes (or granule moments) within a film, and it is expected to be temperature- and magnetic field-independent. The *ζ*(*H*)/2*π* term describes the suppression of the incoherent motion of granule moments by the external magnetic field. This term is responsible for our observation of the narrower widths for higher magnetic fields. Finally, the *ξ*(*T*)/2*π* term describes the damping due to couplings between the FIM excitations and another freedom of thermally-excited magnetization dynamics. We will concentrate on this term in our following discussions.

Since the pioneer work by Néel [109], the magnetization reversal dynamics in SPM nanoparticles have been intensively investigated in a wide time scale between 10^2^ s and 10^−13^ s by employing various experimental techniques: magnetization and dynamical susceptibility measurements (with a time scale of 10^2^ to 10^−4^ s), Mössbauer spectroscopy (with a time scale of 10^−8^–10^−9^ s), neutron spectroscopy (with a time scale of 10^−10^–10^−13^ s), and so on [110]. According to the Néel–Brown model, the thermally activated relaxation time is given by [111]:

(174) $$\tau \left(T\right)=\tau_{0}\exp\left(\Delta E/k_{B}T\right)$$

Here, *τ*_0_ is the attempt relaxation time, Δ*E* = *KV* is the activation energy (or anisotropy barrier), *K* is the magnetic anisotropy constant per particle volume, and *V* is the volume of the individual particles. For convenience sake, let us introduce the Debye relaxation model [112] and replace the static FIM 4*πM* in Equation (170) with the dynamical FIM 4*πM*(*ω*) as follows:

(175) $$4\pi M\left(\omega \right)=4\pi M\left(\infty \right)+\frac{4\pi M\left(0\right)-4\pi M\left(\infty \right)}{1+i\omega \tau }=4\pi M\left(\infty \right)+\frac{4\pi \Delta M}{1+i\omega \tau }$$

Here, 4*πM*(∞) and 4*πM*(0) are the limiting high-frequency and low-frequency FIMs, and *τ* is the relaxation time already defined by Equation (174). Then, the bulk-type SPM excitation frequency Δν_B_ given by Equation (170) becomes a complex frequency. For the weak relaxation case (*H* + *H*_K_ + 4*πM*(∞) > >2πΔ *M)*, the real and imaginary parts of the SPM excitation frequency can be readily obtained as follows:

(176) Re(ΔνB)=fB=γ2π(H+HK)1/2(H+HK+4πM(∞))1/2   +γ2π(2πΔM)(H+HKH+HK+4πM(∞))1/211+ω2τ2

and

(177) Im(ΔνB)=ξ2π=γ2π(2πΔM)(H+HKH+HK+4πM(∞))1/2ωτ1+ω2τ2

The peak width *ξ*/2*π* exhibits a maximum at a temperature for which the condition *ω*_B_·*τ*(*T*) = 2*πf*_B_·*τ*(*T*) = 1 is satisfied. In order to determine the parameters *τ*_0_ and *ΔE*/*k*_B_ in Equation (174), we should perform at least two BLS measurements with different frequencies, *f*_1_ and *f*_2_, by simply changing the magnetic field. In the following discussions, we will use the GHz unit for these frequencies. When we observe the peak-maximum at temperatures *T*_1_ and *T*_2_, then we can readily calculate the relaxation parameters *τ*_0_ and *KV*/*k*_B_ as follows:

(178) $$\frac{KV}{k_{B}T_{1}}=\frac{T_{2}}{T_{2}-T_{1}}\ln\left(\frac{f_{2}}{f_{1}}\right)=\frac{1}{1-\beta }\ln\left(\frac{f_{2}}{f_{1}}\right)=\ln{\left(\frac{f_{2}}{f_{1}}\right)}^{\frac{1}{1-\beta }}$$

and

(179) $$\tau_{0}=\frac{1}{2\pi f_{1}}\exp\left(-\frac{KV}{k_{B}T_{1}}\right)=\frac{1}{2\pi f_{1}}{\left(\frac{f_{1}}{f_{2}}\right)}^{\frac{1}{1-\beta }}=\frac{1}{2\pi }{\left[\frac{f_{1}{}^{\beta }}{f_{2}}\right]}^{\frac{1}{1-\beta }}\times 10^{-9}\;s$$

Here, we have defined *β* = *T*_1_/*T*_2_. Because our BLS measurements have been performed at limited temperatures, we adapted the least-squares fourth-order polynomial fitting to determine the peak-maximum temperature. Therefore, our following discussions are limited to qualitative ones. We obtained two sets of the parameters: (*f*_2_ = 22.1 GHz, *T*_2_ = 178 K) from the 4.5 kOe results and (*f*_1_ = 16.5 GHz, *T*_1_ = 143 K) from the 3.0 kOe results. In fact, we also have the excitation frequencies: *f*_2_ = 22.4 GHz and *f*_1_ = 16.9 GHz. Because these frequencies involve the relaxation contribution given by Equation (176), we corrected the contributions. Utilizing Equations (178) and (179), we obtained a set of relaxation parameters: *τ*_0_ = 2.2 × 10^−12^ s and *KV*/*k*_B_ = 213 K. It is interesting to compare the present relaxation parameters with the ones for other nanogranular systems and also physically different structural relaxation systems. Neutron scattering from ~15 nm hematite particles detected a quasielastic peak due to SPM relaxation and propagative precession peaks [113]. From temperature development of the quasielastic peak, the attempt relaxation time of *τ*_0_ = 7 × 10^−12^ s and the anisotropy barrier *KV*/*k*_B_ = 500 ± 200 K were determined. Linderoth et al. obtained *τ*_0_ = 2 × 10^−12^ s and *KV*/*k*_B_ = 428 ± 29 K for Fe-C particles by combining the magnetization with the Mössbauer techniques [114]. These values are in the same order with the present values of *τ*_0_ = 2.2 × 10^−12^ s and *KV*/*k*_B_ = 213 K. Much longer attempt relaxation times of ~10^−10^ s have been reported on α-Fe, α-Fe_2_O_3_, and γ-Fe_2_O_3_ nanoparticle systems [110]. For comparison, we obtained *τ*_0_~4.0 × 10^−18^ s and ∆*E* = 0.55 eV (=6380 K in the temperature scale) for P[VDF-TrFE] copolymer films [115], and *τ*_0_~1.3 × 10^−15^ s and ∆*E* = 0.28 eV (=3250 K) for glass-former propylene glycol [116]. It is very interesting to note that the attempt time of *τ*_0_~10^−12^ s for magnetic relaxation is three orders or more long, and that the activation energy (height of the potential barrier to jump) is one order or more lower than the barrier height for the structural relaxation. The characteristic features of SPM magnetization relaxation can be summarized as slow motion within a shallow potential minimum.

Here, we return to Equation (173). At 15 K, the magnetization relaxation time *τ* in Equation (174) is calculated as *τ* = 3.23 × 10^−6^ s. At the BLS frequency of *f*_B_~20 GHz, the condition 2π*f*_B_*τ* >> 1 is fully satisfied. This condition means that the SPM excitation frequency is too fast to couple with the magnetization relaxation process. Therefore, we can set *ξ*(15 K)/2*π* = 0 in Equation (173). Now, assuming that the φ/2π+ς(H)/2π term in Equation (173) is independent of temperature at *H* = 4.5 kOe, the relaxation amplitude is given by

(180) $$\frac{\gamma }{2\pi }\left(2\pi \Delta M\right){\left(\frac{H+H_{K}}{H+H_{K}+4\pi M\left(\infty \right)}\right)}^{1/2}=2\frac{\Delta \Gamma_{R}}{2\pi }=2\left(\frac{\Gamma \left(178\;K\right)}{2\pi }-\frac{\Gamma \left(15\;K\right)}{2\pi }\right)=2\frac{\xi (178\;K)}{2\pi }$$

When we put the available constants ΔΓ_R_/2*π* = 0.35 GHz, *γ*/2*π* = 3.11 GHz/kOe, *H*_K_ = 0 kOe, *H* = 4.5 kOe, and 4*πM*(∞) = 6.76 kG into Equation (180), we obtain 2*π*Δ*M* = 0.36 kG and the relaxation amplitude of 0.70 GHz. Finally, we obtain the SPM frequency and the peak width *ξ*(*T*)/2*π* for the bulk-type mode as follows:

(181) $$f_{B}=22.1+0.70\frac{1}{1+\omega^{2}\tau^{2}}$$

and

(182) $$\frac{\xi }{2\pi }=2.52+0.70\frac{\omega \tau }{1+\omega^{2}\tau^{2}}$$

The solid lines in Figure 38 are the calculated SPM excitation frequency *f*_B_ and the peak width *ξ*/2*π* at *H* = 4.5 kOe as a function of temperature by using Equations (181) and (182). The magnetic relaxation model qualitatively reproduces the observed temperature development of the peak width, except for the higher temperatures above 250 K. The calculated frequency below 100 K was lower than the observed frequencies. Because we have not included the temperature dependence of the *γ*/2*π* constant and the *H*_K_ term in Equation (181), it is possible to improve agreement between the observed and calculated frequencies. We can also qualitatively reproduce the results for *H* = 3.0 and 4.0 kOe with the above relaxation parameters by changing the relaxation amplitude. As we have already discussed, we can ignore the *ξ*/2*π* term at 15 K in Equation (173) because of the condition *ω*_B_*τ* >> 1. Then, we can rewrite Equation (173) as follows:

(183) Γ(H, 15 K)/2π≅ϕ/2π+ς(H, 15 K)/2π

The inset in Figure 39 shows the magnetic field dependence of the peak width Γ(*H*, 15 K)/2*π*. As the magnetic field increased, the peak width decreased. This behavior can be attributed to the magnetic field dependence of the *ζ*(*H*, 15 K)/2*π* term. Unfortunately, the highest magnetic field available in our BLS system with the closed-cycle refrigerator is not enough to fully separate these *ϕ*/2*π* and *ζ*/2*π* terms in Equation (183). However, as a rough estimation, we obtained *ϕ*/2*π* ≈ 2 GHz and *ζ*(0 kOe, 15 K)/2*π* ≈ 2 GHz, respectively. 

So far, I have demonstrated in this granular section that the BLS technique involves higher potential for investigation of fast magnetization dynamics. In the Co-Al-O system, we investigated the dynamics in the frequency range of around 20 GHz. However, it is easy to extend the frequency range below several GHz and over a few hundred GHz for the BLS technique by utilizing a tandem FPI. Furthermore, we can adjust the SPM excitation frequencies by applying an appropriate external magnetic field according to Equations (170) and (171). I would like to emphasize these advantages of the BLS technique in the magnetization dynamics study of SPM materials. 

**Figure 40:**
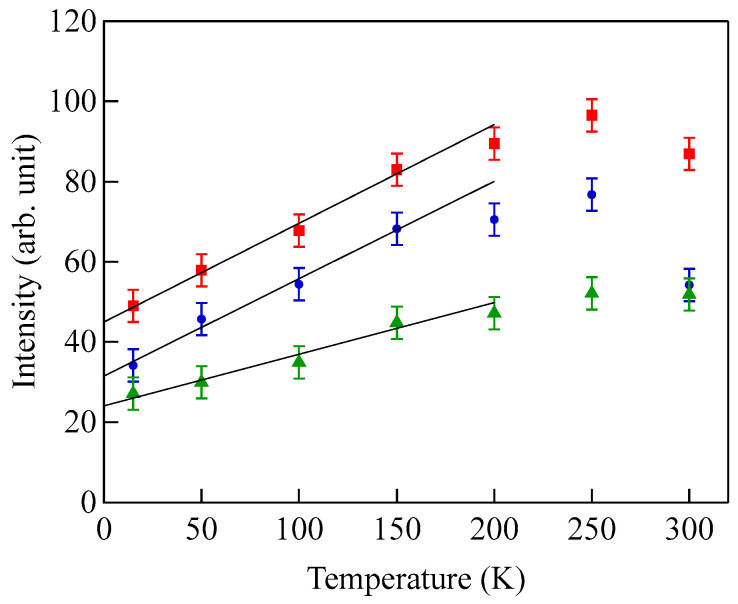
The peak intensities of the SPM excitations as a function of temperature: (●) bulk-type anti-Stokes peak, (■) bulk-type Stokes peak, and (▲) DE-type peak. The solid lines are the least-squares fits to the linear function *I*(*T*) = *A* + *B*·*T* below 150 K [108].

Figure 40 shows the peak intensities at *H* = 4.5 kOe obtained from the fittings and normalized by the total spectrum accumulation time as a function of temperature. 

Although all peak intensities monotonously decreased as temperature decreased, the peak intensities at 15 K kept about 50% of the highest intensities observed at 250 K. It is important to note that the BE factor in Equation (169) is an inevitable sequence of the quantum-mechanical fluctuation-dissipation theorem [117] and independent of the details of the real physical systems. The solid lines in Figure 40 show the least-squares fits below 150 K to a linear function of temperature given by *I*(*T*) = *A* + *BT*. The linear function well describes the observed temperature dependence of the peak intensities. Now, we rewrite the linear function in the following form.

(184) I(T)/T=A/T+B

Here, *I*(*T*)/*T* is proportional to Equation (169) divided by the BE factor. Because the optical properties and the dynamical susceptibilities appearing in Equation (169) are essentially independent of temperature, we can explain the B term. However, we cannot explain the existence of the *A*/*T* term within the framework of the single-site magneto-optic coupling mechanism already discussed. Nevertheless, the *A*/*T* term in Equation (184) is the most crucial in our present results. When we use the least-squares parameters *A* = 45.0 and *B* = 0.246 for the Stokes peak (■), it is obvious that the *A*/*T* term dominates the BLS scattering intensity below 100 K. At 15 K, the *A*/*T* term is more than ten times larger than the *B* term. This can be the reason why we could observe relatively intense scattering even at 15 K. 

Non-*T*-proportional behaviors of SW BLS intensity have already been reported in several conductive FM materials, for example, semiconductor EuS and EuO single crystals [104], a FM/AFM Co (2.8 nm)/CoO (0.7 nm) bilayer thin film [106], and so on. These materials are magnetically quite different from the present SPM Co-Al-O granular system. Because we cannot explain the *A*/*T* term in Equation (184) within the phenomenological description of the conventional single-site magneto-optical coupling theory given by Equations (14)–(18), some collective motion of electrons might contribute to the SW light scattering in conductive FM materials. In order to elucidate the light scattering mechanism, more extensive BLS studies for various conductive magnetic materials are strongly recommended.

### 5.6. SWs in Confined Structures and Devices

Unfortunately, I had no chance to perform BLS measurement in this research area. This area is closely related to the rapidly growing magnonics and spintronics fields, and is a promising area for the next generation of SW BLS study. For readers interested in this field, I give a few of recent references [118,119,120]. 

## 6. Summary

Since my SW BLS reports on CoZr and FeSi films published in 1989, I spent more than 30 years in the field of SW BLS. BLS as an optical technique to determine a set of the basic magnetic constants of a magnetic thin film was fully established during the period, as I have presented in this review. Now, the BLS technique is recognized as one of the best techniques not only to investigate the SWs and the magnetization dynamics in the GHz frequency range, but also to characterize magnetic interactions induced in various artificial magnetic structures. There is no doubt that developments both in the sample preparation techniques and the BLS technique will go hand in hand to open new frontiers of functional devices as well as basic material sciences. When I started my SW BLS research in the middle of the 1980s, quasi-two-dimensional magnetism was just a textbook subject, and I never imagined that I could measure SWs in such a thin film of a few monolayers in thickness. However, the MBE technique made it possible.

Because of the sensitivity and flexibility of the light scattering technique, the importance and usefulness of the BLS technique in materials science and engineering is still growing and growing. The downsizing of magnetic devices will result in magnetic instability due to direct or indirect interactions between magnetic elements. The microfocused BLS technique and micromagnetic simulation can be applied to characterize interactions between the magnetic elements. BLS detection of the spin current and the spin accumulation in spintronics devices will be another interesting challenge.

Through my research career in the BLS field spans over 45 years, including structural and ferroelectric phase transitions, low temperature liquids, glass formers, and SAWs and SWs from opaque surfaces, I learned many new physics, ideas, and the importance of the challenges when going into a new research field. The BLS study from opaque surface was exactly a challenge for me. When I was asked to examine BLS from SPM granular samples, to speak frankly, I had no assurance that I could observe BLS signals because of my preconception that short range interactions such as the exchange coupling were necessary to generate magnetic excitations. Fortunately, my idea was completely wrong as I have presented in this review, and BLS can be a new tool to investigate the magnetization dynamics of SPM granular materials.

## Figures and Tables

**Table 1:** Magnetic constants of Co_85_Nb_12_Zr_3,_ bcc Fe, and hcp Co films used for the SW frequency calculations.

Magnetic Constants	Co_85_Nb_12_Zr_3_	bcc Fe	hcp Co
*γ*/2*π* (GHz/kOe)	2.98	2.09	2.17
4*πM*_B_ (kG)	10.0	21.5	17.8
4*πM*_S_ (kG)	10.0	21.5	17.8
*D* (10^−9^ Oe·cm^2^)	2.47	2.34	3.39
*K*_1_ (10^6^ erg/cm^3^)	−	0.45	3.45
*K*_2_ (10^6^ erg/cm^3^)	−	−	0.9

## Data Availability

Data are contained within the articles.
